# Evaluation of a piloted digital reproductive health registry in Jordan to improve mother and child health

**DOI:** 10.1186/s12978-025-01995-2

**Published:** 2025-05-31

**Authors:** Mirwais Amiri, Majd Al-Soukhni, Manal Tahtamouni, Mohannad Al Nsour

**Affiliations:** 1 The Eastern Mediterranean Public Health Network (EMPHNET), Shmeisani, Abdallah Ben Abbas Street, Bldg No. 42, Amman, Jordan; 2 Independent Consultant, Amman, Jordan

**Keywords:** Electronic registry, Health records, Reproductive health, Maternal and child health

## Abstract

**Background:**

Primary data on antenatal care services in Jordan are stored in diverse systems among hospitals and Mother & Child Health (MCH) centers. The resulting information flow gaps between healthcare levels challenge the quality and continuity of MCH care. To close these data and care delivery gaps, a *harmonized* Reproductive Health Registry (*h*RHR) was designed and piloted to bring data in a consistent/digital/readily accessible format and enhance the current health information system. Our study evaluated the perceptions on the newly developed *h*RHR’s improvement of the delivery of sexual and reproductive health (SRH) services in 19 healthcare centers where the *h*RHR was piloted.

**Methods:**

We utilized a mixed methodology (qualitative and quantitative assessments). Three tools were used for Key Informants/stakeholders, services providers, and women.

**Results:**

A total of 13 SRH stakeholders, 37 service providers, and 855 women/service users participated in this evaluation. All SRH stakeholders agreed that the *h*RHR is responding to a need for digitalization of routine MCH data, medical files, and reports. They all agreed that the synchronization with the currently used Computerized Patient Record System (CPRS) and the *h*RHR Web-based application will enhance the expansion and needed upgrades in the future. For service providers, 73% were satisfied with the system, 78.4% were willing to keep using it, and 81.1% indicated that the *h*RHR is highly useful to health providers. For women/service users, 89.2% agreed/strongly agreed that the new system improves the confidentiality and privacy of their health information, and 89.7% agreed/strongly agreed that the new system allows health staff to easily access patient information. There were also foreseeable challenges including fragmentation of electronic data management, lack of connectivity across health services, limited ownership, dependence on external funding as impeding factor for scalability, poor IT infrastructure still disrupting service in MCH centers and affecting data entry, and limited electronic connectivity/reporting access impacting the continuity of care.

**Conclusion:**

The new *h*RHR has a high acceptance level among stakeholders, health providers and women using MCH services. The evaluation showed that the system improved documentation of data, decreased time, and effort of data reporting and retrieval, and improved access to patient data.

**Supplementary Information:**

The online version contains supplementary material available at 10.1186/s12978-025-01995-2.

## Plain Language Summary

In Jordan, health information about the services provided to pregnant women is scattered across different systems in various hospitals and centers, leading to gaps in information flow and challenges in providing consistent care to those women. Therefore, a new system called the *harmonized* Reproductive Health Registry (*h*RHR) was created to organize and improve access to this data in one easy-to-use digital format. To see how this new system affected the healthcare provided to women, the new system was implemented and tested in 19 health centers. We used different methods, like interviews and surveys, to collect feedback from 13 high level personnel involved in sexual and reproductive health, 37 healthcare workers who were using the new system, and 855 women who benefited from the provided services. All participants expressed their consensus on the new system's effectiveness in organizing information and medical records digitally. The majority of healthcare workers expressed satisfaction and a willingness to continue using it, and most women believed it enhanced the privacy of their health information while facilitating easier access for staff. There were also foreseeable challenges for the implementation and scaleup of the new electronic data management system due to the poor/limited technology infrastructure and existing dependence on external funding. In summary, the new system received positive feedback overall and was perceived as an improvement for quicker and more efficient recording and retrieval of patient information services.

**Supplementary Information:**

The online version contains supplementary material available at 10.1186/s12978-025-01995-2.

## Résumé

**Contexte:**

Les données primaires sur les services de soins prénataux en Jordanie sont stockées dans les divers systèmes des hôpitaux et des centres de santé maternelle et infantile (SMI). Les lacunes dans les flux d’informations qui en résultent entre les différents niveaux de soins de santé compromettent la qualité et la continuité des SMI. Pour combler ces lacunes en matière de données et de prestation des soins, un registre de santé reproductive *harmonisé* (*h*RHR) a été conçu et mis à l’essai afin de rassembler les données dans un format cohérent, numérique et facilement accessible et d’améliorer le système d’information sur la santé actuel. Notre étude a évalué les perceptions de l’amélioration de la prestation de services de santé sexuelle et reproductive (SSR) grâce au *h*RHR nouvellement développé dans 19 centres de santé où le *h*RHR a été mis à l’essai.

**Méthodes:**

Nous avons utilisé une méthodologie mixte (évaluations qualitatives et quantitatives). Trois outils ont été utilisés pour les informateurs/intervenants clés, les prestataires de services et les femmes.

**Résultats:**

Au total, 13 intervenants de la SSR, 37 prestataires de services et 855 femmes/utilisatrices de services ont participé à cette évaluation. Tous les intervenants de la SSR ont convenu que le *h*RHR répond à un besoin de numérisation des données de routine de la SMI, des dossiers médicaux et des rapports. Ils ont tous convenu que la synchronisation avec le système informatisé de dossiers médicaux actuellement utilisé et l’application Web *h*RHR facilitera l'expansion et les améliorations nécessaires à l’avenir. Pour les prestataires de services, 73 % étaient satisfaits du système, 78,4 % étaient prêts à continuer à l’utiliser et 81,1 % ont indiqué que le *h*RHR est très utile aux prestataires de soins de santé. Pour les femmes et les utilisatrices de services, 89,2 % étaient d’accord ou tout à fait d’accord pour dire que le nouveau système améliore la confidentialité et la protection de leurs renseignements médicaux, et 89,7 % étaient d’accord ou tout à fait d’accord pour dire que le nouveau système permet au personnel de santé d’accéder facilement aux renseignements sur les patients. Il y avait également des défis prévisibles, notamment la fragmentation de la gestion des données électroniques, le manque de connectivité entre les services de santé, la propriété limitée, la dépendance à l’égard du financement externe en tant que facteur entravant l’évolutivité, la mauvaise infrastructure informatique qui perturbe toujours le service dans les centres de SMI et affecte la saisie des données, et la connectivité électronique limitée/l’accès aux rapports qui affecte la continuité des soins.

**Conclusion:**

Le nouveau système *h*RHR bénéficie d’un niveau d’acceptation élevé parmi les parties prenantes, les prestataires de soins de santé et les femmes utilisant les services de SMI. L’évaluation a montré que le système a permis d’améliorer la documentation, de réduire le temps et les efforts consacrés à la création de rapports et à la récupération des données et d’améliorer l’accès aux données des patients.

## Background

Sexual and Reproductive health (SRH) is a state of complete physical, mental, and social well-being and not merely the absence of disease or infirmity, in all matters relating to the reproductive system and to its functions and processes [1, 2]. The concept of “Reproductive Rights” refers to a package of rights and freedoms related to reproduction and reproductive health that are required to be legally and societally protected [3, 4]. These rights encompass issues surrounding gender and the right to respectful and quality SRH services irrespective of the medical insurance status of service users or their nationality (in our case, refugee status). Sexual and reproductive health are linked to several human rights such as the right to life, the right to health, the right to privacy, the right to information, the prevention of discrimination and others [5].

Within its post-2014 Plan of Action, Jordan renewed its own commitment to the importance of taking measured actions for promoting sexual and reproductive health, including family planning programs, through the provision of quality information and services [6]. Provision of quality sexual and reproductive health is a main pillar of the Jordanian government’s commitments to many signed international treaties and agreements. Among the most important of these commitments are the Program of Action of the International Conference on Population and Development/Cairo—1994, which was renewed during the Nairobi Summit in 2019, and the 2030 Agenda for Sustainable Development [7, 8], in particular, the third goal of the Sustainable Development Goals, to “ensure that all people enjoy healthy lifestyles and prosperity at all ages and that all the right to the enjoyment of the highest attainable standard of physical and mental health” [9].

Measuring progress against such commitments requires comprehensive health information systems that capture the right information, at the right place, and that in turn ensure that information can be used for decision-making. In Jordan, primary data on antenatal care are collected in the public sector at the mother and child health (MCH) centers, whereas perinatal (intrapartum and postpartum) care data are for their part collected and entered in public or private hospitals where a woman delivers her baby. Since MCH services are delivered free of charge to all women irrespective of their nationality or medical insurance status, many women seek antenatal care at MoH H centers. However, when it comes to the women’s delivery, which cannot be provided at those centers, women have to give birth at hospitals (public or private). Thus, a woman’s medical records might not be connected between the hospital she chooses for delivery and the center where she received antenatal care. These data are gathered in both paper and electronic formats. As for the Mafraq Governorate, about 25% of the health facilities were automated using the Computerized Patient Record System (CPRS) by the Electronic Health Solutions (EHS) Company (also called EHS-Hakeem). The mentioned company provides CPRS to a similar proportion of health facilities across other governorates of the Kingdom. It is also worth noting that, even in the CPRS-automated centers, the data could not generate aggregated reports. Thus, all MCH health facilities, including the ones automated by EHS-Hakeem, collected aggregated data manually on paper files/folders to generate daily, weekly, and monthly reports for the Ministry of Health.

Without an integrated electronic health information system in many settings, paper-based registers and patient folders lead to ineffective health information management and related health services planning, and ultimately to poor health outcomes. Timely and actionable data, needed for health service delivery, program management and policy development, are incomplete and limited. As a result, extraction of data from disparate paper files results in poor quality data and underutilized health information [10].

It is recommended by the World Bank, World Health Organization (WHO), US Agency for International Development (USAID), and other global organizations that all countries have comprehensive databases and electronic health information systems that support quality health service delivery and allow providers to follow up with clients in a timely manner, whether within the same or different sectors [11]. The Global Roadmap for Health Measurement and Accountability considers integrated electronic health information systems as key to obtaining continuous, sustainable, and secure information exchanges at different levels of the health system [11]. Electronic health registries represent integrated systems that can ensure an effective data collection for health workers to seamlessly follow clients along the continuum of care and across different levels of care [12].

To close data and care delivery gaps, a *harmonized* Reproductive Health Registry (*h*RHR) was designed and piloted to organize and improve access to data in one easy-to-use digital format that will bring data in a consistent and readily accessible manner and enhance the current health information system. The hRHR was implemented in 19 MCH centers of Mafraq where MCH data collection was not yet automated by EHS-Hakeem (i.e., where MCH data collection was fully paper-based). The *harmonized* Reproductive Health Registry (*h*RHR) is a global initiative introduced by the World Health Organization in 2013 to improve data quality and data collection techniques for maternal and child health. Led by the Department of Reproductive Health and Research (RHR)/World Health Organization and the Norwegian Institute of Public Health (NIPH), the overall aim of the *h*RHR Initiative is to reduce maternal and infant mortality by facilitating the introduction of reproductive health registries for better governance, targeting, health surveillance and accountability of public health initiatives in reproductive health [13].

Given the need for an e-registry for enhancing MCH data in Jordan, the *h*RHR for Jordan was developed, introduced, and piloted to bridge the gap in data accessibility for beneficiaries and health service providers. The *h*RHR is a web-based application to institute a unified approach for MCH data entry and retrieval by service providers, offering more comprehensive medical records for mothers and children across the country. The *h*RHR was developed as the “lite” version of the system currently used in Jordan, the Computerized Patient Records System (CPRS), and is therefore also referred to as the “CPRS-Lite”. Launched in 2009, CPRS is a platform for sharing medical data across different healthcare providers. It is used to store patient records and allows healthcare providers to access patient information electronically. Thus, it is intended to help in reducing medical errors and improve patient safety by ensuring that all relevant information is available at different points of care. The *hR*HR, on the other hand, was developed in 2019–2020 to run in synchronization with CPRS. The reason to develop it as a separate, yet integrated, system is to make it more flexible and responsive for any needed changes as per the evolving MCH care services requirements, including generating aggregated reports, the flexibility to add more indicators as needed, and the minimum infrastructure it requires. The main difference between the CPRS and *h*RHR (CPRS-Lite) is that the *h*RHR collects all MCH data exactly as previously collected by hand on the MoH-authorized cards, which helps in maintaining consistent health records across all service points. Unlike the CPRS, the “Lite” web-based *h*RHR is easily accessible via mobile devices and provides connectivity from any location with internet access. Moreover, it is automatically linked to the country-wide CPRS, creating a more integrated healthcare information network. Synchronized with the core CPRS, the *h*RHR system facilitates streamlined data entry and utilization, enhancing accessibility for authorized users. Its innovative reporting feature generates comprehensive statistical counts for MCH indicators, empowering decision-makers and elevating overall MCH service quality.

This implementation research project, led by the Eastern Mediterranean Public Health Network (EMPHNET), with the Jordan Ministry of Health, aimed to examine the benefits of implementing the *h*RHR to improve sexual and reproductive health (SRH) services and referral systems between different levels of service delivery in Jordan. Mafraq was the chosen place for the implementation, as it is the second largest population area in Jordan, hosting the largest refugee camp for Syrian refugees, Zaatari, and is the third largest area that hosts off-camp Syrian refugee population [14].

In this evaluation, we aimed to study the new *h*RHR intervention in Jordan and measure the success (or failure) of this pilot in Mafraq Governorate (located in the northeastern part of the Kingdom) and to know if it would live up to the expectations when it is rolled out on a national scale. As with any system, the acceptability, usefulness, sustainability, feasibility, simplicity, and other features of its implementation are critical to its uptake and adoption.

The objectives of the study were:

1. To analyze the strengths and weaknesses of the *h*RHR
2. To assess the effect of *h*RHR on improvement of SRH data management and reporting
3. To provide recommendations for improvement for the second phase of the project (national expansion)

## Methods

At the onset of the implementation of the new *h*RHR intervention, a high-level Technical Steering Committee (TSC) was established which comprised of a maximum of 12 members, including high-level MoH managers and other MCH & ICT stakeholders. This committee supported the implementation of the project by providing guidance leading to the development of project components and technical principles, operational policies, and processes that ensure effective functioning. The committee played a fundamental role in facilitating the implementation of project activities, considering the policies, technical principles, and systems adopted by the MoH from the project kick-off till its endpoint evaluation. The TSC also endorsed this study, which utilized a mixed methodology of data collection (qualitative and quantitative assessments) to respond to the above specific objectives. The use of mixed methodology also allowed for looking for other aspects of the system such as human rights and gender perspectives. Quantitative data was collected through a self-administered questionnaire for health providers, and qualitative data was collected using semi-structured interviews with SRH services users (women benefiting from the system) in the MCH centers, as well as through focus group discussions (FGDs) with key informants consisting of MCH and IT managers and supervisors. Various outcomes were examined, including acceptability, usefulness, sustainability, governance/data security, feasibility, simplicity, human rights, and gender, to ensure the system alignment with user needs, ethical standards, and practical considerations within the healthcare environment.

### Study sample size

A purposive sampling approach was used to identify key informants/stakeholders for FGDs. The KIs list of 13 stakeholders (high level personnel involved in sexual and reproductive health) was carefully selected to offer diverse perspectives on the new *h*RHR from various crucial angles within the healthcare system. Those included MCH managers, IT managers, midwives’ supervisors and others. “Data saturation” was the main factor determining the sample size (i.e., number to be interviewed) for this part of the assessment.

For the health providers self-administered questionnaires, the whole study target population (i.e., all 37 *h*RHR users from all 19 health facilities) were targeted in the evaluation.

Finally, for the SRH service users, a randomly selected sample size of over 850 individuals from 19 centers was calculated for a 95% confidence level and a 5% margin of error, which would also account for (i.e., was inclusive of) the non-response.

### Study tools

Three tools for data collection were developed and were discussed and endorsed by the high-level TSC. The data collection tools were:

1. FGDs with key informants/stakeholders. The questionnaire reflected on different aspects, including overall impression, strengths and weaknesses of the system, effect on quality and availability of data collection and reporting, comprehensiveness, effect on referral mechanism, sustainability, and general recommendations for improvement.
2. Self-administered health providers questionnaire (*h*RHR users) reflected on different aspects, including acceptability, usefulness, sustainability, feasibility, simplicity, human rights, and gender sensitivity.
3. Questionnaires for SRH users in MCH centers reflected on different aspects including waiting time, privacy and confidentiality of data, time allocated by service providers, accessibility of data by health providers.

All study tools included questions on such aspects of the system as human rights and gender perspectives. The three tools were translated into Arabic language including its back translation for quality and standardization of the tools to the Jordanian culture, language, and cultural consideration. The service providers’ and service users’ questionnaires were automated using KoBoToolbox to be used online.

### Data collection

Data was collected between the period of September 20 to October 20, 2021. First, for key informants/stakeholders, two FGDs—of 6–7 participants each—were conducted (Table 1). Secondly, for service providers, a focal point from Al Mafraq Health Directorate was assigned to ensure the distribution of the questionnaires and collection of data from SRH service providers. The questions were sent electronically to each of the health providers where they responded individually. And finally, for service users (women), five researchers who were trained on survey administration, data collection, and gender sensitivity visited the 19 MCH centers to carry out interviews. Women who were visiting the piloted MCH centers on the days of data collection were approached by the researchers and the center health providers to participate in the evaluation. Women respondents were targeted randomly. Their selection took into consideration the average monthly patient load per center divided by the number needed to be interviewed to account for the sampling interval in each center. This selection approach resulted in the inclusion of women from different nationalities (Jordanian, Syrians and others), women receiving various MCH services, and women being aware or unaware of the presence of the new electronic system on a random basis.

**Table 1:** Data collection methods and participants distribution

Data type	Data collection method	Number of participants
Qualitative	Two focus group discussions	13 stakeholders
Quantitative	Self-administered questionnaire	37 Service providers 855 Service users

### Data management and analysis

Both quantitative and qualitative data were collected in Arabic and went under strict measures for data quality and integrity, including data cleaning and validation for its accuracy, validity, and relevance. Quantitative dataset was searched for and cleaned of duplicates, typos and incorrect entries, implausible values, and other issues. As for qualitative data, all transcripts were checked by separate transcribers and spot-checked according to their timestamps in the recordings.

Quantitative data was analyzed using Statistical Package for the Social Sciences (SPSS) for all variables included in the assessment. As for the qualitative data, content and thematic analyses were conducted using Macro-enabled Microsoft Word plug-in for extracting comments [15] to extract coded data (codes and categories) into Excel for content analysis and generating themes.

Coding and re-coding was done through several iterative processes to systematically classify data and identify key categories of issues under the discussion. This was accompanied by thematic analysis which focused on the search of already identified themes and generation of new themes from the dataset.

## Results

### I. FGDs with Key Informants/stakeholders

Two FGDs were conducted with 13 stakeholders representing different entities who were directly involved in the need assessment, planning, execution, and provision of support during the implementation of the *h*RHR project (Table 2).

**Table 2:** FGDs list of participants

Participants
FGD (1): (7 Participants)
- Director of Woman and Child Directorate/MoH
- Manager of FP supply unit/MoH
- Director of Quality and Institutional Development Directorate/MoH
- Director of IT department/MoH
- Three representatives of Technical/Administrative support units/EHS-Hakeem
FGD (2): (6 participants)
- Director of Mafraq Health Affairs Directorate
- Head of IT unit/Mafraq
- Midwife-FP supply coordinator/Mafraq
- Midwife-MCH supervisor/Mafraq
- Manager of MCH section/Mafraq
- Midwife/Mafraq

#### Overall assessment

All participants in the FGDs agreed that the new system is responsive to the need for digitalization of routine MCH data, medical files, and reports. They all agreed that the synchronization between the currently used CPRS and the *h*RHR Web-based application will enhance the expansion and needed upgrades in the future. They all agreed, and the feedback from the users revealed this as well, that the system is user-friendly and is based on the files and reports usually used at the level of the health facility. However, reporting and defining indicators continue to be major challenges in dealing with MCH services. Furthermore, the participants indicated that commitment of the system users to ensure complete and accurate data entries is the key factor in achieving validated health data, however, supervision should be strengthened. They indicated that the web-based application enhances equitable use of technology and health information especially in areas with poor infrastructure such as remote areas, and could be easily upgraded, refined, and modified according to evolved new needs.

#### Usefulness

All the participants agreed that the system provides an excellent platform for data entry and documentation of patient data and services provided. It also improves documentation of data and saves patients’ files from loss and duplication of entries. The system also helps in the retrieval of data and its use among different service providers, and this would improve the referral system among different healthcare providers only if it is used at a national level and by all service providers.

#### Acceptability

All participants agreed that there is a great acceptance among the relevant stakeholders at different levels (managers, health providers, users) to the digitalization of all paper-based data entries and reports. Also, the digitalization of medical records is acceptable to the patients as it improves their access to MCH services within primary healthcare centers (PHCs). However, duplication of data entries (in both paper and electronic reporting format) continues to be the main parries toward improving acceptance.

#### Sustainability

Participants from MOH central directorates indicated that sustainability and national expansion are the main challenges. They believed that sustainability is not only due to financial needs but also due to the needed technical support, maintenance, and training. However, they also indicated that there is a great potential for the system’s sustainability since it is a web-based application that makes it less costly and can be widely used in all health facilities especially remote areas.

#### Human rights and gender

The participants agreed that the system is still lagging behind generating disaggregated data that will ensure proper assessment and analysis on the access and utilization of MCH services by special groups and vulnerable populations. They indicated that the current data is only on the essential MCH services and not the expanded SRH package targeting youth, men, and menopausal women. Moreover, the current data is not responsive to special groups with potential protection risks e.g., refugees, unmarried young people, and others.

#### Gaps identified

One of the main identified gaps within the MOH is that there are many separated/segmented electronic data management systems with potential duplication and overlap. However, not all health services are digitized nor connected, and this is a major challenge for continuity of care. Moreover, the MOH currently is unable to own the system and utilize it at a national or district level. Similar to many previous projects, the MoH completely depends on funding opportunities, and it does not consider internal allocation of funds. It is worth noting that these gaps were related to the broader system. However, they also affect the *h*RHR intervention and, thus, they might challenge its scale-up in the future. Other challenges that face the continuity of care include the poor IT/Internet infrastructure in most MCH centers which leads to disruption of the service or accumulation/inaccuracy of data entry, and that not all medical service delivery points are electronically connected (medical doctor, pharmacy, lab and others). Finally, access to reporting is still very limited with a limited number of currently authorized managers.

### II. Health service providers (*h*RHR users)

A total of 37 health providers working in Mafraq governorate from different MCH centers (Primary and Comprehensive) were involved in the project activities. They received theoretical and hands-on/technical training on the newly developed *h*RHR system, and they use it during their daily work to document SRH services.

#### Demographic information of respondents

We had a total of 37 participants in our study. More than half (56.8%) of the respondents were females and 2.7% were males, while 40.5% did not specify their sex. Almost two thirds (64.9%) of the participants were aged less than 40 years, and 35.1% were 40 years or above. Among the 37 respondents, 22 were midwives, 11 nurses, 3 general practitioners (GPs), and 1 administrator. The majority (97.3%) of the participants were full time staff in the pilot centers. Furthermore, 62% of them worked in the pilot centers for more than five years.

#### System usage duration statistics

Out of 31 respondents who answered the question on the already available CPRS system usage, only one respondent had used it for less than 3 months. Almost one third (29%, n = 9) of respondents had used the system for “3 to 6 months”, and over one fifth (22.6%, n = 7) had used it for “6 months to a year”. The rest of the respondents (45.2%, n = 14) had all used the system for one year up to five years and above. As for the usage of the new *h*RHR system, the period ranged from four months to two years for different users in different health facilities. It is worth noting though that more than half of the participants (54.6%) had used it for more than one year.

#### Specific assessment questions

Acceptability: In comparison with paper files, smooth access to patient information, workflow, and relevance, our evaluation showed a significantly high acceptance of the hRHR by health providers (system users). Overall, 73% of users were satisfied with the system, and 78.4% are willing to keep using it. Areas of acceptance are related to convenience (94.6% agree/strongly agree), confidentiality of data (97.3% agree/strongly agree), improved access to patients’ data (97.3% agree/strongly agree), relevance to daily work (89.2% agree/strongly agree). However, 45.9% indicated that the *h*RHR is time consuming.

Usefulness: In terms of quality of data, documentation, accuracy, security of information, and communication among health providers, the evaluation showed that the *h*RHR is highly useful to health providers (81.1%), especially related to documentation (97.3% agree/strongly agree), accuracy (86.5% agree/strongly agree), and easy retrieval of data (89.2% agree/strongly agree) and improves communication among different health providers for continuity of care (81.1% agree/strongly agree).

Sustainability: In terms of synchronization with the main core system, access to training and technical support, and matching similarity with the patients’ paper files, the health providers indicated a high level of potential sustainability especially when it is related to training and technical support needs which can be provided by MOH (easy to learn 83.8% agree/strongly agree, support services 86.5% agree/strongly agree, training 75.5% agree/strongly agree). Also, 86.5% (agree/strongly agree) that the web-based application works in synchronization with the core system used in the MoH, but only 67.6% indicated that it can accommodate new variables/fields with minimum cost and effort. Furthermore, only 56.7% indicated that the system will be able to generate the needed reports and indicators.

Governance/data security: The health providers indicated that the system has clear data access authorization and validation (83.8% agree/strongly agree), a clear guideline for data sharing (73% agree/strongly agree) and 73% indicated that it has a clear guideline for data users.

Feasibility: In terms of simple requirements (ICT, internet), generation of needed reports, and costs, health providers indicated that the *h*RHR can be used in all MoH centers and hospitals (89% agree/strongly agree), however, only 64.8% perceived that it can be used by other health service providers *outside the MOH*. The providers indicated that the system needs supported IT infrastructure, e.g., laptops or desktops, and 35.1% indicated that it is available on any internet browser. Slightly over half of the respondents (51.3%) agree/strongly agree that the new electronic system provides the same reports as those used and generated manually at the central level MoH health facilities, yet more easily and with increased efficiency.

Simplicity: In terms of accessibility, flow of information, and entry fields, most of the health providers indicated that the system is simple (81.1%), however, only (67.5%) indicated the system is easy to be accessed with minimum needed training. Only 43.2% indicated that data entry into the new electronic system is not time consuming.

Human rights: The health providers indicated that the *h*RHR system improves delivery of quality health services (83.8% agree/strongly agree), improves privacy and confidentiality of patients (91.9% agree/strongly agree), is responsive to the needs of special vulnerable groups (78.4% agree/strongly agree), provides an understanding of the barriers to access services (67.5% agree/strongly agree), however, only 62.1% indicated that the system empowers patients to make informed decisions related to their health conditions, and 62.1% believe that the patient has the right to access his information within the system (which is not currently the case).

Gender: Respondents indicated that the system could provide gender specific indicators (67.6% agree/strongly agree) and only about half (51.3% agree/strongly agree) indicated that it improves privacy and confidentiality of patients. Only 37.8% indicated that the new electronic system covers all components of SRH, including Sexually Transmitted Infections/Human Immunodeficiency Virus (STIs/HIV), Sexuality and Reproductive Health (SRH), and Gender-Based Violence (GBV) etc. and only 29.7% indicated that the new electronic system can help to identify gaps in accessibility among different groups (Men, Women, Boys and Girls) (Married/unmarried) (Adolescent/reproductive age/postmenopausal). Also, only few participants indicated that the new electronic system logically and clearly presents associations among the following sociodemographic determinants and access/utilization of services (Education 32.4%, Economic status 24.3%, and Nationality 21.6%).

Moreover, the participants indicated that the new electronic system can be utilized to define special needs of the different vulnerable groups as follows: Refugees, Internally Displaced Persons (IDPs), and immigrants (indicated by 40.5% of respondents), people with disabilities (indicated by 32.4%), marginalized and other vulnerable groups (indicated by 21.6%). It is worth mentioning that 40.5% of participants indicated that they were not sure how to answer questions related to gender issues.

### III. SRH users in MCH centers

#### Demographic information

A total of 855 women participated in the evaluation from 19 MCH centers (11 CHC, 8 PHC). Out of the total, 75.0% of the participating women were Jordanians, 20.8% were Syrians, and 1.7% had other nationalities (e.g., Palestinians, Egyptians). Most of the interviewed women were highly educated (57.1%), while 5.8% had high school education, 1.4% were illiterate, and 19.3% had basic education. Most of the participants were recurrent users of the health facility.

The most commonly used MCH services were childhood vaccination representing 26.2%, followed by family planning (17.2%), antenatal care (4.1%) and postnatal care (1.2%). Almost half (48.5%) of the women received more than one service type. Out of the 855 women, 70.8% (n = 605) were aware of the newly administered data management system while 29.3% (n = 250) were not. Only those who were aware of the new system completed the assessment questions (605 out of 855).

#### Specific assessment questions

Out of the 605 women who were aware of the system, 84.6% agreed or strongly agreed that the new system speeds up the service, and the majority (89.2%) agreed or strongly agreed that the new system improves the confidentiality and privacy of their health information. Furthermore, more than two thirds (76.7%) of the participating women agreed or strongly agreed that the new system decreases the waiting time, while 10.4% disagreed or strongly disagreed. When women were asked about time that health staff spend with them, more than two thirds (77.1%) of the participating women agree or strongly agree that the new system allows health staff to spend more time with the patient.

The majority of the participants (89.7%) agreed or strongly agreed that the new system allows health staff to easily access patient’s information. Most of the participants (86.3%) agreed or strongly agreed that the new system makes them feel confident about how their health information is being collected and used. Only a third (33.1%) of the participating women agree or strongly agree that the new system made their visits to the health facility more daunting or difficult, and more than two thirds (75.4%) of the respondents agree or strongly agree that the new system has improved the relationship between them and their healthcare provider.

Regarding the time that visits take, more than two thirds (79.5%) of the respondents agree or strongly agree that the new system made the visits to the health center faster compared to the paper-based file. Furthermore, most of the respondents (86.8%) agreed or strongly agreed that the new system has made them feel confident that each subsequent visit will be based on their previous health data captured in the system. Finally, more than two thirds (81.8%) agreed or strongly agreed that they would like to have access to their medical files in the new electronic system through a mobile app.

## Discussion

Based on the results and discussions at different levels and with different groups, our study found that the new *h*RHR has a high level of acceptance among stakeholders, health providers and women using MCH services. The evaluation showed that the system improved documentation of data, decreased time and effort of data reporting and retrieval as well as improved access to patient data. As more capacity is built around the use of the system, 45.9% of system users who indicated the *h*RHR as “time-consuming” are expected to decrease. The stakeholders and health providers indicated that the system is easy to use and requires minimum training and supervision. This is due to the fact that the collected information within this system is similar to the information within the paper-based maternal and child health medical files, and the reports generated by the system are the same as the routine paper-based reports by MCH centers. Thus, users of the new system will only need a minimum level of training to be able to use it. Additionally, it is worth noting that out of all the CPRS system users who answered the question on the system usage, only one respondent had used it for less than 3 months, while almost half of the respondents had used it for one year up to five years and above. On the other hand, the new system was used by all of them upon the introduction. This indicates that most of the respondents could provide valid viewpoints on the comparison of the newly introduced system versus the previous one that they had already used.

As part of the field visits conducted to engage with the medical and registration staff to assess the workflow and status of MCH services following the implementation of the innovative *h*RHR system, the feedback that was received was overwhelmingly positive, particularly regarding the smooth information exchange between the pre-existing CPRS and the newly implemented *h*RHR web-based application. The transition from traditional paper files to the *h*RHR electronic system made a significant jump forward in data management, eliminating the need for bulky paper records for patients. The web-based application can be used with basic IT infrastructure and internet connection which would facilitate the expansion and sustainability of the system. The stakeholders and health providers indicated that the system can be easily expanded to all MoH’s MCH clinics within the PHCs, CHCs and hospitals, and a majority indicated that it can be used by other health providers e.g., the military and the private sectors, which would facilitate a coordinated and comprehensive national system for data documentation and reporting. However, the stakeholders and health providers indicated the need to expand the use of the system within the health facility to include other services (other than MCH) and different service providers to ensure smooth referral of cases and continuity of care especially in centers using the web-based version of the CPRS. They also noted the need to review collected data and link it to reporting needs and defining indicators.

Continuity of technical and financial support is the main challenge to ensure the sustainability of this system. However, our respondents indicated a high level of potential sustainability because of the minimum needed investment on the infrastructure as well as related training and technical support which can be provided by MOH at central and peripheral levels with the need to mobilize financial support from internal budget or relevant donors. Women receiving MCH services are more aware of the presence of the system than before, and they indicated that the system improved the continuity of care, confidentiality, and easy retrieval of their health data. In addition, the participating women indicated that the system improved the quality of care provided by increasing the time their health provider spent with them.

Very few studies have been published on the evaluation of SRH electronic systems. The findings of this evaluation are consistent with many previously conducted studies that evaluate the effect of digitalization of SRH services in particular in relation to availability of accurate and timely data for decision making. A study that aimed to propose, implement and evaluate an Obstetric Electronic Health Record and to develop a healthcare indicators dashboard conducted in a public maternity hospital of tertiary reference (high-risk pregnancy) bound to a public Brazilian university, showed that the presentation of results in the graphic form allowed a simple and more direct reading of the most relevant values, being, doubtlessly an added value in the assistance to the management and control of efficiency of the health facilities [16].

In another study an ‘‘evidence map’’ was developed through a systematic search of articles published between 2005 and 2015 about the use of Health information technologies (HITs) to enhance SRH services in Latin America and the Caribbean (LAC) countries. The assessment showed an increased utilization of technology in the field of SRH and an expanded list of various applications and approaches, however, the study showed a weakness in evaluating the effect and impact of the widespread use of HIS [17].

Another study aimed at presenting frameworks and tools to facilitate the development and secure operation of e-Registries for maternal and child health [12]. The study indicated that the benefits of e-registries are yet to be recognized as many countries are implementing health registries in various forms, the majority in transition from paper-based data collection to electronic systems, but very few have e-Registries that can act as an integrating backbone for health information [12].

## Conclusions

The new *h*RHR intervention has shown a high level of acceptance among stakeholders, health providers and women using MCH services. The evaluation showed that the system improved documentation of data, decreased time and effort of data reporting and retrieval as well improved access to patient data. However, the reporting function and availability of timely reports that would replace the paper-based reports still need to be strengthened in addition to a dashboard (at the health directorate level as well central level) to monitor and provide proper supervision and corrective decisions. Our respondents indicated a need to involve IT and MCH technical staff at a central level and at the level of the health directorates to provide direct support to the users in MCH centers. Furthermore, they recommended that proper costing of expansion be conducted, including infrastructure, internet connectivity, training, and technical support in order to see the scalability of this system.

Key recommendations emerged for both optimizing implementation of the new system, and the best way to expand it. Starting with system optimization, a comprehensive review of data and indicators was proposed, aligning with the expanded SRH service package. Enhancement of MCH and SRH-related indicators is essential for informed decision-making. Standardization of procedures and protocols was proposed to ensure consistent service quality. Moreover, coordination within the MoH was seen as crucial for unifying MCH indicators and improving data collection. Strengthening reporting within the new system is needed to enable timely electronic reports, as was the introduction of dashboards for effective monitoring. Finally, the authorization on reporting should be clear, providing secure data management.

Expanding the system requires reviewing and coordinating projects with health data components, aligning with proposed digital systems. It was recommended that a national plan should outline phased implementation, resource allocation, and support/training. For sustainability of the intervention, planning should address financial aspects. Gradual phasing out of the Electronic Health Solutions (EHS) Company/HAKEEM training support was suggested, and a national core team to take over internal training. In the best expansion scenario, the hRHR system was intended to extend to all Mafraq PHCs, which can then be scaled-up to other governorates. Advocacy with other health providers to foster collaboration is also needed in order to ensure a seamless continuity of care. It was also discussed that implementing the expanded phase for six months would allow for a comprehensive re-evaluation. And finally, it was recommended that further expansion to other governorates can be done later on based on the next phase’s evaluation, which will also promote strategic growth.

## Limitations

Finally, this study was limited to the short duration of the actual implementation of the system due to response to COVID 19 and to the number of piloted centers to show impact. However, the overall finding is still indicative of a positive potential for *h*RHR scalability across other governorates of the Kingdom.

## Supplementary Information

Supplementary material 1. Quantitative data gathered from service users/women visiting the MCH clinics – datasheet in Excel format

Supplementary material 2. Quantitative data gathered from services providers (at MCH clinics) through a self-administered questionnaire—datasheet in Excel format

Supplementary material 3. Qualitative data gathered from stakeholders during the first focus group discussion at the central Ministry of Health (MOH)/national level on 2021-OCT-17—text content in Arabic language in Word format

Supplementary material 4. Qualitative data gathered from stakeholders during the second focus group discussion at the governorate level on 2021-OCT-18—text content in Arabic language in Word format

Supplementary material 5. Questionnaire for service users/women visiting the MCH clinics (in English language)

Supplementary material 6. Self-administered questionnaire for service providers at MCH clinics (in English language)

Supplementary material 7. Focus group discussion (FGD) guide for stakeholders (in English language)

Supplementary material 8. Questionnaire for service users/women visiting the MCH clinics (in Arabic language)

Supplementary material 9. Self-administered questionnaire for service providers at MCH clinics (in Arabic language)

Supplementary material 10. Focus group discussion (FGD) guide for stakeholders (in Arabic language)

## Data Availability

The de-identified data and tools that support the findings of this study are available as supplementary files alongside this manuscript.

## Abbreviations

CHC: Comprehensive Health Center
CPRS: Computerized Patient Record System
EMPHNET: Eastern Mediterranean Public Health Network
FGDs: Focus group discussions
GPs: General practitioners
HITs: Health information technologies
*h*RHR: *Harmonized* Reproductive Health Registry
ICT: Information and Communication Technology
IR: Implementation research
MCH: Mother and child health
MOH: Ministry of Health
PHC: Primary Health Center
SRH: Sexual and reproductive health
TSC: Technical Steering Committee
WHO: World Health Organization
