# Supplementary material for: Evaluation of a piloted digital reproductive health registry in Jordan to improve mother and child health
Source: Reprod Health. 2025 May 31;22(Suppl 1):77. doi: 10.1186/s12978-025-01995-2 (PMC12125747; doi:10.1186/s12978-025-01995-2)
Supplement: Supplementary file 3 — Supplementary material 3. Qualitative data gathered from stakeholders during the first focus group discussion at the central Ministry of Health (MOH)/national level on 2021-OCT-17—text content in Arabic language in Word format [file 12978_2025_1995_MOESM3_ESM.docx]

**EMPHENET the Eastern Mediterranean Public Health Network 2021**

**FGD 1/ End-Point Evaluation**

**DATE: 17 October 2021**

**Time: 9:30-11:30**

**Venue: GHD EMPHNET, MEETING ROOM 57**

**Project: إنشاء سجل منظم لصحة الإنجابية لتحسين صحة الأم والطفل**

**Participants:**

| **رقم (1)** | **رقم (2)** | **رقم (3)** | **رقم (4)** | **رقم (5)** | **رقم (6)** | **رقم (7)** |
| --- | --- | --- | --- | --- | --- | --- |
| Manager of FP supply unit/ MoH | Director of Woman and Child Directorate/ MoH | Representative of Administrative support units/ EHS-Hakeem | Representative of Technical support units/ EHS-Hakeem | Representative of Administrative support units/ EHS-Hakeem | Director of IT department/ MoH | Director of Quality and Institutional Development Directorate/ MoH |

**FDG 1 Transcription:**

| المتحدث | المحتوى |
| --- | --- |
| الميسر (Facilitator) | بالاطلاع على النظام من السنة السابقة وأعلم بأن هناك تفاوت بالمعرفة الدقيقة والفنية للنظام شو رأيكم بالنظام وكم بتعتقدو كانطباع عام النظام قادر يستجيب لاحتياجاتنا بتقليل الفجوة الذاتية فيما يتعلق بصحة المرأة والطفل ؟ |
| رقم (2) | النظام ممتاز ليتم تعميمه على المملكة وهذا بعمل سهولة ليتم التواصل مع كل المملكة حتى اعمل مؤشراتي لكن خائفة من الفجوات التي يمكن إن تحدث لقدام هل تستطيع وزارة الصحة حوسبت جميع المراكز حتى نقدر نأخذ المعلومات بوقت واحد مش بس الحوسبة ولكن يجب ُأن يكون هناك انترنت داخل جميع المراكز حتى يستطيع النظام تزويدنا في المعلومات يجب توفر انترنت حتى نعبي هاي الشغلات وهو شيء جيد لتخفيف العبء لنستطيع تركيب انترنت في جميع المراكز |
| الميسر (Facilitator) | هلا رح نحكي بشكل عام على الإطار العام لنظام بعدين رح ندخل بتحديات والخصوصية التقنية له |
| رقم (2) | هلا هو أكيد شيء كويس انه وزارة الصحة بتخفف علينا كثير شغل وعندي بنات يدخلوا كل شهر 700 تقرير على الكمبيوتر حتى نقدر نطبعهم بس أكيد بهمنا هلا يكون في شوي حتى نقدر نعمل مؤشر على وزارة الصحة سريعا يكون في المراكز انترنت هلا في مراكز في المفرق قطعت عنهم الانترنت انقطع الاشتراك ومش عم تقدر تسجل هلا على النظام ، أكيد بهمنا الاستدامة وانه المراكز شوي شوي تتحو سب من حكيم وإضافة الانترنت . |
| الميسر (Facilitator) | يعني يكون هناك خطة واضحة من اجل التوسعة كيف رح تصير |
| رقم (2) | نعم حتى التوسعة تمشي بشكل ممنهج ، في شغله ثانية أي تحديث بددي إياه رح يصير على البرنامج وأي إضافة على التقرير الشهري لأنه عم بيصير عنا مشاريع ومتطلبات جديدة ومؤشرات جديدة لازم نضيفها على النظام |
| الميسر (Facilitator) | رح نحكي مع حكيم أكثر عن موضوع التحديث ومتطلبات التحديث |
| رقم (7) | هلا أنا مع كل شيء الكتروني وربط الكتروني وأي واحد يدخل البيانات من دون استخدام الورق يعني بدل ما الخطأ يكون مرة في الإدخال والي أنكتب في الإدخال بس حد يجي ما يكون في خطأ مرة ثانية أنا مع هذا النظام أنا مع يصير حكيم في كل المملكة ومراكزنا الصحية ووزارة الصحة تكون مربوطة بنظام واحد . |
| الميسر (Facilitator) | عم تطلعي بشكل عام على برامج الجودة وبشكل عام على التطور المؤسسي وكم هاد الكلام عم يغذي إستراتيجية وزارة الصحة ؟ |
| رقم (7) | أكيد عم يغذي و بيساعد صانع القرار بأنه يكون عندي مؤشرات واضحة انه اقدر استند عليها إذا بددي اعمل أي إستراتيجية تكون موجودة بين بددي بعدين شي إلي عم تحكي والهدف إلي عم تحكي وصلني ممتاز بالعكس إحنا مع انه تعمم الفكرة بما انه مربوط فيها حكيم وبما انه حكيم إلي عمله إحنا مع إنها تتعمم في كل المملكة أنا معا ومؤيد اله . |
| الميسر (Facilitator) | من وجهة نظر حكيم يعني شو رأيكم كونكم الشريك الرئيسي بموضوع نظام المعلومات الصحي (Health Information System) مش لوزارة الصحة للقطاع الصحي حاليا كيف نظرتكم لنظام وشو نظرتكم للنظام انه عم يقدر أو رح يقدر يغطي هذه الفجوة المعلوماتية الموجودة واعتقد في مكونات إضافية تم إضافتها نفس حكيم ما زال عنا معلومات ورقية موجودة على الورق في تأخير مابين وصول المعلومة من المركز الصحي لعند المركز تقريبا ثلاثة أشهر, كم تعتقدي هذا النظام انه قادر يستجيب لهذه التحديات الموجودة حاليا وبشكله الحالي ؟ |
| رقم (5) | هلا إيصال المعلومة بشكل الحالي بلبي 100% أصلا هو تم بنائه ليوصل المعلومة بيضل دقة المعلومات له جزئية في جزء منها كليا من النظام وفي جزء منها التزام من المستخدمين في إدخال المعلومات النظام لما يكون في محاور وين الخطأ انه المستخدم يستخدم النظام ويبلش ينبه انه آنت لازم ادخل هون وانه المعلومة ناقصة وانه المعلومة مش ناقصة ولازم تعملها لكن في ناس عم تهجر الملف الالكتروني هجران كامل هذا الكلام يعني انه بقدم الخدمة من برا لبرا وهذا الكلام لازم ننتبه لو عشان نوصل المعلومة بشكل صحيح . |
| الميسر (Facilitator) | بنهاية أحسن نظام أنا بنيته إذا المستخدم نفسه ما كان عنده تقبل له ما رح ينجح |
| رقم (5) | عشان هيك كثير حلو نسمع من المستخدم إذا عندهم تحديات ، إحنا ما سمعنا إذا عندهم تحديات برغم من ذلك كان عنا هفوات كثيرة بهجران النظام كان عنا تذبذب في البيانات إحنا ما سمعنا منهم إذا عندهم مشاكل هل هي تقنية مثل ما تفضلت الدكتورة هديل بالمطلق ولا موضوع انقطاع الخدمة عنهم ، ولا مشاكل تقبل ولا في مشاكل إحنا مش عارفين عنها ، إلا إحنا بدنا نطلب كمزودين للبرنامج انه يكون اتصال مباشر ، يضل في متابعة ، يضل في حد يخبرنا شو عم يصير |
| الميسر (Facilitator) | انتو معنيين بشكل أساسي بنتائج التقييم إلي رح تطلع |
| رقم (5) | شي أكيد 100% هلا النظام تم بنائه بحيث انه يستوعب التحديثات ، أنعمل وأنبنا لأشخاص فقط الأمومة والطفولة ، فهذا النموذج بمعزل عن النماذج الموجودة في حكيم يعني مش مطر انه يكون مرتبط بتحديثات إلي بتحدث على النظام بشكل عام نعمله بشكل منفصل ، بشكل خاص ممكن ينعمل له أي تحديث مطلوب ، ممكن إضافة البيانات ، ممكن تغيير شكل البيانات إلي ممكن يتغير ، وتقارير الممكن تتغير ما في مشكلة لأنه العمل بحد ذاته النموذج تبعه وممكن عزله تماما عن نظام حكيم بدليل إحنا طبقنا في مراكز لا تطبق حكيم . |
| الميسر (Facilitator) | ما تم تدريبهم على حكيم وما بعرفو حكيم |
| رقم (5) | نهائيا وتم تطبيقه في هذه المراكز ، وكنت بدي اعلق على نقطة كنت عم تحكوها عن تبادل البيانات في بعض المراكز انه ما في اتصال بين مقدم الخدمة في مركز الأمومة والطفولة وبين الطبيب، لأنه الطبيب لا يطبق النظام لأنه المركز كله غير محو سب ، إذا بنفكر قاعدين في فجوة بين تبادل البيانات على الملف الطبي نفسه ، واستكمال باقي البيانات للمريض أو المنتفع من خدمات الأمومة والطفولة فنحن عم نحكي عن تطبيق نظام كامل انه يتم حوسبة كل شيء ، يعني نعرف شو عم يأخذ هذا المريض وين عم يروح شو بيوخد أدوية وشو عم ينتفع . |
| الميسر (Facilitator) | سواء داخل المركز الصحي أو خارجه |
| رقم ((5 | هذا بدو تطبيق لنظام كامل ، لكن إذا بنحكي عن ملف الأمومة والطفولة بشكل خاص تحديدي بشكل خاص عمل الأمومة والطفولة فنحنا بنقدر نتوسع بس في هذا الجزئية في كل مكان ، بنقدر نعزلها ونتوسع فيها ونحدثها بمعزل عن كل التفاصيل الموجودة والمعوقات الموجودة في نظام حكيم والكلف الأخرى. |
| رقم (7) | بدي أسال هلا هدول المراكز إلي يستخدموا النظام محوسب مع حكيم هلا هذا المركز رح يصير عم يعبي البيانات من حكيم رح يصير هناك زيادة على النظام |
| رقم (5) | هو جزء من حكيم ويمكن عزله يعني لما يطبق حكيم يدمج . |
| رقم (1) | هلا في شغله مهمة بددي احكيها لأنه قسمي هو متابع قسم الإنجابية بشكل عام وعنا المشاكل إلي نواجهها بهذا الموضوع والأخطاء وتصححيها وال أخره ، ما هي الخطة إلي رح تبرهن أمور الخطة التدريبية يعني إحنا لما عملنا حطينا انه رح يكون هناك تدريب على التوثيق وتدريب للكوادر على أي شيء جديد كيف بدهم يسجل او يعملوا برأيي التوسعة لازم يكون معها برنامج تدريبي واضح وشديد لكل القطاعات والكوادر لجديدة على البرنامج ولا رح نرجع على الأخطاء وتصحيح الأخطاء وهذا رح يكون مشكلة كبيرة للكادر . |
| الميسر (Facilitator) | هل موضوع التدريب رح نرجع نحكي عنه لأنه رح يجي ضمن جزيئات الاستدامة لأنه الاستدامة مش فنية وتقنية وهي كمان الإدارة و إدارة البرنامج بشكل عام والتدريب عليه يمكن هلا رح نحكي عنه أكثر . |
| رقم (4) | هلا 16 المركز الصحي إلي تم تطبيق عليهم النظام كان الهم تدريب ودعم من الألف إلى الياء . |
| الميسر (Facilitator) | والمتابعة التقنية أول بأول ولكن تخوفنا اليوم انه بنحكي عن 700 مركز هلا مراكز الأمومة والطفولة 500 واشي هلا إحنا قادرين نضبط 19 لما يصيروا 500 هلا إحنا رح نفكر شو الدور بين حكيم والوزارة ، لما أتعامل مع تجريبي بقدر اعمل ضبط إلها وبقدر أروح وأجي. |
| رقم (2) | هلا في احد المراكز ومحللي البيانات بيعرفوا هذا الكلام لقينا في خلل في المدخلات لأنه صار في هبوط في الأرقام فجأة اكتشفنا انه قابلة كانت في إجازة أمومة وطفولة وما حد غطى محلها والي غطى محلها ما عرفنا ندربه . |
| الميسر (Facilitator) | بما انه بنطلع على وزارة كاملة وعلى نظام كجزء من مكون كامل كم تعتقدي انه النظام بشكله الحالي والإمكانيات والوظائف إلي يغطيها اليوم قادر يستجيب للحصول على المعلومات بقضايا صحة المرأة والطفل وهي مكون كبير برعاية الصحية الأولية؟ |
| رقم (6) | كنظام هو كثير مهم هلا لازم نبدأ نفكر نعمل نظام شامل لكل موقع ماسكين أنا مش مع انه أضل موقع محو سب وعشرة ورقي أو فاكس لا كبداية شيء كثير ممتاز، إلي خلى الموضوع أسهل انه هذا النظام أنعمل تطبيق فانا عم بحكي هلا ممكن أطبقه على أي مركز غير محو سب هذه جزئية كثير مهمة بتخليني اطلع لقدام انه حتى باقي أجزاء نظام حكيم ، أنا كنت حاكيه الموضوع هذا أكثر من مرة يتحول تطبيق حتى أصير أنا بدي أحوسب الملف الطبي المحو سب ما بدي كل الإجراءات إلي أنا بخدها والاستثمارات عشان أنا أحوسب الملف الطبي بكل موقع وعم بيكون فيه تكاليف باهظة جدا مادام أنا بدأت أحول التطبيق لنقطة بداية كثير مهمة وانه أنا أفكر انطلق ليصير النظام كله تطبيق بيصير النظام تطبيقه كثير أسهل وبأي قطاع كثير أسهل . |
| رقم (2) | بيصير على المطبقين أسهل ولكن كلف استدامة العمل هلا أنتي أول استثمار بتدفعي ارخص ولكن بعدين بتصيري تستديمي أعلى لأنه بدهم infrastructure و collectivityوdata selector. |
| رقم (6) | أنا بطلع على end user كمان أنا بهمني يكون يتعامل مع النظام بسلاسة أكثر، فبيكون هيك نقطة بداية كنير مهمة وانطلاقة كثير مهمة ، احد التحديات حكتها الدكتورة هديل أنا ألان عملت دراسة عن اذا بددي اشبك كل مراكز المملكة بالانترنت و ADSL بسرعة متوسطة يكلف 300سنوي ألف دينار وهذا كان تحدي . |
| الميسر (Facilitator) | هل تعتقدي أنه system cost effective تكلفة النظام فعالة بناءا على إلي هلا بنحكي عنه ، بمرحلته الحالية وبشكله الحالي هل هو cost effective.. |
| رقم (6) | نعم أنا بشوف أنه آه فيه داعي انه نعمل استثمار مفيد بيصير عندي ملف . |
| الميسر (Facilitator) | النظام يكلفني وما بأخذ منه في الأخير رح يهجر. |
| رقم (6) | لازم يكون في متابعة زي ما حكت لمى كان في هناك فجوة في البيانات لو في متابعة بشكل دوري البيانات أدخلت أو ما أدخلت لازم أكون عارف في أكثر من جزئية ، متابعة البيانات تدخل بشكل دائم وكل شي ورقي يكون مدخل الكترونيا ، إذا ما دخل كل البيانات أنا ما بكون حصلت على معلومات دقيقة رح يصير في عندي فجوة بين الورقي والالكتروني . |
| رقم (7) | هلا هي الفكرة مش أنه دخل أو لا هلا بحكي كمان عن تحقق البيانات ، جودت البيانات هلا ما بعرف إذا نظام الأمومة والطفولة أكيد عنهم دخول كمان مع حكيم لو عنا كان طلعنا لما تشوفوا مثل ما حكت لمى أنه في فجوة عند هذا المؤشر بحط مليون علامة استفهام ليش المفروض أنه ندخل عند الجودة أنه بددي أعرف شو هي الأسباب أنه نزل هذا المؤشر سواء كان سلبي أو ايجابي فهذا يسوى أنه مديرية الأمومة والطفولة هلا أنا جديدة بعرف عن النظام . |
| الميسر (Facilitator) | جزء من قعدنا بدنا نعرف مين هو أصحاب المصلحة اليم عنين يكونوا معانا للمرحلة القادمة . |
| رقم (7) | هلا فيما يخص النظام يمكن حكيم بعرفو بما يخص information system في الوزارة المفروض dashboard يكون عندي ، يعني dashboard إلي بروح لصاحب القرار زي الوزير ، هلا المفروض البيانات أحولها لمعلومات حتى استفيد منها وابني عليها استراتيجيات . |
| الميسر (Facilitator) | البيانات أنا كمستخدم كقابلة مثلا قاعدة عم بدخل وعندي معلومات كثيرة وهذا ألحكي كان عبء أصلا على المعلومات إلي بنحطها في الورقي وقدي في عنا كم هائل من البيانات إلي قاعدين عم بنعبيها إحنا قاعدين عم نحكي عن بيانات احترافية قدي هاي البيانات الاحترافية في ثلاث مستويات في الأخر هلا لما أجي بددي أقول القابلة الموجودة عندها رئيس المركز بشكل مباشر بدو يشوف البيانات وشو انعكاساتها الداخلية عنده على المركز في المقابل بدو يكون في ثلاث أربع ل عشرين مراكز بصب في المديرية قدي عنا القدرة اليوم من النظام أنه نشبك المراكز هاي مع المديرية ويكون في عنا قرارات على مستوى لا مركزي ، قدي النظام قادر يعطي أهلية على مستوى المديرية بعدين عندي أنا 14 مديرية في المملكة قدي هدول 14 مديرية في النهاية كلياتهم يتحوسبوا ويصبوا في المرحلة المركزية سواء بمديرية صحة المرأة والطفل أو مديرية الجودة أو كل المديريات المعنيين الموجودين كم بتعتقدو أنه 3 مستويات ويمكن صاروا 4 مستويات قادر النظام اليوم بطريقته اليوم بمعالجته او انتقاله للمعلومات قادر يستجيب إلها . |
| رقم (5) | هلا إحنا في البداية نطاق عمل المشروع لما كانت غير متوفرة ، ما كانوا قادريين يعملوا المتابعة صح كان الموضوع بنتفرج عليه بتواضع انه الفكرة انه نحط البيانات الاحترافية ودايما الإيصال بيكون أسهل طالما البيانات موجودة ، طالما عنك ملف الكتروني موجود ومحفوظ أصغر مستوى من المعلومة الموجودة وإذا لبه موجودة بتبني عليها قد ما بدك ، بيصير الموضوع تجميعي ، ومين المالك مش مهم ووين يتسوى التجميع مش مهم ، هلا بصفي في شوية تحديات إلها دخل بالتكاليف ، والبنية التحتية ما بدنا انظم ونرجع نحكي في الموضوع لأنه الموضوع بالأخير إذا تم بالإجماع على إضافة قيمة بيصفي هذا الموضوع موضوع مشروع يعني موضوع تخطيط ، وميزانية ، ومراحل ، وطالما المعلومة عم تنحط على مستوى صغير هلا الفكرة في الموضوع لما أنطرح الحل لنظام خليني أقول إحنا بنسمي نظام لا نهائي ، لأنه عملنا شي خاص بالأمومة والطفولة ، فبنرجع له كنظام للام والطفل ، الفكرة فيه أنه ما في أي حد مسئول عن جمع البيانات ، في ناس مسئولة عن تقديم الخدمة , حتى ما يصير في عبء على شخص خص نص محدود حتى يجمع البيانات وحتى يجمع الملومات ويرجع إدخالها على نظام تجميع أو يرجع يجمع المعلومات ، هلا هو طالما أجا الطفل على عيادة الطفولة يريد أخذ المعلومات الروتينية تبعته أو أي خدمة من الخدمات الموجودة في العيادة مجرد أنه القابلة أو المسئول يريد التعامل مع الطفل وتأخذ المعلومات تبعته وتأخذ إشاراتها وتحط معلوماته فهي مجرد عم تقدم الخدمة وعم تعمل توثيق للخدمات إلي بتقدمها ما عم تجمع معلومات ما عم تشتغل على أي عبء إضافي إله دخل في أهداف التوعية. |
| الميسر (Facilitator) | بدل ما أعبي على الدفتر بعبي الكتروني هذا المطلوب . |
| رقم (5) | إلي بتعبي الكاونتر أنتي ما بتعبي تجميع ، ما عم بتقولي أجاني اليوم طفل وهذا الطفل بدك تعبي وألام قدامك أنتي عم بتعبي كاونتر ، يعني أنتي عم تتعاملي وعم بتقدمي خدمة وهذا خلال عملك الطبيعي ، خلال الدور الطبيعي تبعه عشان هيك هاد الكلام يتيح إلنا نجمع المعلومات أكثر من حاجة المؤشرات ولكنها مهمة لأنها إلها دخل في الملف الالكتروني ، برجع لتاريخ المرضي ، برجع للملاحظات الطبية ، هذا يتيح إنه أي شخص قادر يحصل على المعلومات يقدر يعمل التجميع إلي يريده ، ويقدر ينربط مع أي نظام بحاجة إله سواء من وزارة الصحة أو من النظام الموجود عنا أو أنظمة تجميعية أو تقارير إحصائية بتنحط للمديريات أي حد يقدر يستفيد منها . |
| الميسر (Facilitator) | يعني البنية التحتية الأساسية |
| رقم (5) | يعني هون الخلال دكتورة المشكلة انه استكمال هاي الجزئية يعني إحنا قد ما حكينا إنه هاد كان الهدف رقم واحد يعني هذا حجر بداية إذا حتى ما حطتي أول درج أنتي لسا عم تعملي حجر البداية عم بتحطي بنية تحتية لنقطة تزويد الخدمة وعم بتقولي عن الخدمة الي لازم تتقدر بعدين بنفكر في التقرير وغيره . |
| الميسر (Facilitator) | أنا أعتقد إنه جماعة حكيم وجماعة IT أكثر الناس إلي كنت باحتكاك مباشر مع end user إلي هي القابلة إلي قاعدة تستخدم حكيم عم تستخدم النظام قدي بتشوف في تقبل حقيقي اليوم من القابلات ومن الأطباء إلي بدو يستخدم إحنا معظم المستخدمين قابلات الآن قدي في تقبل وقدي تقبل من الدعم الفني الموجود وشو أسباب عادتا أنا بعرف أنه في تواصل مباشر معكم أنه في مشكلة أو علقة الشاشة عم بدخل مش عم بحفظ هاي الأشياء إلي كلنا عم نسمعها ، وكانوا كمان من تقيمنا الأولي إلي صار عنا موضوع الوقت إحنا بنعرف الهدف واحدة من الأهداف النظام الإلكتروني إنه في النهاية يحفظ الوقت بدل ما أضيع وقتي ، في جزء كبير تقريبا 40% من المستخدمين قالوا عم بيوخذ منا وقت أكثر من الورقي . |
| رقم (5) | معلش أضيف تعديل على هذا المصطلح يعني الأنظمة الالكترونية لا توفر وقت هي المفروض تعمل على إعادة هندسة الإجراءات بحيث يكون في تحسن في الإجراءات ، ولكن شخص كان في حياته العملية يوثق 20% مما هو كان مطلوب والمفروض كان يعملها يجي النظام كمان يطلب منه يوثق 100% من البيانات أكيد رح يأخذ منه وقت أكثر أو أخذ من وقته زيادة . |
| الميسر (Facilitator) | من علاقتكم المباشرة بالمستخدم أنا بددي أقول بألا خر القابلة إلي قاعدة في المركز وين باعتقادكم ، أول شيء هل في تقبل حسيت من المستخدم لنظام لجديد ولا اعتبروه عبء عليهم والسؤال التالي وين التحديات إلي يلاقوها. إحنا رح نقعد مع 34 مستخدم بشكل فردي مع كل واحد فيهم نأخذ رأيهم لكن أنا بهمني انتم وانتم عم تأخذون كل الأسئلة هاي والتعليقات تبعهم كم انتم بتعتقدو إنه في تقبل من end users من إلي الشاشة قدامه لنظام. |
| رقم (6) | بشكل عام أي نظام جديد عم ينعما وأنا من خبرتي مقاومة التغيير شيء طبيعي في معظم الأنظمة وكثير من الناس كانوا بحكو لك إنه لازم نوقف عند الملاحظات ولازم نشوف الموقع هل هو بطيء أو سريع بدو يكون في أثار ثانية لازم تندرس بالأشياء إلي بحكو فيها ، وبشكل عام المستخدم عنا هو مقاوم لتغيير ، يعني بقولك لازم أكتب على الورقة . |
| الميسر (Facilitator) | أشطب من فوق إلى تحت أكتب ما في لا ، لكن هلا عندي عشرين ميداني بددي أعبيهم. |
| رقم (6) | لازم يكون في إلزامية من الوزارة للكوادر أنه أنا عملت نظام جديد وكان التدريب.ر كبير وما بددي أحدد النظام إنه المستخدم يدبر حاله ، أنا من البداية بددي ألزم المستخدم مش بطريقة صعبه إنه في نظام ولازم تستخدمه لازم في متابعة وإشراف ، أبتعرفي لو ردينا على المستخدم 90% من الأنظمة ما حد طبقها ، إحنا كوزارة لازم نلزم المستخدم أنه كنظام أنعمل نحن بحاجه إله ، أول شغله يكون في إلزامية للمستخدم ، يكون في متابعة لشغل ، أنت كمستخدم أتابع شغلك هل أنت بدخل كل البيانات ، وهل صحة المعلومات بمشي بطريقة صحيحة ويكون في متابعة ، أنا كثير في ناس بحكو أنه هذا النظام معطل لما ننزل على الميدان نشوف ما في مشاكل أنا نزلت لقيت المستخدم شايل الكي بل وبحكي النظام معطل ، أنا بهمني أتابع أي مشكلة بتوصلني . |
| الميسر (Facilitator) | دكتورة هديل قسم المتابعة والإشراف إلي عندك قسم كامل دورهم يشتغلوا على قسم الإشراف والمتابعة كم كانوا فعلا معنيين من بداية المشروع وجزء من الأدوار تغيرت ليكون في جزء من المتابعة و الإشراف على الإدخال الالكتروني . |
| رقم (2) | أي من المراكز الصحية في قسم الإشراف والمتابعة إلي عندي إذا رحن زيارات إشرافية حتى في بقية الأقسام كلهم بنشوف شو نوعية البيانات إلي بتت سجل أذا كانت المراكز محوسبة أو لا ، بنفوت على النظام بنختار أي رقم وطني من عندهم بنفوت عليه . |
| الميسر (Facilitator) | صار في تدريب للمشرفين إلي عندك يعني هل كانوا المشرفات الموجودين الهم جزء من التدريب . |
| رقم (2) | لا هلا أنا بعرف نظام حكيم وبعرف أفوت عليه وكان سهل أنه أفتح وأشوف بس بقية الناس إلي عندي في المديرية ما بعرفوا يفتح . |
| الميسر (Facilitator) | هدول كانوا جزء من الهدف إلي كنا في البداية نستهدفهم للمشروع وما فكرنا فيهم لأنهم الحلقة ما بين الميدان وبين والمديرية . |
| رقم ((5 | في كل اجتماع بيصير برجعولي في النتائج بيكون في شخص جديد بيكون في حلقة ناقصة بيكون في شيء مهم ، مثل قسم الجودة والإشراف والمتابعة . |
| الميسر (Facilitator) | كيف بددي أشرف على شيء أنا معتمد أنا بدي أشرف عليه ؟ |
| رقم (2) | لازم يكون عنده وصول على هذا الحكي قسم المعلومات والتزويد لأنه كل المعلومات بتصب عندهم |
| رقم (7) | هلا أنت بتحكبي بس تروحي على المواقع القابلة إلي بتفتح أنا القابلة عارفة إنه عندي ناس مكتمل الملف تبعه بروح عليهم ، إلي عندكم يكونوا مدربين لأنه إحنا بدنا نعمل معلومات يكون يفتح لأي ملف طبي يختاره عشوائي ونلاقي مكتمل أو غير مكتمل . |
| الميسر (Facilitator) | ويكون عنا سياسة واضحة لموضوع الإشراف ، معناها ممكن خلينا نقول وحدة من التوصيات إنه نعمل تتبع الثغرات إلي بتطلع تدريجيا ما بين end user مقدم الخدمة لغاية ما نوصل الدكتورة هديل ونعمل تتبع تام إلها . |
| رقم (2) | مديريات الشؤون الصحية إلي بدهم يتابعوا برامجهم كمان هدول لازم مدخل. |
| رقم (7) | هلا القابلة إلي بدها تعبي عندها أخطاء بتروح على المديرية والمركز يشيك إذا في خطأ. |
| رقم (1) | انه ما في حد عم يصلح الأخطاء إلي عم بتصير . |
| رقم (6) | لازم يكون في تدخل لمدير الصحة مسئول صحة المرأة والطفل بنفس مديرية الصحة ، هلا إلي بيصير في النظام الالكتروني انه مديرية الصحة بتشيك على البيانات بيروح عندهم . |
| الميسر (Facilitator) | لا تنسي عم يأخذ وقت يعني أنت بتحكي شهر شهرين بس هلا المفروض بكبسة زر أنا أعمل نقدي |
| رقم ((6 | فرصة إنه نشيك على إدخالات المراكز انتو عم يجي كم ورقي بشكل شهري ، هلا الدكتورة ناديا أجاها من مراكز المفرق ورقي التاسعة عشر مركز المحو سب بيوم بددي امسك البيانات وامسك من إلي أجاني من نظام حكيم وأشوف كم في توافق ، لازم البيانات تكون مئة بالمائة مطابقة . |
| رقم (2) | هلا نحنا عم بنزودهم التقارير هناك ، حكيم ما بقدر يعمل تقارير شهرية هلا برنامج هدى تبع المفرق هذا إلي بعمل تقارير شهرية . هلا إحنا طلبنا من حكيم عشان نستغني عن السجلات أول ما أجو على عين الباشا حكينا بدنا نستغني عن السجلات عشان القابلات ما يكتبوا ، حكيم قال ما بقدر أطلع لكم تقارير شهرية إلا إذا طلبتوا منا ، حكيم القديم إلي هلا في المراكز الصحية المحو سبه ما بقدر يطلع تقارير شهرية إلا إذا ضاف تطبيق هدى . |
| الميسر (Facilitator) | أنا ما بوصل لمرحله أقول إني مستغنية عن الأوراق إلا لما يكون في ثقة تامة. لما بنحكي عن قبول وعلى عدت مستويات سواء عن المستخدم أو القابلة أو رئيس المركز أو المديرية معناها لزم استجيب لكل احتياجات لكل المستويات حتى أقول في عندي تقبل للخدمة الموجودة هل موضوع أنه لغاية الآن الأثر ما رح نقدر نحسبه طالما الأثر لسا محدود أنا بحكي عن 19 مركز من ضمن 550 مركز وبالتالي في المرحلة التجريبية ما بقدر أجي أقول انه النظام مفيد إلي وأقدر استبدل أقول حطوا الأوراق والملفات الورقية وخلي إلكتروني إلا لما أوسع أكثر وأكثر وأبدأ أخذ 20% و 50% ، هلا عشان نظمن الاستدامة وهاي وحدة من الإشكاليات إلي بعتقد إشكالية أساسية بأي نظام بتم شغله من خلال التجربة ومن خلال مشروع شو المكونات إلي انتو تعتقدوا أنه لازم نأخذها بعين الاعتبار، إذا بدنا نشتغل على المرحلة القادمة للنظام حتى نظمن الاستمرارية وهون عم بحكي استمرارية ، تقنية ، فنية ، برامج التدريب ، إنه بستجيب حقيقة لاحتياجاتي حتى لو أتغيرت المؤشرات والآلية إلي عم بشتغل فيها ، شو المكونات اليوم إلي بقدر أجمعها كلها حتى أضمن الاستمرارية . |
| رقم (6) | جديد عملنا شروط ل 400 مركز صحي ما في إنترنت ورفعنا حتى نأخذ موافقه عليه ، أنا عم بشتغل عليها حتى نشوف التكاليف وأنا خاطبت حتى أخذ الموافقة واشبك المراكز بالانترنت . |
| الميسر (Facilitator) | وهذا بيساعد مستقبلا في الاستمرارية . |
| رقم (6) | هلا المراكز شاملة والأولية إلي ما فيهم انترنت طلعوا أكثر من 400 مركز عم نشتغل عليهم لأنه بدهم وقت حتى أشبكهم كلهم ، حتى أكمل هذا العدد لازم يكون كوزارة الصحة تكلفة تقديرية لمثل هذه المراكز ، لازم حكيم يقول في كل مركز بدي أضيف النظام هذا إذا بدي أضيف هارد ويرأ وسفت وير بكل التفاصيل لما يكون عندي تكلفة تقريبية كم رح تكلف كل المراكز أنا كوزارة صحة بقدر أحط وأشوف متى بنهي هذا الأمر. |
| الميسر (Facilitator) | أنت عم بدوري على زراعة خطة واضحة من هون لسنتين ل خمسة سنوات معروف فيها مين بدو يشتغل وشو المراحل إلي بدو يشتغل فيها و التكلفة إلها وتكون تكلفة خطة العمل مش حسب ما يتوفر من الميزانية . |
| رقم(2) | هلا بنسبة إلنا يجب توفير كوادر في الوزارة في المراكز الصحية عشان تحل محل وحدة راحت إجازة أمومة ، وحدة راحت تغطي في مركز صحي ثاني ، بدي كوادر كافية في قسم الأمومة وطفولة تغطي أي نقص ، إنه أي وحدة بتروح وبتفضي جهازها وما رح يكون في إدخال معلومات . |
| الميسر (Facilitator) | بدو يكون في خطة واضحة لتدريب الكوادر ونظام إحلال في حال احتجنا . |
| رقم (7) | هل إلي بتحكي فيه الدكتورة هديل يعني شؤون الموظفين يعني أنا إذا بدي أعمل أي شيء وأعمله مؤسسة ويضلوا مستدام لازم كل الناس معنية يعني هي بدها كوادر شؤون الموظفين معنيين في هذا الموضوع . |
| الميسر (Facilitator) | أنا بغير شؤون الموظفين أنا بشيل القابلة من هون وبشيل من هون ما بسأل إذا أنت بتعرفي أو ما بتعرفي. |
| رقم ( 7) | مدير الشؤون الصحية معني ، بدو يجي يكون عارف في المركز القابلة ، مدير المركز مسئول ، الطبيب مسئول ، الأمين العام مسئول. |
| الميسر (Facilitator) | سؤالي كالتالي كم النظام صديق بطريقة أنا مش محتاج برنامج تدريبي ، هل البرنامج بشكله الحالي صديق بالتالي إلي كان يعرف الملف الطبي وحافظ بيقدر يشوفه أنا هاد إلي بعرفه بيقدر يشوفه على الشاشة وبتالي لما أجي أقول بدي كوادر مدربة ، معناها التدريب مش يعني برامج تدريبية طويلة المدى ، قدي تعتقدوا إنه النظام قابل وبسهولة إنه يتم تدريب عليه واستثمار الكوادر إلي أصلا موجودة في الميدان ، بمعنى ما ينزل حكيم يدرب ، ممكن حكيم يدرب استدعاء المدربين call trainers في المديريات والمديريات هي المعنية بشكل مباشر تدرب المراكز ، قدي ممكن من الخطة تكون موجودة ، أنا بقول من ناحية حكيم هل هذا الكلام وارد في خطتكم ووارد بتنسيق مع قسم ITولا لا . |
| رقم (4) | أنا بدي أحكي أنه وفرنا دعم ل ستة مراكز خلال أسبوعين لكل مركز صحي شامل التدريب حتى الناس إلي كانوا مأخذين حكينا مع المديرية ومع المراكز وحتى نغطي المجازين هاد الحكي دورنا وما كان عنا مشكلة ، هلا لما نحكي عن تدريب الناس إلي ما كانوا موجودين هلا وفرنا في كل مركز صحي Super uses. |
| الميسر (Facilitator) | قدي وجود super user انتو قادريين تستثمروا ويمكن إحنا كمشروع نستثمروا في المستقبل بتدريب الكوادر لأنه عنا ضغط عالي. |
| رقم (4) | بوخدوا top training إحنا بنعمل عليهم مراقبة Observing من خلال وجودنا أنهم يدربوا أي حد جديد ، إحنا صار عنا هذا شخص قادر على تدريب أي شخص ويغطي أي مكان وحتى يتواصل معنا سواء الدعم الفني بيعرف يطلب مستخدم ، وإذا في خطوة جديدة كيف يمشي ما نغادر المركز حتى نرفع أسمه للمديرية . |
| الميسر (Facilitator) | هذه الخطوة الأولى إلي بنقدر نظم فيها الاستمرارية لقدام ، هلا أنا في البادية الشمالية شفت في الاستقبال عم يعملوا . |
| رقم (4) | لازم لا يشترط فقط على القابلات . |
| رقم 2) ) | في قسم الإشراف والمتابعة إلي عندي في المديرية لا يقل عن 522 مركز لازم نراقبهم لأنهم عبارة عن شخص واحد ، لازم يكون في فريق كامل للإشراف والمتابعة على مستوى المركز وعلى مستوى المديرية والوزارة . |
| الميسر (Facilitator) | إذا أنا لما بدي أفكر بموضوع الاستمرارية بجانب الدعم التدريبي والتقني إذا لازم أفكر على مستوى محلي وإني أخلقcall trainers' موجودين ومتاحين يقدروا يقدموا الخدمة قدي هذا الكلام كمان قدرنا نفكر فيها في على مستوىIT ، يعني أنا لما حكيت مع الصبية رقية من المديرية قالت لما أنعمل هذا الكلام أنا ما كنت موجودة وما دربت على النظام وبقدر أعمل دعم تقني ، مثال بتقول بتيجيها أسئلة بدل ما أعمل بطاقة وأروح على حكيم وحكيم بعد يوميين يبعتلي شب ممكن يصلح إذا كانت المشكلة محتاجه هيك ، كم في توجه عند حكيم غير موضوع التدريب والعلاقة المباشرة بين مقدمي الخدمة وكمان على مستوى IT الموجودين في المديرية أدربوا على تقديم الدعم التقني والفني بشكل مباشر |
| رقم (5) | بصراحة نحنا دايما أي شيء كفريق أول إلي يقدروا يشيلوا بشيلو بس هاد زي ما حكينا بتصفي بدنا نشرف على البيانات ، ونشكل فريق إشراف على البيانات ، بدنا حد يشرف مع it ويأخذ هذا العبء تبع صلح الطابعة أو أعمل وسوي ، يصير في كوادر يلفوا على المواقع تعمل هذا الكلام . |
| الميسر (Facilitator) | بس مين أسهل إنه أجي من عمان للعقبة أو للكرك عشان موضوع بسيط بينما محليا النقطة المحورية في المديرية قادر إنه يتحرك . |
| رقم (5) | عنا ضابط عادي لدعم الفني في كل مكان بالعكس هدول من الناس إلي يفيدونا. |
| الميسر (Facilitator) | في التقييم الأولي قالوا مع للعلم كل التقييم كان إيجابي ، الدعم الفني والتقني إلي صار وتقدم من حكيم وإنه الأمور بسرعة بتيجي وإنه بتنحل المشكلة على التلفون هاد الكلام بدي أحط بقول في انطباع إيجابي ، لكن بكرا بنحكي عن 19 مركز لأنه بنحكي عن 550 مركز ، بعتقد بدنا نفكر للامركزية ومش بس اللامركزية والملكية من الوزارة. |
| رقم (5) | نحنا ما عنا مشكلة ودايما بنحكيلهم ، إحنا ما بنقدر نحكي بدل إدارة حكيم وإدارة حكيم دايما بتجتمع مع وزارة الصحة لإنشاء لجنة بنسميها لجنة الهمة المركزية ، ويكونوا يقدروا يعملوا هذا الحكي بس ما أعتقد القدرة الحالية لوزارة الصحة ولا بتخيل من هون إلى ثلاث سنوات أنهم تدبر هذا الحمل إلي عليهم ، نحنا بنبني تكاليف وهذه التكاليف للامركزية مستحيل نبعت بطاقة من المفرق لعمان ، فريق المفرق بحيلها ، ها إذا كانت الحالة بتحتاج مادة ، لكن هذا لا يعني أنه يكون في كل مركز إلي بنسميها super user إلي بقدر يحل المشاكل الطيارة. |
| الميسر (Facilitator) | وهذا المفروض نحطوا في خطتنا لما أجي بددي أفكر إنه أنا كوزارة بعد ثلاث سنوات هذا النظام وين بددي أودي هلا أنا بددي أعمل توسع بشكل مفاجئ ل550 المركز ولا بددي أعمل على مستوى اليوم بسكر المحافظة كلها وبكرا بأخذ محافظة من كل إقليم وهكذا بدو يكون الجزء الأساسي من خطة العمل إلي بددي أشتغل عليها. |
| رقم (6) | في مشرف أمومة والطفولة في كل مديرية صحة دورها تابع النظام في عندي IT في كل مديرية صحة هل IT ما بوخد دور الداعم support مش غلط نكون مشاركينه في التفاصيل مش غلط يكون يعرف في الموضوع 3 ساعات مش غلط يخفف عنا شوي . |
| الميسر (Facilitator) | وعلى الأقل على مستوى المديرية هو المركز إلي بدو يعمل الصلاحية . |
| رقم (5) | يعني إحنا بطموحنا وبتشكيل كيف الوزارة بتمارس هاي النظام الكبير ، على ثلاث مستويات على مستوى المركز، وعلى مستوى المديريات هدول المكافئين للمديرية وفي مستوى المركزي في الوزارة ، لازم يكون ممثل أو مشرف بيانات في حالات يتفرج ويشرف على الحالات يتأكد من براعة والدقة ، ومرجعية لنظام كيف شكله كيف يتعاملوا معا . |
| الميسر (Facilitator) | موضوع استمرارية في الخدمة ، يعني وحدة من الأشياء عنا موضوعين مهمين اليوم موضوع استمرارية الخدمة وانتقال الحالة ضمن استمرارية الخدمة ، وثانية موضع referral mechanism قدي نحنا النظام بشكله الحالي ويمكن نحنا بجزء التجريبي وما ربطنا كل المراكز مع بعض ، هل نحن تقريبا المستشفيات كلها محو سبه ، هلا فكرت ل MCH بذات إنه لازم أضمن الدائرة في استمرارية الخدمة ، أنا عم بتابع الحالة رعاية ما قبل الولادة Antenatal care وبتروح بتولد العادي إنه في فجوة مباشرة بصير في قطع كامل بين تسلسل المعلومات وما بين المركز الصحي وما بين المستشفى في المستشفى بولدوها وهم مش عارفين شو صار في antenatal ، بتولد بتمشي على ما بعد الولادة postnatal هون في قطع إنه شو صار في المستشفى ورجوعها على العيادة . |
| رقم (2) | هل إذا كان المركز محوسب والمستشفى محوسب المعلومات بتكون متصلة. |
| الميسر (Facilitator) | كم تعتقدوا إنه هذا النظام بموضوع MCH عم يقدر إنه يستجيب لاستمرارية الخدمة ويضم وزارة الصحة ومع وزارة الصحة و الخدمات يعني إذا بنحكي عن التجربة في المفرق معظم السيدات إلي يعملوا رعاية ما قبل الولادة في الصحة يولدوا في الجيش هلا هذا الكلام قدي تعتقدوا اليوم الوزارة والنظام قادر على إنه يحقق موضوعين استمرارية الخدمة ويكون عندي الدائرة مكتملة والمعلومات كاملة بحيث إني أفتح رعاية ما قبل الولادة وأسكرها family planning المفروض ومقابل الإحالة referral ، هل أنا في المركز الصحي اليوم قادر إنه أعمل إحالة مباشرة للمستشفى والمستشفى قادر يستجيب للإحالة إلي أنا وصلني إلكتروني بحيث كله يكون مكتمل . |
| رقم (5) | هلا إحنا ما بنسمي إحالة لأنه ملفنا متاح للكل ما في داعي أصلا . |
| الميسر (Facilitator) | في نظام في الوزارة لتحويل بفرق كثير ماليا ومنطقي ، إذا طلع تحويل من المركز الأولي أو لا . |
| رقم (5) | صحيح عشان هيك أنا جزأتهم إذا بنحكي قاعدين على استمرارية الخدمة أما إذا بنحكي عن تأمينات وتحليلات وقصص ثاني رح يتبع حوسبة الملف المالي قصته قصة ثانية ، خلينا أركز على استمرارية الخدمة عشان بنحكي شو صار معها في حالة الولادة وشو مخرجات الولادة ووين ولدت وأخذت أدوية بعدين رح تكمل العناية تبعها أو عناية الطفل تبعها هلا موجود في حال عم تولد في مستشفى وزارة الصحة المحو سب . |
| رقم (2) | إذا بدك استمرار في كل المراكز والمستشفيات الخاصة والجيش ، هلا إحنا عارفين إنه إحنا تجربة شو بدنا حتى نتضخم وحتى نتضخم بحيث يستوعب هذه الفكرة . |
| الميسر (Facilitator) | عشان أنا أحقق هدف استمرارية الخدمة يكون في عندي خطة وطنية وليست خطة وزارة صحة يعني لما بنحكي عن خطط وزارة الصحة لا يكفي إلا إذا كان في استجابة وطنية وخطة واضحة وطنية بحيث كل نقطة تزويد الخدمة تكون مشبوكة حتى أضمن الاستمرارية . |
| رقم (6) | هلا لما بنحكي عن وطني ما بنحكي عن وزارة الصحة . |
| الميسر (Facilitator) | اليوم ما رجعنا نحكي عن وزارة الصحة ، إنه هذا التوجه حتى يبين الأثر |
| رقم (5) | ما دام بنحكي عن استمرارية الخدمة على نطاق وزارة الصحة ومحو سب رح يضل في عقبات ، طالمة ما بنحكي عن خطة حتى لو كانت هاي الخطة توزيع الجزئية لصغيرة بس ، هاي الجزئية بتيح يقرأ الملف الطبي لأنها رابطة معنا يعني إذا أنت بدك قرأه وتعرفي شو صار معها بالأخير المساهمة للمركزي في الأمومة بس من خلال هذا السجل ما يقدر يعمل مساهمة خارجية . |
| الميسر (Facilitator) | حتى المؤشرات الوطنية ما رح تنعكس حتى إذا صار الربط هاد . |
| رقم (5) | هو بيقدر يقرأ من ملف رئيسي وبيقدر يكتب عن جزئية الأمومة والطفولة هذا الخط من غير ما يكون محو سب بحكيم بس عم تحكي عن حوسبت الجزئية تبعت الخاص في الانترنت. |
| الميسر (Facilitator) | كم تتوقعوا بس هذا المكون ما بعرف إذا كان عنا طموح حكيم يصير للكل ولكن إذا أنا بحكي كجزئية للويب بأنه طلعنا من الإستراتيجية الوطنية لصحة الإنجابية الايجابية إلي بتطلب أهم بند فيهم موضوع إدارة البيانات يفترض إنه يصب بالاستراتيجي وتكون ضمن الإطار الوطني . |
| رقم (6) | دايما بنحكي التقنية أسهل شيء طريق الربط التقنية قابلة لربط ، أنا بقدر أربط أي نظام بنظام ثاني ، نحن اشتغلنا على تبادل بين حكيم والجيش فترة كثير طويلة وصلنا لطريق إنه ما صار ، هل كتقنية قابلة لربط هلا إحنا من ناحية تقنية ما في أي مشكلة. |
| الميسر (Facilitator) | إذا بمرحلة بناء النظام أخذا بعين الاعتبار إمكانية يكون مربوط كل الجهات بمقدمة الخدمة . |
| رقم (5) | حاولنا ندخل على موضوع البيانات بين الخدمات و الصحة وحكينا إنه هون في جزئية الأمومة والطفولة بس ما طلع بيدنا بصراحة ، سياسي مع زبطت. |
| الميسر (Facilitator) | في عنا تحدي كبير على التفاصيل البيانات إلي هي عنا المتغيرات مثل موضوع الجنسية لأنه كانت كل التقارير السابقة ما بتوخذ في عين الاعتبار الجنسية وخصوصا هلا معظم الجهات المانحة تطلب على الأقل سوري أردني وجنسية أخرى هلا أنا في عندي متغيرات عم تنطلب بشكل أساسي في كل المشاريع بددي أشوف هذا الكلام كم بينعكس على تقاريرنا الأساسية ، التفاصيل بين جنس ذكر أو أنثى والتفاصيل من حيث العمر مش بس أخد فقط الأنثى المتزوج اعمل توسع اقدر اخذ ذكر وأنثى أقدر أخذ أعزب ومتزوج كم النظام أخذ بعين الاعتبار هذه التغيرات . |
| رقم (( 5 | هي متاحة وإحنا أضفناها من دون متطلب إحنا عارفين شو المتطلبات. |
| الميسر (Facilitator) | إحنا اليوم بقدر أقسم حسب المجموعات إلي بددي إياها متزوج أو غير متزوج والجنس والجنسية . هل عكسنا ملف الأمومة والطفولة ما بحكي عن الصحية الإنجابية علما إنه التوجه إني أغطي المعلومات كلها ، كم النظام اليوم عم يغطي مكونات الصحة الإنجابية عم بغطي الرجال والمراهقين و سن اليأس menopause وكم النظام عنده مرونة إنه يستجيب لهذه المكونات لاحقا . |
| رقم (5) | هلا المراهقين و سن اليأس menopause مغطين من حكيم ، هذا النظام MCH إلي عم نتعامل معا حاليا أمومة وطفولة فقط . |
| الميسر (Facilitator) | د.هديل تعتقدي إني بحاجة أطلع من فكرة الأمومة والطفولة لصحة الجنسية والإنجابية. |
| رقم (2) | بحاجة كثير كبيرة . |
| الميسر (Facilitator) | هل ممكن يكون هذا بجزء الثاني لتحديث . |
| رقم (2) | هلا صار جزء أساسي الجنسية والإنجابية ، هلا صار ممكن ندخل سن اليأس menopause ، هلا رح يصير شيء كثير شامل مش موجود عنا في السجلات والمعلومات. |
| الميسر (Facilitator) | إذا بدي أفكر في المرحلة القادمة أطلع من MCH . بشكل عام بمؤشراتنا الوطنية بدينا نفكر أكثر بتغطية الدولية جزء منها الحماية الاجتماعية والوصول للفئات الأكثر تضررا، وموضوع اللاجئين مثلا عم بيأخذ بعين الاعتبار متسلسلة والغير متسلسلة ، لكن كلاجئين في 82 جنسية مسجلة كلاجئين في الأردن كم قدر النظام يأخذها ، غير اللاجئين في موضوع عمال الموسمين إلي بتنقلوا من نهر الأردن من منطقة لمنطقة كم قادرة إني أستجيب إلهم . |
| رقم (2) | فقط بالتطبيع يقدروا يفصلوا ، هلا الجنسية موجودة بس مش على اللاجئين ، فقط في التطعيم بنفرق بين الأردنيين واللاجئين ، اللاجئين السوريين والجنسيات الأخرى ، الباقي عنا ثلاث فئات لتطعيم . |
| رقم ((5 | هلا عم بشتغل مع إدارة المشاريع والتعاون الدولي وحاليا صاروا يدوروا على اللاجئين العراقيين ، الفلسطينيين ، واليمنيين ، صار عنا أكثر من منح بتتقدم للاجئ. |
| الميسر (Facilitator) | الجنسية الأردنية والغير الأردني كم بنقدر نعمل drop list |
| رقم (5) | المشكلة شو تعريفنا للاجئ ، هلا الجنسية بتقدري تأخذيها إنها هاي جنسيته ، هلا تعريفه كا لاجئ هون المشكلة ،إذا صارت أساسية إحنا لما ربطنا مع الرقم الشخصي إلي بتم منحه من وزارة الداخلية فكرنا إنه رح تنحل هذه المشكلة ولكن لا لأنه ما في ما يميزه حتى في وزارة الداخلية الوحيد إلي ممكن يعرفوا إنه رقم المفوضية . |
| الميسر (Facilitator) | وعائلة رح يطلع عندك 10 أشخاص في نفس الرقم. |
| رقم (5) | معلش المهم إنه نعرف نربطه . |
| الميسر (Facilitator) | التقرير كله صار مبني على الجنسية . |
| رقم (6) | أنا في التطعيم كثير يسألونا كم عدد ألاجئين إلي أطعموا ، أنا عندي كل الجنسيات ، أنا عندي نص مليون أردني شخص اطعم لكن كم بدهم لاجئ ما عرفنا ، هلا في عنا الرقم الشخصي ورقم المفوضية ممكن يكون في ربط بينهم في طريقة أو بأخرى الشخص إلي إله رقم شخصي والشخص إلي إله رقم في المفوضية هيك بعرف أنه لاجئ. |
| الميسر (Facilitator) | هذا تحدي مش بس بهذا النظام ولكن بأي نظام نشتغل. |
| رقم (6) | هلا إذا أجتني ست بدها تتعالج أنا ممكن أسألها الجنسية أردنية لاجئ أو غير لاجئ . |
| الميسر (Facilitator) | ممكن أكون سوري بس مش لاجئ ، كل المسجلين على المفوضية أسمه لاجئ .عشان هيك سؤال كان محورين عن الجنسية وبقدر أعملها قائمة ما في مشكلة ولكن ك لاجئ بدها ينشغل عليها . شو الخصائص إلي مش موجودة اليوم وممكن تعطوني إياها كتوصيات عشان نقدر ندخلها في المرحلة القادمة . |
| رقم (2) | بددي اندماج مع النظام تبع صحة المرأة والطفل ، ويكون في تحديث مرن عشان إذا في مكون حديثة بددي أضيفها بكل سهولة ، ويكون في توسع بأقصر فترة ممكنة ، ولازم يكون في دخول بتدريب على مستوى الوزارة والمديريات. |
| الميسر (Facilitator) | هل توسعة برأيك بدو يكون أنا بدأت التجربة في المفرق خليني أعمم التجربة كاملة على المفرق حتى أقدر إنه أحكم عليه ومن ثم مش أقول إني أتوسع . |
| رقم (2) | خلينا في مديرية وحدة ونتوسع بشكل كامل وبعدين بنتقل على المراحل الثانية ، وبعدين بنتوسع وبنشوف المشكلات والعقبات ألي واجهتنا بعدين بنشوف ، تشتيت الجهود في أكثر من منطقة كثير متعب بصراحة ، لما نعملها في مديرية وحدة بتقدري تلفي في المكان . |
| الميسر (Facilitator) | اختيار المفرق كثير مميز لأنهم عندهم تجربة في المشاريع وبيغطي مساحة كبيرة . |
| رقم (7) | أنتي إخترتي المفرق لأنه ما فيها صعوبات مثل عمان مثلا . |
| رقم (1) | يعني أنا لو بددي أشتغل في الشمال وأخذ المفرق بشتغل بنفس الوقت في الجنوب وبأخذ معان المراكز الصحية بطبيعتها وبعدها و صعوبة إدخال الإنترنت في هذه المناطق رح يكون تحدي كبير . |
| رقم (6) | موضوع المفرق بنكمل في المحافظة وإذا بنقدر نوخذ قطاعات غير الصحة إنه أخذ مستشفى خاص ، خليني أكمل المشروع كامل وأشوف شو المعيقات مع القطاعات الأخرى إلي يواجهها. |
| الميسر (Facilitator) | شو المزايا والخصائص إلي ممكن نضيفها للمرحلة القادمة . |
| رقم (5 ) | في شغلي تين ما أخذناهم في عين الاعتبار فكرنا فوق وفكرنا تحت وفي نص لا أنا بصراحة كمقدم ومزود خدمة المديرية ما كانوا معي في الصورة إلا إنهم هم عندهم دخول على الملف ولا كانوا معنا في الرقابية والإشرافية. ثاني شي ما كان مأخوذ بعين الاعتبار إنه كان التركيز إما على الملف الطبي وتركيز على التقارير في جزء بنص وهو السجل إلي هو الكتروني وحسينا فيه لما بلشنا نحتاج بيانات معينة مش تجميعية وحسينا في لما بلشو يحكوا عن الإشراف بددي أعرف أشوف كم ملف مريضة. موضوع السجل لي ما يندرس وينحط في عين الاعتبار وأكثر من مرة حكينا في بعض المشاكل بس ما بتنطرح بس بتنجل بمجهود رجعي ، السجل عبارة عن سجل إلكتروني فيه أسماء ، ولازم يكون إله مالك لأنه ملف حساس في أسماء ومعلومات بشجع إنه نطلع على هذا الموضوع لأنه رح يساعد جدا على المتابعة والإشراف وعلى الرعاية ، ورح يكون مفيد لأي حد لقدام . |
| رقم (2) | هو خارطة الطريق وين أروح و وين أجي . |
| رقم (5) | بخصوص الانترنت ملف حكيم ما بحتاج ملف حكيم بحتاج MBL بحتاج تكون واصلة شبكة الألياف الوطنية ، هاي شبكة أمنة ومعتمدة عنا ، هي أهم من الانترنت ، خذوه بعين الاعتبار إمكانية وصول هاي الشبكة . |
| الميسر (Facilitator) | المشكلة التوجه للمناطق النائية والبعيدة وهاد بمثل تحدي كبير . |
| رقم (5) | في عندك إعتبارت بتقول هذا الملف صفى ملف وطني ، حتى لو في انترنت إحنا ما بنشبك عليه مباشرة إذا شبكة الاتصالات الوطنية مش شابكة . |
| رقم (1) | المهم ما هي الطريقة إلي نتجنب فيها الأخطاء وتبطل توصل للمركز . |
| الميسر (Facilitator) | هي بالأخر data management |
| رقم (1) | ممكن تكون أبسط بكثير يكون عندي قائمة تحقق تبين الغلط وتلقته من أوله ، هلا أنا أول شيء فكرت فيه إنه أحدث البرامج إلي عندي واحدهم خدمات الصحة الإنجابية ، هلا عم بشتغل عليه وعم بغير عليه أشياء جديدة ، مدة 3 شهور رح أطلع بتقرير جديد ورح يتعمم على كل المراكز الصحية ويدربه عليه كتوثيق . |
| الميسر (Facilitator) | بنفس المديرية عندك نظامين قد يتضاربوا مع بعض |
