# Supplementary material for: Evaluation of a piloted digital reproductive health registry in Jordan to improve mother and child health
Source: Reprod Health. 2025 May 31;22(Suppl 1):77. doi: 10.1186/s12978-025-01995-2 (PMC12125747; doi:10.1186/s12978-025-01995-2)
Supplement: Supplementary file 4 — Supplementary material 4. Qualitative data gathered from stakeholders during the second focus group discussion at the governorate level on 2021-OCT-18—text content in Arabic language in Word format [file 12978_2025_1995_MOESM4_ESM.docx]

**EMPHENET the Eastern Mediterranean Public Health Network 2021**

**FGD 2/ End-Point Evaluation**

**DATE: 18 October 2021**

**Time: 9:30-11:30**

**Venue: مديرية صحة المفرق**

**Project: إنشاء سجل منظم لصحة الإنجابية لتحسين صحة الأم والطفل**

**Participants:**

| **رقم (1)** | **رقم (2)** | **رقم (3)** | **رقم (4)** | **رقم (5)** | **رقم (6)** |
| --- | --- | --- | --- | --- | --- |
| Manager of MCH department | Midwife-MCH supervisor | Midwife-FP supply coordinator | Director of Mafraq Health Affairs Directorate Director of Mafraq Health Directorate | Midwife | Head of IT Unit/ Mafraq |

**FDG 2 Transcription:**

| **المتحدث** | **المحتوى** |
| --- | --- |
| الميسر (Facilitator) | باعتباركم معنيين بشكل فعلي للنظام الالكتروني لصحة الأم والطفل بددي أسمع منكم بشكل عام تقيمكم عن النظام وعن المشروع وإجراءات المشروع خلال الثلاث سنوات الماضية . |
| رقم 1 | بالنسبة للمشروع هو سلاح ذو حدين هو يعتمد في النهاية على المستخدم الأخير للنظام إذا كان سهل التعامل معا بيصير الموضوع كثير سهل على مدخل البيانات. |
| الميسر (Facilitator) | هل النظام كبداية لبى حاجة انتم تعتقدوا إنها موجودة . |
| رقم 1 | أنا من خلال زياراتي الإشرافية وإطلاعي على الملف لاحظت إنه نفس الملف الورقي ما بيختلف عن الملف الورقي هو شغلت الكوادر إلي حكينا عنها إحنا مع الضغط هل في مراكز صحية بتشوف في اليوم أو بتغطي بتطعيم 200 طفل يوميا وبتسحب الغدة الدرقية ما بين 250 ل 300 طفل إلي هي مسحة الولادة ، في مراكز صحية عليها ضغط حتى لو إنها مراكز أولية ، زي حي الحسين والحي الجنوبي ، الخالدية . |
| الميسر (Facilitator) | أنت حكيتي أو من كلامك في تقبل ، أنت حكيت شقين من التقبل فكرة إنه المفروض يعكس الورق إلي إحنا متعودين عليه وحافظين لكن الكترونيا يعني ما أجا نظام جديدة وحكومة جديدة مختلفة علي بنستخدمه . |
| رقم 1 | لا هو كمان أسهل |
| الميسر (Facilitator) | أنت تعتقدي في بناءه هو سهل وبقدر امشي عليه . |
| رقم 2 | هو سهل لأنه خفف علي الملفات والبطاقات مكان كله على الجهاز يتم حفظه ، بيعطيك معلومة غلط أو مش غلط ببين على الجهاز أكثر ، يساعدنا على تحديد المواعيد ، نظم الوقت في مواعيد في بيانات ما في عنا مشكلة ، كان في عنا غلبة في الفحص العام لطبيب إذا الطبيب ما وقع كانوا القابلات رح يوقعوا عن الطبيب ، هلا ما فيها القابلات يوقعوا عن الطبيب ، هاي في فجوة في الأطباء ، الفحص العام في فجوة فيها هاي ، ما في فحص عام بيغلبوني ، ليش انه الطبيب ما بيكون فاضي مش مدرب . |
| الميسر (Facilitator) | الطبيب مش بضرورة يكون عنده دخول على النظام . |
| رقم 1 | لا في العشر مراكز الأخيرة بس للأمومة يعني الطبيب العام ما عنده حكيم . |
| الميسر (Facilitator) | المعنى العام إنه في المركز الصحي حتى أحس في الفائدة المفروض كل خطوات تقديم الخدمة تكون مربوطة . |
| رقم 2 | الصيدلية ما فيها نظام حكيم المختبر ما في نظام حكيم بخص أخر مراكز أضفناهم لأمومة والطفولة هاي عملت عنا فجوة. |
| الميسر (Facilitator) | في جزئية عم تتغطى بنظام وفي جزئية كبيرة من الخدمة ما تتغطى . |
| رقم 2 | بيزيد العبء لما يكون عندي مختبر عم ينزل الفحوصات بيكون سهل هلا لا، بدها تروح على المختبر ورقي وترجع دور عنا على حكيم القابلة ، هذه بدها وقت ، جهد ، نفس الشيء الفحص العام لطبيب ونفس الشيء المختبر ، الأشعة عنا فجوة فيها ، كمان عندي فجوة انقطاع الانترنت . |
| الميسر (Facilitator) | انقطاع الانترنت وين المشكلة هل الشبكة ضعيفة ولا آلية ربط الانترنت في المراكز الصحية ضعيفة. |
| رقم 1 | هلا في بطء وانقطاع |
| الميسر (Facilitator) | إنه قدرة الانترنت ضعيفة إضافة إلى الانقطاع وإنه الشبكة ضعيفة . |
| رقم 2 | لكن المهندس حسن متعاون معنا إلى أبعد الحدود ، أي مشكلة بتصير عندكم بس احكي تلفون رح يبين عندي. |
| الميسر (Facilitator) | هل هو بعملكم الدعم الفني من حكيم كامل. أنت عم تحكي إنه التدخل مباشر هل فعلا هذا الكلام بأثر فعليا على تقبل إلي يشتغلوا على النظام يعني ما في إني أجي بكرا أو بعده . |
| رقم 2 | نعم في نفس الوقت إلي بحكي معا المهندس حسن مع الدعم الفني مباشرة بيكون حالين المشكلة ، ما في تراكمات . |
| رقم 1 | على سريع بتفتحي البطاقة يعملوها ربط سريع . |
| الميسر (Facilitator) | آلية الحصول على الدعم التقني والفني آلية سريعة ومناسبة لشغلكم ما بتوقفكم. |
| رقم 1 | بس في الكورونا تغلبنا يعني خلال فترة كورونا 2020كان في مشاكل ما كانت تنحل لأنه ضباط الارتباط لحكيم ما كانوا يطلعوا لأنها كانت مرتبطة بعملية النقل على حسابهم بطلوا يعطوهم بدل مواصلات صارت كل المشاكل تنحل على التلفون ، وأحيانا ما كانت تزبط على التلفون إلى نروح نزور المركز نفسه وما بيصير على التلفون. |
| رقم 2 | الأمور أسهل كيف دكتورة لما أطلع زيارة إشرافية بحكي مع ضابط الارتباط ، طبعا ما هلا قابلة تحكي أنا أنزل ما برد على الحكي هذا ، شو صار عنا نزلوا نظام حكيم على الجهاز تحت عنا شو بنزل شغل عندهم شو فات شغل عندهم خلال الشهر هذا بعكس تقريرهم ، رحت زيارة على مركز وحكيت مع ضابط الارتباط المسئول حكت له أعطيني تقرير شامل على تلفون مطره أحكي معا ، كان سريع الاستجابة ، حكيت معا وحكت له أعطيني شو فات الشهر هذا وتسجل على نظام حكيم سواء نفاس أو حوامل كان سريع الاستجابة وأعطاني أخذت التقارير الشهرية من القابلة والسجلات كاملة حكتلها حكيم عندك صفر قال لا هنادي هي السجل مسجل عليها حكتلها لا حبيبتي أعطيني كم فات لما دخلنا على حكيم كله صفر ، بعدين حكتلي ما سجلت على حكيم سألتها ليش ما عندي وقت ، وفي إهمال هلا زمان تضحك على هنادي إني أنا سجلت هلا ما في تعالي هي سجلتي هيك وعندك حكيم هيك. |
| الميسر (Facilitator) | هلا في الأخر المستخدم القابلة أو الطبيب هو إلي ببين وبحدد نجاح النظام من عدمه ممكن أجيب أنا نظام بمليون دينار واحطوا في المراكز وما ينجح ، كم تعتقد من خلال المديرية ومن خلال قسم الإشراف إنه هذا الكلام ممكن يكون حقيقة قابل لتطبيق وانه يكونوا المشرفات مسؤوليات عن فاعلية إدخال البيانات على أرض الواقع مبارح أنطرح قضية مع الدكتورة هديل إنه قسم الإشراف لازم يكون إله دور إضافي لكل أعبائهم الوظيفية ويتأكدوا ويعملوا تفعيل لإدخال البيانات ما بين الورقي وحكيم وخصوصا المرحلة الأولى إلي فيها جزئيتين . |
| رقم 1 | في عنا مثال في عنا تقرير وهو أعمال الأمومة الشهري هلا كتقرير واحد أعطي لكي مثال هلا إحنا بنجمعوا من كل المراكز الصحية إلي بتقدم خدمات صحة الأمومة والطفل سواء فرعية أولية شاملة عددها 58 مركز عنا إلي بتقدم هذه الخدمات بتيجي عنا بيانات مبدئية هلا المطلوب إندخلها على النظام في عندي هبه المسئولة عن فعاليتها وتدقيقها كامل ، هلا في اختلافات كيف الاختلافات بتبين معها هلا هي بتفعلها وابتعمل إلها تصحيح مع القابلة ترجع تتصل وبندخل كثير بيكون في أخطاء النظام ما بقبلها بنزبطها وبتدخل على النظام . |
| الميسر (Facilitator) | هل النظام بساعدك وبساعد المديرية في موضوع **validation**. |
| رقم 4 | هل زي ما أتفضلت أمل الجواب على الجزئية الأولى بس أنا إلي بدي أحكي موضوع التحديات لازم ننساها ليش لأنه ممكن إنت تحط أمامك أنه لا يمكن حلها أنه النظام لا يمكن يتوقف ، يعني ممكن يكون في نقص بعشر قابلات بتتفاجئي وبيصير عندك أمل ممكن يتم تزويدك في القابلات ، وقبل أسبوعين صارت تنتقل قابلة من معالي الوزير قابلة من عندك على حساب القابلات وبزيد كلف العمل ، والقضية الثانية ذكرتيها إنه صار عندك مش لازم تتكرر في المركز . |
| الميسر (Facilitator) | الحلقة إلي بتربط ما بين التقارير وبين المرفق الصحي |
| رقم 2 | هلا التقارير د.منال تبعت حكيم قعدنا مع الدعم الفني في اختلاف في التقارير أنا حكيت معهم وحكيت إنها ما بتعكس كل شيء في شغل القابلات ، التقرير الشهري إلي بيطلع عندي ، ليش هم بنسبة إلهم كوزارة منظورهم غير عن منظورنا ، أنا بهمني كأمومة يكون في تفصيل حكيت له حكا نحنا بنعمل اجتماع أتواصل مع فاطمة حماد وحكالها في المفرق في فجوة . |
| الميسر (Facilitator) | ليه ما يكون في مستوين من التقارير تقارير على مستوى المديرية وتقارير المركز. |
| رقم 2 | وهاي رح يكون فيها قعده خصوصا على مستوى المطاعيم إنه يعطيني العدد كامل . |
| الميسر (Facilitator) | آنت بهمك تعملي مقارنة وتتبع لكل مركز وتشوفي وين الخلال والمشاكل إلي عنده. أنه تكون جزء من توصياتنا إنه نطلع ونشوف في مستوى التقارير ونشوف في المستويين أنشوف على المستوى المركز ومستوى المديريات والمركز في الأخر بيشوف على 550 وانتووا بتشوفوا من 55 هلا في فرق في الأخر في مستوى المعلومات ألي بددي إياها لكن المفروض النظام يستجيب لحاجاتك وحاجاتهم هذه من ضمن التوصيات الموجودة . هل إذا بددنا نحكي عن موضوع الكادر ، هو بشكل عام بحب يشتغل إلكترونك وبحب يكون عنده نظام ، عم يضل يفكر في التحديات مش ملحق وما عندي وقت ، وانقطع الانترنت ومش ملحقين ، في بعض الأشياء إلي وصلتنا من خلال تقيمنا وزيارتنا الإقليمية ، إنه عدد الناس إلي تم تدريبهم قليل بناءا على إني أقدر أغطي الخدمة وما تنقطع الخدمة في حال صار عنا إيجازه صار عنا غياب وحدة تغطي عن الثانية ، في عنا 38 شخص تم تدريبهم من ضمنها أنتم ، أنتم بتشتغلوا على مستوى أعلى منهم ، وين التحديات إلي أنتم تشوفوها في موضوع التدريب أول شيء هل أنا بددي أدرب بس القابلة ، ولا بددي أدرب كل الكادر الموجود في الأمومة والطفولة ، هل تدريب حقيقة بحاجة إني أعمل برنامج تدريبي وأجيبهم على الاوتيل ، قاعة وأحجز وأفضي لهم يوم كامل ولا ممكن تدريب إذا كان يكون إني أفتح شاشة وأقعد معهم كون إلي حكيتو إلي بعبي معهم بعبي على الشاشة . |
| رقم 2 | فقط يكون معروف ، يعني يطلعوا عليه على الملف هو بعكس الورقي نفسه. |
| الميسر (Facilitator) | هلا عنا نوعين من المراكز ، المراكز إلي عندها حكيم وهدول إلي عندهم خبرة أكثر في التعامل مع النظام والمراكز لسه جديدة على النظام web based ، هل تعتقدوا إني بس رح أستهدف القابلات ولا هل ممكن إني أوسع التدريب على كل المراكز. |
| رقم 1 | كل المراكز ، يعني ضمن التحديات مع نفس الكادر عنا جزء كبير من المراكز مش محو سبه ، بددها قابلة تأخذ إيجازه من دون راتب ، سنوية ، مرضية |
| رقم 4 | هذا بيعني إنه تدريب الكادر أفضل بحيث تنحل هذه المشكلة ، بس ممكن يكون هذا على حساب أشياء ثانية ، لكن المعنى في الدرجة الأولى هي القابلة لكن إذا تدرب الكادر كله أفضل. |
| رقم 1 | قسم السجلات ما درب ، هم إلي يدخلوا ويعطوا البطاقات ، الطبيب العام لازم يكون داري بمعنى التدريب عشان في الفحص العام ، مثلا المختبر . |
| الميسر (Facilitator) | عشان في المستقبل لو وسعت النظام يكونوا مدربين . هلا كان في فكرا يمكن طلعت من التدريب الأولي وهلا نحاول نعززها فكرت يكون في تدريب على مستوى المديريات ، يعني بمعنى حكيم والوزارة أدرب كادر موجود في المديرية هو يكون معني بعملية تدريب للكوادر وحتى الدعم الفني والتقني يكون محلي local أكثر من إنه يكون مركزي central ، كم تعتقد دكتور هاني هذا الكلام cost effective فعال و قابل لتطبيق ورح يضيف على مستوى turn over بقدر أوصل له . |
| رقم 4 | أنا بقارنه في المستشفيات فكرت حكيم بددت في المستشفيات ، حكيم نفسه لما كان في المستشفيات كانوا حكيم هم يدربوا الكوادر لكن فيما بعد أصبحت الكوادر مرتبطة في بعضها. |
| الميسر (Facilitator) | منال أنت بتقولي إنه المهندس حسن مباشرة بيجاوبك يعني لما يصير عندي 550 مركز بددي أتوسع فيه بكل النظام شو بيلحقوه ، لكن في كل مديرية أنا عدي 15 مديرية فيها فريق قاعد عم يدرب ويشتغل بعتقد عملت وصل الدعم الفني والتقني رح يكون أسهل . |
| رقم 2 | شو صار معي كان في مركز نادرة كان عندها مشكلة القابلة بالتوثيق حكت لها اسمعي إحنا موجودين في القسم يا إما تدربي عندي يا إما ببعتك على مركز من مراكز حكيم القديمة من القابلات الكفء تدربي عنها. |
| رقم 4 | يعني أنت أخذتي مبادلة . |
| رقم 2 | أنا بودي كي على رحاب وشوفي شو أقرب مركز إلك ومن البنات الكفء بودي كي تدربي حكت لي ما في مشكلة. |
| رقم 4 | هلا في كل مشروع في البداية صعبة وفي النهاية اعتماد ذاتي، الفريق هذا لازم يكون مدرب على الميداني ، أنت بتكوني بدل حسن. |
| الميسر (Facilitator) | يعني رقية موجودة هون وتقدر تقدم الدعم التقني إذا أحنا احتجنا . |
| رقم 6 | هلا إحنا لما كنا كان في مشكلة في الأطباء كان في أخذ تدويرهم وأخذ إجازات ، لما الطبيب يغطي في مركز كان المركز يوقف عن حكيم ، إحنا بادرنا أي طبيب يتم تعينه جديد ندربه نجمع 5 أو6 أطباء. |
| رقم 2 | بس الأطباء أسهل ، الأطباء عيادتهم بسيطة لكن القابلات لما أوديهم قابلة بقابلة أفضل لأنه عنا أكثر من عيادة. |
| رقم 6 | كطبيب تدريب على CBRS عنده شاشات أكثر من القابلة لكن هي القابلة ألي عندها واحد اثنان ثلاث ل إحدى عشر خدمة موجودة ، الطبيب عنده مساحة أكبر، لما كنا ندرب الطبيب كنا نتغلب كنا نأخذ يوميين في تدريب كانت تشوف على شاشة وأنا عندي هون على الجهاز وندرب ، هلا في شغله أنتو عم تحكوا في هذا الموضوع عشان الوزارة إذا بكم تسلموا للوزارة بدك كادر فعليا يقدم هذا الدعم ، أنت ك IT بدك دعم فني صيانة ، بدك دعم فني تدريب ، وبدك دعم فني تدريب برنامج . |
| الميسر (Facilitator) | أي مشروع يأتي الفكرة فيه والهدف منه كوزارة للنظام ينعمل فترة تجريبه حتى كلنا نتأكد هذا هو إلي بدنا إياه بعدين المفروض يتسلم للوزارة . |
| رقم 6 | أحكي لكي شو الاحتياجات التكنولوجيا بددكم تقدموا لها لكل مديرية تتبنى هذا المشروع بدك أقسام IT يكون فيها شعب مختلفة ، شعب خاصة في الصيانة ، شعب خاصة بالتدريب ، وشعب خاصة في الإدخال ، وهذه الشعب بددها تكون تقدم فعلا دعم ، أنا نفسي بشيك على الانترنت وبعمل صيانة للجهاز ونفسي بعمل صيانة لطابعة ، وبنفس أنا بقدم تدريب وبنفسي أنا إذا كان في خطأ في الإدخال بغير البيانات ، الشغل الواحد يشتغل هذا الكم فعليا هو متشتت . |
| الميسر (Facilitator) | إذا عشان أنا أضمن الاستمرارية ، تلقي وتبني في الوزارة مثل ما هو الهدف لهذا النظام المفروض يكون في خطة واضحة مبنية على مراحل ، مش ضرورة أفكر فجأة وأقول عمم النظام على المملكة بيكون في تدريج في الموضوع وشوي شوي نقدر نبني كل شبكة الدعم إلي عم تحكيها سواء تقني أو فني أو تدريب يكون في تدريج ويكون في مشاركة لمديريات الصحة بكل عملية بناء الخطة هاي مزبوط . |
| رقم 6 | أهم نقطة إعادة هيكلة أقسام تكنولوجيا المعلومات لتخدم كافة هذه الخدمات . |
| رقم 4 | هل أنتم لكم دور مع الوزارة بحيث إنه الوزارة تتبنى بعض هذه الأمور في استمرارية هذا العمل ، الاستجابة من الوزارة لمستموها في إيجابية . |
| الميسر (Facilitator) | بزبط كانت الفكرة في البدء في المشروع من البداية إنه في تقبل من الوزارة ورغبة في تبني النظام هلا أنت عارف صار كل التوجه استبدال الملفات الورقية بالكترونية . |
| رقم 4 | هلا في النهاية بددكم تدخلوا بسرعة هلا في الوزارة في تجاوزات . |
| الميسر (Facilitator) | في مرحلة ثانية في نهاية بدنا نوصل نحنا والممول يقول يا جماعه بيكفي هون سلموا النظام . |
| رقم 4 | إلي يقصدوا هون هل الوزارة عندها استعداد بحيث بعد ما يتركونا إنه خلص . |
| الميسر (Facilitator) | عادة ما بيصير دكتور كم صار في أنظمة في المشاريع الاستمرارية للمشاريع . رقية أنت أعطيتني الوصفة من حيث إنه من جهة IT بددي اضمن الاستمرارية دكتور هاني من الناحية السياسية مثل ما آنت قلت التوجه والتبني من الوزارة كيف المفروض نحن عنا اللجنة التوجيهية ووجود دكتور غازي في اللجنة التوجيهية ووجودك هذا كثير مهم بعكس تبني الوزارة . |
| رقم 4 | الخطوة الأولى التوصيات التي تنبثق على هذا الاجتماع ، وأخذ الآراء بين قوسين استمرارية العملية فيما بعد ، ويكون من عاتق الوزارة الاستمرارية في العمل ، الرجوع للخلف مشكلة ، لازم يكون في دور لو جزئي على الوزارة لضمان الاستمرارية وأهمها تكنولوجيا المعلومات. |
| رقم 1 | وزيارات إشرافية منهم ، لأنه كنا نشوف زيارات إشرافية تبعتنا جهاز الطبيب محطوط على جنب أو مفصول أو ما يستعمله. |
| رقم 2 | الطب العام والإسعاف غير مفعل مثل الأمومة والطفولة ، لو تيجي أنت تقيسي على المفرق أكثر ناس ملتزمين على نظام حكيم الصحة والأمومة. |
| الميسر (Facilitator) | مبارح كان في نقاش مع د.ناديا ود.هديل عن موضوع الإشراف وقالوا نحن المركز ما عنا إدخال على النظام هلا اقترحنا قسم الإشراف في الوزارة مهم يكون جزء من البرنامج صح ، لأنه في النهاية نفرض لو د.نادية أجت زيارة إشرافية إلى المفرق أنا بهمني إنها مطلعة وفاهمة شو عم بيصير ، لأنها في النهاية التقارير رح تصب عندها ، في ثلاث مستويات للفعالية على مستوى المديرية وعندك المركز مديرية صحة المرأة وكانت معنا مبارح د. رندا وهي مسئولة عن الجودة وعن التقرير الإحصائي بتقول أنا مثل لازم يكون في عندي إدخال أنا كمان بهمني ، التقييم بدء يعطي أكثر مين الناس المعنيين بشكل الحقيقي في تبني هذا المشروع ويمكن د. هاني بعد ما نطلع النتائج هذي ما نكتفي في اللجنة التوجيهية ممكن يكون في تصور نقعد مع معالي الوزير وشركاء WHO مثلا مثل USAD أنا بعرف هلا كم عم يطلع برامج اليوم ، كم مهم نقعدهم كلهم ويكون في تبني وما أنضارب على بعض ونكمل بعض أنا وصلت لهون أنت كملني بهذا المشروع مثل أنا بحكي أنا بعمل هذا النظام مثل الاسباني بقول أنا بجيب IT الاتصال إذا ما ارتبطوا الاثنين مع بعض في النهاية أنا كوزارة ما رح أستفيد . |
| رقم 4 | لكن الحلقة الأولى إنه الجهاز الإشرافي الموجود في المديرية يوازي في central جهاز ثاني هذول يكونوا على أهبت الاستعداد بحيث حتى لو صار تقصير من أي جهة ثاني إنهم يمشوا العملية . |
| الميسر (Facilitator) | من الضرورة في المرحلة الثانية إنه نوسع شبكة الناس إلي إلهم تفويض authorization على النظام ومدربين عليه . |
| رقم 1 | التنسيق بين المديريات المركزية السابقة سبق وحكينا عن هذا الموضوع. |
| الميسر (Facilitator) | المديرية نفسها هلا يمكن انتم كثير بتقعدوا وبتحكوا مع بعض ، إحنا اكتشفنا في الصدفة د.نادية بتقول عن نظام اليوم بنبنى مع USAD بصدفة عن متغيرات الصحة الإنجابية ، بضرورة يتقاطع مع النظام تبعنا ، بصدفة هذا ألحكي ، وحدة من أهداف النظام غير إني أعمل إدخال بيانات ، موضوع استمرارية الخدمات ، في المراكز الصحية في توجه للاعتمادية ، وأول شرط في الاعتمادية وجود ملف واحد فقط للمريض ، سؤال كم بتعتقدوا ك ضمان للاستمرارية إحنا حقيقة ك نظام قدر يعمل رعاية مجتمعية ما بين داخلي internally وزارة الصحة أو مع القطاعات الثانية خلينا نبدأ نحكي عن وزارة الصحة بعرف في تحديات كثير مع القطاع الأخر ، بوزارة الصحة هل بتعتقدوا إنه النظام قادر إنه يحقق أنا طبيب أو قابلة إلي بشوف الحالة ورجعت لي بعد الولادة postnatalفعلا بتحسوا أنه الدائرة سكرت أو لا. |
| رقم 4 | إذا كان على مستوى وزارة الصحة نعم . |
| الميسر (Facilitator) | إذا محو سب إذا محو سب وداخل معي على البرنامج . |
| رقم 4 | نعم ما بين المركز والمستشفى بس ما يكون الحلقة الضايعه في القطاعات الأخرى. |
| الميسر (Facilitator) | لكن النظام قادر في شكله الحالي إنه يربط لي ويعمل الدائرة بشكل متكامل. |
| رقم 2 | كمستشفى حكومي وكمستشفى نسائية كله نازل عنا على الجاهز وعلى الملف . |
| لرقم 4 | وحتى لو في محافظة ثانية. |
| الميسر (Facilitator) | التحدي بيصير في القطاعات الأخرى ، موضوع المرجع ، هلا في عنا مرجع الكتروني وفي تعليمات عن موضوع التحويل بعرف أنا إنه في ناس بتروح على المراكز الصحية بس عشان يأخذوا ورقة التحويل ويطلعوا فيها إلى المستشفى إنه بساعدهم كثير شو بدك تدفع وكم بدك تدفع ، هل النظام بيساعدكم بموضوع المرجع ، هلا برأيكم هل فجوة لازم ينشغل عليها لأنه أنا في النهاية المفروض أكتب التقرير للمستشفى وهذا التقرير . |
| رقم 2 | أبدا |
| رقم 1- | موضوع المرجع كملاحظة ، لحد ألان أي مرجع بطلع ورقي لازم يطلع ورقي مع المريض ، في كمان أشياء كثير. |
| الميسر (Facilitator) | أعطيني شو الخصائص والأشياء نحن اليوم ما عم نغطيها. |
| رقم 1- | الوصفات الطبية وخاصة العقاقير الخطرة والأشياء الثانية ، مثلا وصفات الأمومة نفسها ما إبنستغني عنها كورق ، سمعت في تغير للوصفات وشكل الوصفة والبند شغالة عليه الوزارة . |
| الميسر (Facilitator) | آه في نظام بشتغل هلا على الوصفات . |
| رقم 1- | لو الوصفة تصير تفوت على النظام ، وتتعبا الكترونيا . |
| الميسر (Facilitator) | وهذا بساعد الرعاية المجتمعية على إني أعرف شو الأدوية إلي أخذتها ومن وين أخذتها. |
| رقم 1- | ليش إحنا اليوم إحنا النظام عنا كيف عيادات في عنا عيادة حوامل وعيادة نفاس وعيادة طفل وتنظيم الأسرى لما تيجي المريضة بدها تراجع أكثر من عيادة لازم كل مرة تروح على السجل تأخذ البطاقة هذي مشكلة. |
| الميسر (Facilitator) | هذي أعتقد أنه أول مرة بسمعها ، يعني في فصل في الخدمات داخل النظام . |
| رقم 2- | أنا القابلة تحكي لي بتفوت تنظيم أسرى ونفاس هلا إذا قطعت نفاس بدها ترجع مرة ثانية على السجل تأخذ ورقة ثانية وترجع وتروح على الفحص العام وترجع مرة ثانية على السجل . |
| الميسر (Facilitator) | أمل انتووا تتعاملوا كثير في المراكز الصحية ، السيدات إلي في الأخر عم يشوفوا النظام ، هل السيدات إلي عم نعمل معهم تقيم بشكل عام انتبهوا ، في 60% بس إلي انتبهوا إنه في نظام على فكرة المعظم ما انتبه إنه في فرق هل تعتقدي في تقبل من السيدات للنظام الكتروني ولا في تخوف منه شو ردت فعل السيدات. |
| رقم 5- | أنا بنسبة إلي شفت السيدات حابين الكتروني أكثر من الورقي لأنه مثلا تيجي مضيعة كرتها بتت غلبي تلاقيه تحكي لكي حطي على الكمبيوتر بتلاقيه . |
| الميسر (Facilitator) | واعية إنه النظام عم يساعدهم . |
| رقم 5- | بيجو على مركز ما في نظام يقولوا ليه ما عندكم كومبيوتر كل شي موثق عليه ليش كل الورق ضايع ، الناس حابه الكتروني أكثر من الورقي. |
| الميسر (Facilitator) | عندهم وعي عام بشكل عام إنه معلوماتي كلها موجودة . |
| رقم5- | وحدة من السيدات بددها كرت مطعوم لابنها إحنا مركز ورقي بحكي لها أعطيني تاريخ ميلاده وشوي وبطلع لكي إياه بتقولي حطي رقمه الوطني على الكمبيوتر بيعطيكي إياه . |
| الميسر (Facilitator) | تعتقدي التقبل من المرضى أكثر من مقدمي الخدمات . |
| رقم 2- | حسب سرعة مقدم الخدمة هلا في مقدمات خدمة بطيئات والأهالي يعترضوا إنا مستعجلين وفي سيارة برا وفي أماكن ثانية ماشية منيح. |
| الميسر (Facilitator) | لكن الناس بشكل عام هلا زمان كان في مقاومة وكانت الناس تخاف ليه بدك تسجلني ، تعتقدا الكلام صار في العكس هم إلي يطلبوا يكون الكتروني. |
| رقم 1 | بددي أحكي ليه بطلوا يجو لأنه زمان كان زمان أيام كورونا بدك تعمل فحص pcr كان في عزوف ويبطلوا يجو على الخدمة يطلبوها والخطوة الثانية لسيدات كنا كثير كنا نشوفها في الزيارات الإشرافية إنه بددها كل مرة تفوت على السجل فكان في انقطاع عن الخدمة أو ما بددها تأخذها . |
| الميسر (Facilitator) | فبتالي لازم نأخذ في عين الاعتبار نشتغل على مجموعة ونقدر نحول الداخلي ضمن المجموعة هذه مش كل مرة نفتح سجل. |
| رقم 1 | وياريت مثلا المستشفيات الجامعية في إلها مثلا أشياء أحلى مثلا في الحجز الكتروني ليه ما يكون في حجز الكتروني. |
| الميسر (Facilitator) | أنت حاكيتيها كم أنا اليوم كنظام ، وحدة من الأحداث إلي كنا حاط ينه المريضة نفسها يكون فيها نوع من التحكم والدخول على ملفها الطبي بحيث لو طلع لي فحوصات معينا بدل ما أجي على المستشفى عشان بس أخذ ورقة الفحوصات أنا لو عندي رقمي الوطني بقدر أطلعوا ، مثل القطاع الخاص ، هل تعتقدي السيدات وخاصة المواعيد أو الاطلاع على نتيجة المختبر بدل ما أجي . |
| رقم 6 | حكيم بلش في هاي الخدمة مش عشان الحجز عشان مشاهدة . |
| الميسر (Facilitator) | مشاهدة ويكون عندي إدخال محاولة مشاهدة. |
| رقم 1 | تذكرني في الموعد تبعي في كثير ناس بتنسى ، في ناس بتيجي تطلب الخدمة اليوم بدل الخارطة الصحية كثير في ناس ألتزمت فيه وفعلته صح ، أنت كمركز شامل عنك خدمات بتوفر طب أسرى ليه أنا أجي الأحد وما عندي طب أسرى ، ليه ما أجي أنا على موعدي عند الطبيب إلي بددي إياه. |
| الميسر (Facilitator) | يعني بتعتقدي إنه النظام يبدأ يفكر في طريقة إنه المريضة تقدر تدخل على ملفها الخاص من خلال الرقم الوطني ويكون عندها خاصية المشاهدة ، إنه أنا في أشياء بقدر أخذ موعد ، بقدر أعرف شو المواعيد المتوفرة لخدمة معينة هذا المركز مثلا فيه تركيب لولب أو ما في تركيب لولب ، دكتورة بتيجي الأربعاء أو الثلاثاء . |
| رقم 6 | بدي أحكي عن الخارطة الصحية بدنا نرجع نحكي عن التنسيق بين المديريات المركزية هلا أجت منظمة اشتغلت الخارطة الصحية وسحبت حالها وراحت هلا مبدأ عمل الخارطة الصحية خاطئ لانه كتالي لازم ينربط في حكيم أنا كمواطن بددي أدخل على الرقم الوطني وأشوف المراكز الفعالة مش أفتح الخارطة وأعمل تسجيل و ايميل . |
| رقم 1 | هو بس التسجيل لأول مرة صار في مئة تطبيق نحنا بس بدنا نوحد . |
| رقم 6 | الأصح والمفروض إنه المنظمة اشتركت مع حكيم وتنر بط معهم مع الرقم الوطني ومكان السكن. |
| رقم 1 | مع إنه حكيم هو إلي درب على الخارطة الصحية . |
| الميسر (Facilitator) | بددي أرجع على مواضيع التقارير وإنه أنا بالأخر مدخل بيانات بددها تنعكس بالتقارير والمؤشرات عشان أخذ القرار د. هاني على مستوى المديرية كم تعتقد إنه النظام اليوم عم يساعدك تأخذ هذه الفكرة والتحليل ووضع خدمات الأمومة والطفولة الموجود بمحافظتك وكم بساعدك إنك تعكس المؤشرات وتعمل تحليل إلها . |
| رقم 4 | إذا كان الوضع على ما هو عليه ألان ولا إذا تجاوزنا الأشياء إلي عم نحكيها. |
| الميسر (Facilitator) | أنا بحكي اليوم كم النظام قادر على إنه يلبي احتياجاتك والمؤشرات وتقدر تعمل تحليل وتتبع وتأخذ قرار. |
| رقم 4 | أنا بقول النصف يعني 50% لأنه إلي ذكروهم الشغلات المفروض ألان تكون متبعة ألان من هاي الفترة من عمر البرنامج ومطبقه لكن ممكن نحكي إنه الكورونا أثرت حتى لو IT في الوزارة لو ما بدنا نحكي مهم ألان مش فاضين مشغولين بسبب الكورونا . |
| الميسر (Facilitator) | أنت دكتور ماذا تريد من النظام هل أنت بهمك يكون عندك لوحة تحكم ، تتبع ومؤشرات . |
| رقم 4 | أنا المهم الشاشة تكون جنبي وكل شيء موجود وكل شي بددي إياه بقدر أحصل عليه وأنا أعتقد مش صعب يعني أنا الآن مثل ما حكت أمل المستشفيات التعليمية طيب ما هي نفس الأنظمة لكن أي شيء عندهم سهل ، المشكلة أراء مختلفة مش بس إحنا وأنتم من الوزارة وحتى في الاجتماعات لو كان في حد من الوزارة ما رح يكون مثمر ، يعني نتناقش في أمور ما نوصل لنتيجة لكن بس إلي ذكروهم الملاحظات هذه على أرض الواقع ممكن سهل كثير ، أنا الان عندي 90 مركز و 4 مستشفيات حكومية إنسي باقي القطاعات لازم يكون عندي كل شيء أعرفه . |
| رقم 6 | قرار بسيط ممكن يرنه عليه مثلا عنا أزمة في المركز الفلاني بإمكانهم بكبسة زر ، يعني نعرف هل الطبيب يزودها ولا كل إلي شايفينهم 20 واحد . |
| رقم 2 | إحنا شغالين عليه يعني قابلة تحكي لي عندي ضغط بتفتح على الجهاز ونسألها و وين الضغط ، ريحنا كثير ، أو تحكي بددي كادر ، تحكي لي هنادي أنا مش ملاحقه شغل بددي كادر ، بحكي لها تعالي هي أنت مسجلة عندي هيك على أي أساس بدك كادر . |
| رقم 1 | في في الوزارة قرارات جديدة إنه رايحين نربط الحوافز بحكيم ، رايحين نربط الحوافز في الكاونتر مع الأطباء ، أنا بددي القرارات إلي بتطلع تأخذ بجدية وتحبب الواحد بشغله . |
| الميسر (Facilitator) | يعني إحنا دائما بنفكر في الحوافز ، يعني ما في تبني داخلي إني بددي أحسن شغلي. |
| رقم 2 | كل الشغل هيك هلا يحسوه شغل زيادة وأنا حفزيني ماديا بشتغل لكي . |
| رقم 4 | نظام الحوافز إذا طبقوا صحيح بتت عدل الأمور . |
| رقم 1 | هم هيك يطلعوا، والتقارير السنوية فيما يخص التوثيق الالكتروني في شغلات ، بس حكيم ما ينجح إلا إذا كانت كل المملكة حكيم . |
| الميسر (Facilitator) | يعني هذا النظام في مرحلة التجربة لسه ما أخذت العينة كاملة ، الأثر الحقيقي ما ببين إلا لما يكون الكل مربوط وعندي مؤشر واحد . |
| رقم 4 | بددي اسأل الاخوات السؤال إلي سألته د.منال لما قلت 50% هل مبالغ فيه الرقم ولا قليل ولا صحيح . |
| الميسر (Facilitator) | عم بيغطي إحتياجاتنا الحقيقة من تتبع. |
| رقم 6 | أنا بنسبة إلي IT لا كثير أقل صدقا لأنه لما جيتوا دربتوا في المراكز ما نأخذ وجهة نظرنا و وين الخطأ في البرنامج مقارنة في الملف الورقي مرات عم نلاقي خطأ مثل إلي عم تحكي لي إياه في بداية حكيم ما كان موجود خدمات منفصلة discrete services لكن مع التفعيل لجديد والنظام لجديد. |
| الميسر (Facilitator) | إنتوا لو إنكم مش مستخدمين واديكم في الشغل ما حد حكاها ، أنا ما سمعتها وهاي أول مرة بسمعها. |
| رقم 3 | المراكز لقديمة ما كانت كانت مجرد ما تدخل على السجلات بدخلها على كامل العيادات وبشتغل فيها . |
| الميسر (Facilitator) | نحنا عم نحكي عن الصحة الجنسية الإنجابية مقابل الأمومة والطفولة هلا الوزارة بددها تطلع من فكرت مراكز الأمومة والطفولة لصحة الجنسية الإنجابية هذا التوجه لجديد التزام من الحكومة أيضا في التزامات الدولية ، كم البيانات إلي أنا اليوم عم بغطيها عم تعكس مكونات الخدمة أنا عندي خدمات عم بقدمها غير موجودة على النظام ، في عندي فئات عمرية وفئات عمل أنا عم بشتغل معهم وما عم بدخلهم . |
| رقم 2 | في أشياء داخلة ومش مدربين عليها والعكس . |
| الميسر (Facilitator) | مثال menopause سن اليأس عند المرأة. |
| رقم 1 | لا. |
| الميسر (Facilitator) | اليافعين صار في برنامج مع وزارة الصحة عن صحة اليافعين . |
| رقم 2 | العنف . |
| الميسر (Facilitator) | العنف كلكم تدر بتوا عليه وما إله مكان. الرجال هل أنا بقدر أدخل الرجال والخدمات المقدمة لرجال . |
| رقم 5 | على ملف التدخين مش موجودة مع إنها موجودة ورقي . |
| الميسر (Facilitator) | هل إحنا في المرحلة الثانية بددنا نرجع ندور على الملف ، الملف الأبيض إله عشرين ثلاثين سنة ما تغيير ، كم تعتقد اليوم بددي أطلع من الأمومة والطفولة واصلا البرنامج هو الصحة الجنسية والإنجابية ، هل أنا اليوم بددي أوسع الحقول إلي بددي أشتغل فيها حتى تقدر تعطيني مؤشرات متكاملة . |
| رقم 4 | يعني بتحكي عن ملف إله ثلاثين سنة ما صار عليه أي تغير هلا ممكن إنه يعكس المتطلبات الحقيقية في الوقت الحاضر. |
| الميسر (Facilitator) | عندي مؤشرات عم ينعمل عليها تقارير مش موجودة عندي |
| رقم 2 | كان في تعديل على الملفات وكانوا تحت الطباعة بس فترة كورونا توقف كل شيء . |
| الميسر (Facilitator) | بس لما نعمل البرمجة أنعمل على القديم. |
| رقم 1 | هلا في ملفات جديدة وفي نقاط جديدة ، بددنا نعمل تعديل كلياتها علي موجود حاليا. |
| الميسر (Facilitator) | برامج موجودة دخلت فيها وزارة الصحة ما كانت موجودة ، أنا اليوم عندي حقول وخصائص النظام اليوم ما بستجيب لحاجاتي إلها . |
| رقم 1 | إذا كان في تعديل أو شي من الوزارة بصير. |
| الميسر (Facilitator) | لو بدنا ننطلق للمرحلة القادمة حتى أقدر أستجيب لاحتياجاتهم والتقارير وبناء سياسات في الأخر لازم يتعدل ويدخل بعض الحقول الإضافية إلي بتغطي كل خدماتي لأنه الست لما تيجي تعمل فحص السرطان وين بتحطوها. |
| رقم 2 | على الملاحظات . |
| الميسر (Facilitator) | ما عندي تقرير يقول إلي كم ست أجت. |
| رقم 1 | اكتبيها في الملاحظات . |
| الميسر (Facilitator) | بس على فكرة كل شيء تأخذيه ما بطلع في الملاحظات . |
| رقم 1 | في التقرير لا. |
| الميسر (Facilitator) | أنت بدخلي في إدخال البيانات بس في الأخير ما بطلع عندك مؤشرات ولا تقرير. |
| رقم 3 | حتى مشورة فحص الحمل للسيدات طويلة الأمد لما تكون تستخدمها وتوقفها نقول لها زوديها فولك اسد هذا تقرير عنا بس لما يزودها ما ببين. |
| الميسر (Facilitator) | إذا أنا اليوم النظام في بعض الحقول والمؤشرات إلي بغطيها في تقاريري الشهرية الدورية ما بتنعكس على النظام ، معناها بدو تعديل . |
| رقم 1 | في أشياء بتنربط خليها أشياء تشخيصية ياريت مثلا يصير في مثلا تحري فحص الدم عنا هذا مؤشر سواء الأطفال أو الحوامل ياريت ينربط الجزئية تبعته المختبر مع الطبيب العام للجرعة ، مثل المستشفيات ما يقدر يطلع منها الطبيب غير لما يعبيها ، هلا في أطفال كثير بطلع عندهم أقل من 11 . |
| رقم 6 | كانت موجودة قديما . |
| رقم 1 | 10 او 9 بعتبر عنده فقر دم الطبيب بحكي لكي لا هذا مش فقر دم ، متى اربطه ويطلع المريض يؤخذ الجرعة الصحيحة |
| رقم 6 | أجاوبك عن حكيم حكيم قالوا إحنا المتوسط أخذنا منكم أنتو يا وزارة . |
| الميسر (Facilitator) | بناءا على دليل إجرائي ، مطلوب من الوزارة إنها تعدله الدنيا تغيرت من 2011 لليوم. |
| رقم 1 | كل الأدلة هاي أنا وزعت نسخة منها على القابلة والطبيب ، في تناقض. |
| رقم 4 | لأنه الأطباء ناقص عليهم التدريب. |
| الميسر (Facilitator) | في فجوة في الأطباء لكن القابلات عندهم التمكن من التدريب وسهل يتحركوا أما الأطباء، أما يطلع طبيب وتسكر العيادة وتودي |
| رقم 4 | غيرنا ثلاث أطباء من ثلاث أماكن النقص بدك أنت تمشي الشغل العام على حساب بعض الأمور كله بنحل في التدريب المستمر . |
| الميسر (Facilitator) | بددي أسأل عن البيانات إلي بنغطيها كبيانات شخصية للحالة ، الكل اليوم وبما إنه كل المشاريع emergency أجت نتيجة استجابة الأزمة السورية ، كل المشاريع إلي ينفذوها في المفرق لأنه فيها سوريين ، كم تستجيب البيانات على هذا الكلام ، في يأخذ العمر ، الجنس ، الجنسية النظام . |
| رقم 1 | ماشي على معايير الاعتماد هلا معايير الاعتماد أشياء خاصة موجودة لازم مكان السكن ، الحالة الاجتماعية ، ورقم الهاتف هاي الأشياء بتكون في شغل السجل . |
| الميسر (Facilitator) | الجنسية لما تنزل بتكتبوا أردني غير أردني ولا كيف. |
| رقم 2 | بدخل آه |
| رقم 1 | بس برقم متسلسل آه |
| الميسر (Facilitator) | إلي أنا بددي أقوله هل أنا بقدر أطلع عدد السوريين إلي أخذوا هذه الخدمة كمثال من التقرير هلا بقدر إني أخذ جنسيات أخرى مصري باكستاني ولا لا . |
| رقم 2 | لا |
| رقم 3 | لا |
| رقم 2 | ورقي آه من حكيم لا ورقي من السجلات والسجلات ثابتة مكتوب أردني سوري باكستاني |
| الميسر (Facilitator) | حكيم شو بيعطي لكي . |
| رقم 3 | ما بيعطي |
| رقم 1 | أعتقد في أخرى . |
| الميسر (Facilitator) | يعني بعطي أردني غير أردني ولا أردني أخرى . |
| رقم 1 | يعطي أردني غير أردني ، لغير أردني متسلسل. |
| رقم 2 | التقرير ما ببين . |
| الميسر (Facilitator) | كم يا دكتور بهمك utilization وهلا كل الشغل والسياسات الصحية لسوريين مبنية على المنفعة ، وأنت حكيت نظرية المنفعة أقل من 2% وعلى رأأرض الواقع حقيقي أعلى ، لأنه أنا ما عم أعمله تقرير عم تطلع معي هذه النسبة. |
| رقم 4 | إلا إذا اعتمدوا غير أردني ، سوري كونه العدد كبير. |
| الميسر (Facilitator) | أغلب الغير أردنيين سوريين . |
| رقم 4 | بنسبة عالية جدا. |
| الميسر (Facilitator) | كم هذا الكلام قاعد عم بأثر على السياسات الصحية سواء كانت مركزي أو محلي . |
| رقم 4 | أكيد بأثر سلبا انت بتحكي 2% كيف أتى هذا الرقم وكيف اعتمدوه. |
| الميسر (Facilitator) | بناءا على دراسة بتعملها كل سنه .طبعا تقارير لأنه ما عنا desegregation للبيانات إدارة التأمين الصحي ما بتقدر تقول كم سوري أخذ من الخدمة، لكن اليونسف عم بجبركم تعملوا desegregation للبيانات حتى يقدر يعطيكم للبيانات . |
| رقم 4 | السوري ما بروح القطاع العسكري ولا الخدمات ولا المستشفيات الخاصة لانه غالي جدا إذا بروح على مراكزنا ومستشفياتنا . |
| الميسر (Facilitator) | معناها هذا الكلام إنه الفجوة المعلوماتية مش قادر ارصدها عم تنعكس على مشاريعي وتدريب مشاريعي . قادرين إنه نعرف موضوع الإعاقة مثلا هل النظام فيه تمييز بموضوع الإعاقة . |
| رقم 1 | فيه موجود حسب التقرير إلي بطلبوه بطلع النظام . |
| الميسر (Facilitator) | شو في بيانات موجودة عم بطلعها في التقرير. |
| رقم 6 | أخر إجتماع وإلي قبله بطلع حكيم بقول لك أعطيني ورقة فيها شو البسط أو المقام إلي بدك إياه وأنا بطلع لك إياه . |
| الميسر (Facilitator) | معظم مش المسجات المانحة الالتزامات في الأردن في الاتفاقات الدولية للوصول للفئات الخاصة منها اللاجئين ، النازحين ، ذوي الإعاقة ظن كبار السن أربع فئات أساسة مرتبطة في حقوق الإنسان وقبول للصحة كم نحن لليوم في تقاريرنا عم نعكس وصولنا للفئات هاذي ، انت بهمك كم عم بوصل لكبار السن كم عم بوصل لذوي الإعاقة . |
| رقم 4 | في المفرق في إحداهم السوريين من أكثر المحافظات ونحنا في تقاريرنا بتعطي أرقام متدنية جدا والفئات الباقية التركيز عليهم ، لو النظام بسمح يكون الأمور واضحة . |
| الميسر (Facilitator) | لما بدينا نحكي عن المشروع الاسباني وكم بدنا نعمل تعديلات للبناء من الأشياء إلي عم بنطالب من الاعتمادية يكون عنا كبار السن ، الإعاقة إلهم خصوصية وتتخذ في معايير في construction code وزارة الصحة لا يراعي هذه الفئة ، ويمكن واحد يحكي كم واحد بيجي في السنه ، إحنا بنحي أقل شي 10% من المواطنين ذوي إعاقة ، 9% من المواطنين كبار السن .كم نحن بنستجيب في التقارير ، عم أندخل data entry وrow data كثير كم بتر كبوا كلهم على شكل مؤشرات وهذه المؤشرات بتخليني أخذ السياسة ، المعلومات بشكل غير مفعل لا تعني شيء . |
| رقم 4 | بوجهة نظرك هذه الأمور شو مجال حلها . |
| الميسر (Facilitator) | أعتقد اللجنة التوجيهية للمشروع بددها تأخذ بعين الاعتبار هذا المتغيرات إلي عم نحكي فيها . |
| رقم 4 | يعني كل إلي عم نحكي فيه من تحديات وصعوبات تدونوها. |
| الميسر (Facilitator) | هلا بنطلع النتائج الأولية ما رح استنا النتائج النهائية نعقد اجتماع مع اللجنة التوجيهية نقول في فجوة حقيقية واحد إثنان ثلاثة حتى ، حكيم مبارح بحكي يا عمي نحنا ما عنا مشكله ، رقية قالت أعطوني شو ما بددكم والبرمجة أسهل شيء ، أسهل شيء أغير البرنامج. |
| رقم 1 | سهل بتعرفي كيف حتى لو بدهم يعرفوا عدد ذوي الإعاقة إلي براجع عندهم نحنا كنا زمان ولحد ألان ترميز الألوان ، استخدام الألوان ، إلي عنده مرض هيك بطلع . |
| الميسر (Facilitator) | ما ننسى إنه هذا بعكس التأمين الصحي تبعهم . |
| رقم 4 | الطريقة بسيطة سواء كان هيك أو غيرها والبرمجة بسيطة. |
| رقم 1 | لو كل ذوي إعاقة أعطوه لون معين سهل بطلع ، ببين معا من كل شاشات حكيم ، تقارير بتصير سهلة ، حكيم ما عندهم شيء صعب بس انتووا شو بدككم . |
| رقم 6 | برجع بحكي لك حكيم أعطيني سياسة أحمي حالي أمام الوزارة أنا ليه طلعتها بناءا على طلبكم . |
| الميسر (Facilitator) | بعتقد اليوم بس نيجي نحكي عن المشروع ككل ، كم اللجنة التوجيهية تؤثر على اتخاذ القرارات في المشروع نفسه بتالي في قرارات اليوم في وزارة الصحة ما كانت سابقا ، في تقارير ومؤشرات عالمية عم تنطلب من وزارة الصحة ما كانت مطلوبة سابقا ، اليوم عم بنغطي ونطلع على التقارير 2020 في كثير مؤشرات من الأردن لا توجد ، حرام كيف أنا من الأردن ومن أميز الدول في الخدمات الصحية في المنطقة لايوجد عندي بيانات ليه، المعلومة تعكس وزارة الصحة ولا تعكس الإطار الوطني . |
| رقم 4 | نحنا الان تطرقنا إلى أكثر من نقطة وتحدثنا على إنه بدنا سياسات ، وموضوع الإعاقات أنا لا أعتقد إنه في دولة في العالم يصير في هيك موضوع . دوران الملف مابين الخدمات والصحة السرية العسكرية في القصة . |
| رقم 1 | ما في سرية ولا شيء بس بخافوا ممكن من الأخطاء إلي بتصير ممكن . |
| الميسر (Facilitator) | الموضوع مش طبي . |
| رقم 4 | لما يتناقش على مستوى إلي رح يؤخذ القرار يصير في إنه نتكهن كذا وكذا ، خلينا نطرحوا بصورة مباشرة أنه وزير الصحة يتبنى هذه الفكرة ويطرحها على أصحاب القرار حتى على مستوى رئاسة الوزرة تكون محلولة . |
| الميسر (Facilitator) | موضوع نظام المعلومات في القطاع الصحي لازم يجي من فوق. |
| رقم 6 | نحن حضرنا أكثر من اجتماع من2014 من برنامج الصحة العالمي هو مظلة لكل القطاعات الصحية أيامها كان د. حكمة وهم يحكوا عن هذا اشغله نحنا بدنا ندمج والموضوع مش بيدنا ، وما زالوا هم يأخذوا القرار. |
| الميسر (Facilitator) | أنت تأخذ سياسة مبنية عن 50% من المعلومات ، تحيد كامل 50% من المعلومات من الخدمات المقدمة . |
| رقم 4 | أصحاب العلاقة ما كانوا جدين في هذا الموضوع حتى تنحل من البداية . |
| الميسر (Facilitator) | مهم جدا هذا الموضوع حتى نقدر نرصد شو توقعاتنا . |
| رقم 6 | في البداية زاروا الخدمات الطبية وزاروا القطاع الخاص. |
| الميسر (Facilitator) | د. هاني بقول مستوى الخطاب بيكون كثير أعلى من المستوى الفني ، بعتقد القرار سياسي وليس قرار فني مش مدير الخدمات لإلي بدوا يقرر. |
| رقم 4 | هل في الدول الثاني النظام الصحي الجديد نظام كان إنه مطلوب نظام سياسي إنهم يأخذوا القرار ليه هم متقدمين أكثر منا ، القطاع الصحية ما في أمور سياسية . |
| الميسر (Facilitator) | أنا هيك خلصت أسئلتي في أي شيء بتحسوا إله علاقة بنظام ، في معلومات مهمة لازم نغطيها . |
| رقم 3 | لا كل شي غطيناه ، شغلت الكوادر يعني أنا أول ما دربت على حكيم بصراحة دربتني صاحبني أنا كنت مجازة وقت التدريب على حكيم ، كنا نفتح ونعرف شو المواقع ، بعتمد على شكله التعاون إنه الكادر يكون متعاون والأطباء بداية المشروع رعاية المتكامل بداية كنا نتغلب مع الأطباء ، مع المختبر، صار في تعاون بينا وبين المختبر ، نحن ندخل الفحص بدل ما ست تفوت على المختبر على الدكتور تفوت تطبع وتوقع التعاون بحل كل القصص. |
| رقم 1 | كون فاديه مسئولة عن التزويد وعنا في كل مديرية فهي بتعاني منه لانه لغاية الآن ورقي بينما مديرية الصحة بتدقيقه الكتروني بمديرية الصحة ببين وين قي أخطاء ، إشتهاد شخصي اعمل ناه على اكسل إلها. |
| الميسر (Facilitator) | النظام المفروض مباشرة ما عي خدمة .أنا لما بحكي تنظيم أسرى هي مش خدمة وحده إحنا بنحكي مشورة بتطلع هي في تقرير وعندك تزويد ، المتابعة عندي أربع مكونات مع بعض هم تنظيم أسرى . |
| رقم 4 | الجميع بيحكي عن الكوادر خلال السنتين الماضين لما أصحاب القرارات يكونوا بعيدين عن الواقع في الميدان ، بتجمع مع الوزير بيكون من خلفياتهم ، وتحكيلهم عن دور القابلة قي المركز وإنه ضروري بيكون ف ذهنهم كيف بددي أزود الكوادر في المستشفيات بسبب كورونا ، أولويات فرضت علينا الحالة هيك ، أنت كمدير صحة بتعاني ما بتحصل على شيء ، أنا بطلت أحب أشوف بريد الوزارة ، وهو بعمل فجوة كبيرة مش قادر تحلها . |
| الميسر (Facilitator) | وكأنه صارت الوزارة معنية بكورونا ، وكم هذا القرار خلق فجوة على البرامج الثانية . |
| رقم 4 | هذا أثر سلبا علينا ، لازم يكون في كل مركز في قابلة ، يعني مش يكون قابلة لكل مركزين . |
| الميسر (Facilitator) | استفدنا من كورنا فكرة الحكومة الكترونية أنا بدفع رواتب ، مستحقاتي وفواتيري وإنا قاعد ، كورونا أعطتنا توجه إنه الشيء بتقدر تعمله الكتروني بأقل تماس مباشر مع مقدمي الخدمات . |
| رقم 4 | عن طريف المحافظ وفي كتب رسمية نجيب سائقين من مديريات ثانية لتقصي الوبائي مع إنه في فائض في مراكز أخرى ، ليش أنا بروح على المديريات ليش ما يكون في توزيع هل القترة الحالية التركيز على المستشفيات بحيث ممكن تتوفع موجه وعليها عبء كبير. |
| الميسر (Facilitator) | أكبر غلطة عملتها الوزارة اشتغلت استجابة لكورونا ، وما زلنا توجهنا على المستشفيات . توقعكم لتوسع الوطني هل برأيكم نشتغل على محافظة المفرق نربطها كلها ببعض ونحاول نوصل مع القطاعات الثانية كنموذج كامل غي محافظة بعدين نطلع لدولة ولا اكمن مركز في اربد اكم مركز في الطفيله وهكذا . |
| رقم 4 | نعتبر المفرق دولة ونعمم النظام عليها لأنه التشتيت هذا بأخذ جزئية من محافظات ثانية . |
| رقم 2 | المفرق كامل |
| رقم 4 | خلي هذا النموذج للمفرق |
| رقم 2 | حتى البيانات مرتبطة في المفرق |
| رقم 4 | كمدير صحة إداريا مسئول عن كل المراكز والمستشفيات المفروض يكون عندي شاشة واحدة إذا وصلنا في محافظة المفرق حتى الوزير والمعنيين حتى انعكاس ايجابي للخدمات الصحية في المفرق التبني رح يكون أسهل . |
| رقم 1 | صار عنا موضوع حكيم عادي للأطباء إلي يتخرجوا جداد والكوادر الصحية إلي بتخرجوا جديد ليه صار يطبق في المستشفيات ، عنا كل المستشفيات محو سبة ، كطبيب امتياز بدك تشتغل على حكيم صار يجي عنا . |
| الميسر (Facilitator) | حتى التمريض خلال دراسته طبق في المستشفيات وشاف حكيم. |
| رقم 4 | تسهل الصعوبات موجود. |
| الميسر (Facilitator) | يعني أقوى قوة ربط النظام في حكيم ، سياسة وجود حكيم موجود ، لكن أنا كم بقدر أستفيد من وجوده هو ضربة النجاح. |
| رقم 4 | أنا بذكر بدايات حكيم عنا أول ما طبقوه في قسم النسائية والأطفال ، كان في مقاومة لكن الآن إذا بتوقف بنضوج . |
| الميسر (Facilitator) | تعتقد من نجاح المشروع واستمرار يته ربطه مع حكيم . |
| رقم 6 | ربطه بشيء ثابت ما بتغير . |
| رقم 1 | بتقدري تبني عليه أي شيء بدك إياه ، وحوسبة المراكز كلها رح تعمل فرق . |
| رقم 1 | ياريت يسمحوا بدخول للكوادر الأخرى مثلا تمريض المشارك ممنوع . |
| رقم 6 | خاطبنا فيها من سنتين وما وافقوا . |
| الميسر (Facilitator) | مقدم الخدمة اله دخول. |
| رقم 4 | حصل أن اجتمعوا مع الوزير الحالي ، لو تحكوا عن هذه النقطة إنه في بعض التحفظات على ربط هذه الخدمات ، يمكن يكون هو المفتاح وعلى مستوى أعلى ونأخذ القرار الصحيح . |
| الميسر (Facilitator) | في نقاش كبير على حكيم وموضوع ربط حكيم بالمدنية مع وزارة الصحة بتالي رح نستفيد . |
| رقم 4 | حكيم عندهم بعض الأشياء إلي يتحفظوا عليها . |
| رقم 1 | بتعرفي هذه الصلاحيات إنه بس القانون هو يوثق من الوزارة . |
| الميسر (Facilitator) | حكيم بحكي أعطيني قائمة بالناس إلي الهم إدخال ، هو ما بعرف غير في القائمة إلي وصلته . |
| رقم 2 | قصة خالد بن الوليد وبدنا حد يفوت ويدرب حكوا في مشكله فيه حكينا مع حكيم وحكوا ما في مشكله جيبوا مين ما كان ، وحكوا نسبوا اسم ودربوا صيدلانيه. |
| رقم 4 | أول ما طبق حكيم في المستشفى كان في + أو – للأطفال والأمهات لازم يكون في خانة ما كانت موجودة وحطوا . |
| الميسر (Facilitator) | في البرمجة ما في شيء ما بنعمل . |
| رقم 6 | في تحفظ لدى حكيم ، إنت أعطيني شو الصلاحية إلي بدك إياها . |
| رقم 4 | ممكن يكون البرنامج موجه مش بس للقطاع الطبي ، أنت كقطاع طبي ممكن يعطوك بس إذا كانت كسياسة أعلى . |
| الميسر (Facilitator) | النظام كان كل النقاش معهم على إنه انت أعطيني قائمة مين الناس إلي بدك تعطيهم صلاحية أنا ما عندي مشكلة ، حكيم استفادوا من البرنامج وصاروا يفكروا كل برامجهم تصير قاعدة بيانات ، هم استفادوا . |
| رقم 1 | ياريت نستغني عن السجلات . |
| رقم 6 | بنستغنى عنه متى لما مديرية التأمين الصحي ومديرية المالية يتبعوا سياسات مالية صحيحة . |
