# Supplementary material for: Evaluation of a piloted digital reproductive health registry in Jordan to improve mother and child health
Source: Reprod Health. 2025 May 31;22(Suppl 1):77. doi: 10.1186/s12978-025-01995-2 (PMC12125747; doi:10.1186/s12978-025-01995-2)
Supplement: Supplementary file 5 — Supplementary material 5. Questionnaire for service users/women visiting the MCH clinics (in English language) [file 12978_2025_1995_MOESM5_ESM.docx]

**Establishing a *harmonized* Reproductive Health Registry (*h*RHR)
in Jordan to Improve Maternal and Child Health**

**End-Point Evaluation**

**Questionnaire for MCH Service User/Women**

**Demographic Information:**

Date: ____-__-__ (YYYY-MM-DD)

Health Facility (HF) Type: 🞏 CHC 🞏 PHC

Health Center Name: _____________________________

**Age:**

**Nationality:**

- Jordanian
- Syrian
- Others, Please specify--------

**Highest educational degree earned (choose one only)**

a. PhD

b. Master

c. Higher Diploma

d. Bachelor

e. Diploma

f. Other (Specify…...)

**How long have you received services in this facility?**

- First visit
- 3 months to 6 months
- 6 months to 1 year
- More than one year

**What are the services you received in this facility?**

- Vaccination for my child
- Family Planning
- Ante-natal care
- Post- natal care
- Others, define------

**Are you aware of the use the new electronic system by the health facility?**

1. Yes
2. no

| **#** | **Question** | **Strongly agree** | **Agree** | **Neutral** | **Disagree** | **Strongly disagree** | **Unable to answer** |
| --- | --- | --- | --- | --- | --- | --- | --- |
| **1** | The new electronic system speeds up the service |  |  |  |  |  |  |
| **2** | The new electronic system improves the confidentiality and privacy of my information |  |  |  |  |  |  |
| **3** | The new electronic system decreases the waiting time |  |  |  |  |  |  |
| **4** | The health staff spend more time with me |  |  |  |  |  |  |
| **5** | The health staff easily access my information through the new electronic system |  |  |  |  |  |  |
| **6** | The new electronic system makes me feel *confident* about how information about me and my health are being collected and used |  |  |  |  |  |  |
| **7** | The new electronic system makes me feel *uncomfortable* about who can see my data and how they will use it. |  |  |  |  |  |  |
| **8** | The new electronic system has made my visits to the health facility *more daunting/ difficult*: |  |  |  |  |  |  |
| **8.1** | Probe: Please explain how: |  | | | | | |
| **9** | The new electronic system has improved my relationship with my provider |  |  |  |  |  |  |
| **10** | The new electronic system has made my visits to the health center faster compared to the paper-based file |  |  |  |  |  |  |
| **11** | The new electronic system has made me feel confident that each subsequent visit will be based on my previous health data captured in the system. |  |  |  |  |  |  |
| **12** | I would like to have access to my medical files to the new electronic system through a mobile app |  |  |  |  |  |  |
| **13** | Please describe if you have any specific comments or suggestions about this new electronic system. | *Capture the answer here:* | | | | | |
