# Supplementary material for: Evaluation of a piloted digital reproductive health registry in Jordan to improve mother and child health
Source: Reprod Health. 2025 May 31;22(Suppl 1):77. doi: 10.1186/s12978-025-01995-2 (PMC12125747; doi:10.1186/s12978-025-01995-2)
Supplement: Supplementary file 6 — Supplementary material 6. Self-administered questionnaire for service providers at MCH clinics (in English language) [file 12978_2025_1995_MOESM6_ESM.docx]

**Establishing a *harmonized* Reproductive Health Registry (*h*RHR)
in Jordan to Improve Maternal and Child Health**

**End-Point Evaluation**

**Questionnaire for Service Providers**

**Demographic Information:**

Date: ____-__-__ (YYYY-MM-DD)

Health Facility (HF) Type: 🞏 CHC 🞏 PHC

Health Center Name: _____________________________

Respondent’s Email: _____________________________(Note: This information is just for the purpose of contact with you. It will be removed during the analysis. Also, there will be no mention of name or any other identifiable information.)

Respondent’s Code: _______

Age:

Sex:

□ Male,

□ Female

Highest educational degree earned (choose one only)

a. PhD

b. Master

c. Higher Diploma

d. Bachelor

e. Diploma

f. Other (Specify…...)

Job title:

1. Gynecologist
2. GP
3. Nurse
4. Midwife
5. Receptionist
6. Others, specify

Your work:

1. Full time at the piloted facility
2. Part time visiting staff at the piloted facility, specify average spent in the facility by hours)
3. Other work arrangement (e.g., supervisory role, technical support etc.)

How long have you worked in this facility?

1. Less than 3 months
2. 3 months to 1 year
3. 1 to 5 years
4. More than 5 years

Do you use the new electronic system?

1. Yes
2. no

How long have you used the new electronic system?

1. Less than 3 months
2. 3-6 months
3. 6-12 months
4. others

| **Item** | **Question** | **Strongly agree** | **Agree** | **Neutral** | **Disagree** | **Strongly disagree** | **Unable to answer** |
| --- | --- | --- | --- | --- | --- | --- | --- |
| **Acceptability** | The new electronic system is more favorable than paper records |  |  |  |  |  |  |
|  | The new electronic system enables quick access to patient records for more coordinated and efficient care |  |  |  |  |  |  |
|  | The new electronic system helps providers to improve their productivity and work balance |  |  |  |  |  |  |
|  | Using the new electronic system is not time consuming |  |  |  |  |  |  |
|  | The new electronic system does NOT impede the daily work protocol |  |  |  |  |  |  |
|  | The Information from the new electronic system is relevant to our daily work |  |  |  |  |  |  |
|  | The new electronic system enhances privacy and security of patient data |  |  |  |  |  |  |
|  | In our health facility, all service providers have willingness to keep using this system |  |  |  |  |  |  |
|  | I am satisfied with the new electronic system |  |  |  |  |  |  |
| **Usefulness** | The new electronic system provides accurate, up-to-date, and complete information about patients at the point of care |  |  |  |  |  |  |
|  | The new electronic system helps promote legible, complete, and accurate documentation |  |  |  |  |  |  |
|  | The new system provides documentation to all PMNCH services |  |  |  |  |  |  |
|  | The new electronic system helps in reducing medical errors |  |  |  |  |  |  |
|  | The new electronic system allows secure sharing of patient’s information with other authorized health facilities |  |  |  |  |  |  |
|  | The new electronic system improved communication with other health service providers |  |  |  |  |  |  |
|  | The information from the new electronic system is presented in a useful format and can be retrieved easily. |  |  |  |  |  |  |
|  | Using the new system enhances the continuity of care that our health facility provided to the patients |  |  |  |  |  |  |
|  | As a service provider, I think the new electronic system is useful |  |  |  |  |  |  |
| **Sustainability** | The web-based application works in synchronization with the core system used in the MoH centers and hospitals |  |  |  |  |  |  |
|  | The new electronic health system is easy to learn. |  |  |  |  |  |  |
|  | The support services for the new electronic system are dependable |  |  |  |  |  |  |
|  | The support services for the new electronic system is available within MOH/Central and peripheral level |  |  |  |  |  |  |
|  | The new electronic system’s users have access to ongoing training by the MOH |  |  |  |  |  |  |
|  | The automated MCH cards used in the new electronic system are the same as the ones used in other automated and un-automated health facilities |  |  |  |  |  |  |
|  | The MOH provides training for new staff on using the new electronic system |  |  |  |  |  |  |
|  | The new electronic system can accommodate new variables/fields with minimum cost and efforts |  |  |  |  |  |  |
|  | The new electronic system can generate needed periodic reports and relevant indicators |  |  |  |  |  |  |
| **Governance/data security** | The data are safe and secure, and you can access the data according to strict authorization |  |  |  |  |  |  |
|  | The new electronic system data ownership is clearly defined by the developer |  |  |  |  |  |  |
|  | The new electronic system a clear guideline for data users. (What can the data be used for? What uses are prohibited?) |  |  |  |  |  |  |
|  | The new electronic system has a clear Data-Sharing guideline |  |  |  |  |  |  |
|  | The new electronic system has a validation capacity (ensure correct and complete data entry) |  |  |  |  |  |  |
| **Feasibility** | The new electronic system can be used in any MoH PHCs |  |  |  |  |  |  |
|  | The new electronic system can be used in any MoH hospitals |  |  |  |  |  |  |
|  | The new electronic system can be used in other non-MOH facilities |  |  |  |  |  |  |
|  | Health facilities can access the new electronic system using only a laptop/desktop and regular internet connection. |  |  |  |  |  |  |
|  | The new electronic system is available on any internet browser and no software is required to be installed |  |  |  |  |  |  |
|  | The new electronic system provides the same reports as those used and generated manually at the central level MOH health facilities |  |  |  |  |  |  |
|  | Data from new electronic system is stored on the existing servers, whereby access is provided to all authorized stakeholders and users |  |  |  |  |  |  |
|  | The new electronic system reduces costs through decreased paperwork, improved safety, reduced duplication of interventions eg. Testing and medications |  |  |  |  |  |  |
| **Simplicity** | As a service provider, I can access patient’s profile using the national number for Jordanian patients or a unique patient ID for non-Jordanian |  |  |  |  |  |  |
|  | Authorized service providers need only a username and password to access the new electronic system using |  |  |  |  |  |  |
|  | The information flow of the new electronic system is easy to understand |  |  |  |  |  |  |
|  | The user interface in the new electronic system is simple and easy to understand |  |  |  |  |  |  |
|  | The interface in the new electronic system is simple with minimum needed training |  |  |  |  |  |  |
|  | The new electronic system uses the same information and fields of the same paper based MOH patient cards |  |  |  |  |  |  |
|  | Data entry to the new electronic system is not time consuming |  |  |  |  |  |  |
| **Human Rights** | The new electronic system can help identify gaps in accessibility among different groups |  |  |  |  |  |  |
|  | The new electronic system can help identify gaps in continuity of care within the health delivery system. |  |  |  |  |  |  |
|  | The new electronic system helps to assess the association between sociodemographic determinants of and access/utilization of services. |  |  |  |  |  |  |
|  | The new electronic system improves privacy and confidentiality of patients |  |  |  |  |  |  |
|  | The information and indicators generated by the new electronic system will help to improve quality of care provided |  |  |  |  |  |  |
|  | The new electronic system can be utilized to define special needs of vulnerable groups |  |  |  |  |  |  |
|  | The patient has the right to access the new electronic system |  |  |  |  |  |  |
|  | The new electronic system empowers patients to make informed decisions related to their health conditions |  |  |  |  |  |  |
| **Gender** | The new electronic system improves the collection of sex-disaggregated data (data based on sex) |  |  |  |  |  |  |
|  | *Probe:* Please explain if it is due the efficiency of collection and/or the quality of data captured? | | | | | | |
|  | The new electronic system improves the analysis of sex-disaggregated data (data based on sex) – |  |  |  |  |  |  |
|  | *Probe:* Given you answer above, what additional analysis insights can (or cannot) be possible in this new electronic system? | | | | | | |
|  | The new electronic system is adding to workload, creating new hierarchies. |  |  |  |  |  |  |
|  | The new electronic system is creating challenges in power dynamics and gender. |  |  |  |  |  |  |
|  | The new electronic system improves privacy and confidentiality of patients (e.g. it is password protected so providers need to be validated before seeing the data) |  |  |  |  |  |  |
|  | The new electronic system covers all components of SRH; including STIs/HIV, Sexuality, GBV etc. in a seamless/ logical way |  |  |  |  |  |  |
|  | *Probe:* Please indicate which of the above components of SRH or not covered; and, what is your suggestion on how to include/cover them? | | | | | | |
|  | The new electronic system can help collect data about accessibility among different groups |  | | | | | |
|  | (Men, Women, Boys and Girls) |  |  |  |  |  |  |
|  | (Married/unmarried) |  |  |  |  |  |  |
|  | (Adolescent/reproductive age/post-menopausal) |  |  |  |  |  |  |
|  | The new electronic system can help with the analysis to identify gaps in accessibility among different groups |  | | | | | |
|  | (Men, Women, Boys and Girls) |  |  |  |  |  |  |
|  | (Married/unmarried) |  |  |  |  |  |  |
|  | (Adolescent/reproductive age/post-menopausal) |  |  |  |  |  |  |
|  | The new electronic system logically and clearly presents associations among the following sociodemographic determinants and access/utilization of services. |  | | | | | |
|  | Education |  |  |  |  |  |  |
|  | Economic status |  |  |  |  |  |  |
|  | Religion |  |  |  |  |  |  |
|  | Nationality |  |  |  |  |  |  |
|  | The new electronic system can be utilized to define special needs of the following vulnerable groups |  | | | | | |
|  | Refugees-IDPs- Immigrants |  |  |  |  |  |  |
|  | People with disabilities |  |  |  |  |  |  |
|  | Marginalized and other vulnerable groups |  |  |  |  |  |  |
|  | The new electronic system helps in assessing the differences in morbidities and mortalities based on gender |  |  |  |  |  |  |
|  | The new electronic system empowers patients to make informed decisions related to their health conditions |  |  |  |  |  |  |
| Are there any functionalities missing in the new electronic system? If yes, please specify? | | | | | | |  |
| Are there any functionalities need to be improved? If yes, please specify? | | | | | | |  |
| Compared with the paper-based documentation, what are the benefits of the new electronic system? To the health provider, to the patient? | | | | | | |  |
| Do you think the new electronic system improves the health service delivery? please specify? | | | | | | | |
| What are your recommendations for improvement?  Access:  Functionalities and scope:  Utilization of health providers:  Reporting functions: | | | | | | | |
