# Supplementary material for: Evaluation of a piloted digital reproductive health registry in Jordan to improve mother and child health
Source: Reprod Health. 2025 May 31;22(Suppl 1):77. doi: 10.1186/s12978-025-01995-2 (PMC12125747; doi:10.1186/s12978-025-01995-2)
Supplement: Supplementary file 7 — Supplementary material 7. Focus group discussion (FGD) guide for stakeholders (in English language) [file 12978_2025_1995_MOESM7_ESM.docx]

**Establishing a *harmonized* Reproductive Health Registry (*h*RHR)
in Jordan to Improve Maternal and Child Health**

**End-Point Evaluation**

**Focus Group Discussion (FGD) Guide for Stakeholders**

**Demographic Information:**

**Please fill out the table below for each FGD participants as per the demographic info questions that follow the table:**

|  | **Respondents** | | | | | | | | |
| --- | --- | --- | --- | --- | --- | --- | --- | --- | --- |
| Date | ____-__-__ (YYYY-MM-DD) | | | | | | | | |
| Respondent Code (###): | R1 | R2 | R3 | R4 | R5 | R6 | R7 | R8 | Add as needed |
| **Respondent’s Email:** |  |  |  |  |  |  |  |  |  |
| Age |  |  |  |  |  |  |  |  |  |
| Gender |  |  |  |  |  |  |  |  |  |
| Highest Education Degree obtained |  |  |  |  |  |  |  |  |  |
| Job Position |  |  |  |  |  |  |  |  |  |
| Department |  |  |  |  |  |  |  |  |  |
| Informed Consent to Obtained? |  |  |  |  |  |  |  |  | YES/NO |
| Consent on voice recording obtained? |  |  |  |  |  |  |  |  | YES/NO |

Thank you for agreeing to participate in this group discussion. We are looking forward to hearing your valuable perspectives and learning from you. The purpose of this discussion is to learn more from you about the new electronic Registry (*h*RHR).

***Additional instructions to the FGD Moderator:*** *Remember to actively use probe questions to get more details about any interesting answers that may be of value to further explore. Please refrain from mentioning names; only use codes, and remove email addresses or any other identifiable information before the data analysis. Also, remember to record in the table above (for each FGD participant separately) that consent was obtained.*

*****

**Please ask the following details separately for each FGD participating stakeholder and fill out in the table above.**

Date: ____-__-__ (YYYY-MM-DD)

**Respondent’s Email: _____________________________(Note: This information is just for the purpose of contact with you. It will be removed during the analysis. Also, there will be no mention of name or any other identifiable information.)**

**Respondent’s Code: _______**

**Age:**

**Sex:**

**□ Male, □ Female**

**Highest educational degree earned (choose one only)**

a. PhD

b. Master

c. Higher Diploma

d. Bachelor

e. Diploma

f. Other (Specify…...)

**Job Position:**

**Department:**

**How long have you been in your position?**

| **No.** | **Question** | **Answer** |
| --- | --- | --- |
|  | In general, how would you rate the new electronic health system? |  |
|  | In general, how would you rate the new electronic health system compared to the paper records used in the past? |  |
|  | What are the benefits and effects of introducing and implementing the new electronic *h*RHR? |  |
|  | Do you think the new system fulfills the main objective of bridging the information gap between different levels of care? How? |  |
|  | In your opinion, what are the most important attributes or components of the new electronic *h*RHR system? |  |
|  | In your opinion, what are the most important challenges of using the new electronic *h*RHR system? |  |
|  | Do you think the new electronic system increased the quality of women and child health healthcare? How? |  |
|  | Do you think that the new electronic system Is cost-effective? Please elaborate |  |
|  | Do you think that the new system is a sustainable tool that can contribute to measures taken to improve reproductive health outcomes? How? |  |
|  | Do you think that the new system is an acceptable tool for the relevant stakeholders, service providers, and service users? why? |  |
|  | Do you think that the new system is an expandable and adoptable tool on a national level? |  |
|  | What makes the new system an expandable and adoptable tool on a national level? |  |
|  | Do you think using the new system as tool for providing timely actionable data for health providers would improve the provided MCH care? How? Please elaborate? |  |
|  | Do you think that the new system will improve the patent satisfaction of the services received? How? Please elaborate? |  |
|  | Do you think that implementing the *h*RHR affected the referral decision at different levels within the health care facility? |  |
|  | Do you think that the new electronic *h*RHR lead to a more effective and efficient referral system with other services? How? Please elaborate |  |
|  | What referral information is available and utilized at the different levels of the health system when women seek reproductive health care? |  |
|  | How will implementing *h*RHR affect the referral decision at different levels of health care? |  |
|  | How will implementing *h*RHR affect the referral of women seeking reproductive health services? |  |
|  | What are the attributes or components of the current reproductive referral system and how can *h*RHR lead to a more effective and efficient referral system? |  |
|  | Do you think the new system facilitates generating required MCH information and reports? How? |  |
|  | What are the weaknesses/ limitations of the new *h*RHR system? |  |
|  | Arere there any functionalities need to be improved? If yes, please specify? |  |
| **Questions on Gender Integration** | | |
|  | Does the new electronic system improve the collection and analysis of sex-disaggregated data (data based on sex) compared to the paper-based system?  ***Probe questions:***  If so, please describe how.  I If not, please suggest how this can be ensured? |  |
|  | Does the electronic system collects and analyzes gender-disaggregated data (data collected by sex, age, marital status, ability, location and other intersectional identity markers of an individual)?  ***Probe questions:***  If so, please describe how.  I If not, please suggest how this can be ensured? |  |
|  | Does the new electronic system help identify gaps in accessibility among different groups?   - (Men, Women, Boys and Girls) - (Married/unmarried) - (Adolescent/reproductive age/post-menopausal).   ***Probe questions:***  If so, please describe how it helps.  If not, please suggest how this can be ensured within the new electronic *h*RHR system? |  |
|  | Does the new electronic system help to assess the association between sociodemographic determinants of and access/utilization of services for better health policies?   - Education - Economic status - Religion - Nationality   ***Probe questions:***  If so, please describe how it helps.  If not, please suggest how this can be ensured within the new electronic *h*RHR system? |  |
|  | When making policies to be more inclusive for Refugees-IDPs, im(migrants) and people with disabilities, would it be useful to have the electronic system provide you with specific comparisons and analysis?  ***Probe questions:***  If so, please describe how it can be utilized.  If not, please suggest how this issue can be addressed within the new electronic *h*RHR system? |  |
|  | Do you think the new electronic system covers all components of SRH in a way that is meaningful to you and your work? Including:   - STIs/HIV - Sexuality - GBV - Others (specify)   ***Probe questions:***  If so, please describe how it covers those components.  If not, please suggest how relevant gaps can be addressed within the new electronic *h*RHR system? |  |
|  | Finally, are there any functionalities missing in the new electronic system? If yes, please specify? |  |
|  | Do you have any suggestions or recommendations about the system? |  |
