# Supplementary material for: Evaluation of a piloted digital reproductive health registry in Jordan to improve mother and child health
Source: Reprod Health. 2025 May 31;22(Suppl 1):77. doi: 10.1186/s12978-025-01995-2 (PMC12125747; doi:10.1186/s12978-025-01995-2)
Supplement: Supplementary file 8 — Supplementary material 8. Questionnaire for service users/women visiting the MCH clinics (in Arabic language) [file 12978_2025_1995_MOESM8_ESM.docx]

**إنشاء سجل منسق للصحة الإنجابية**

**في الأردن لتحسين صحة الأم والطفل**

**تقييم نقطة تقديم الخدمة**

**استبيان لمستخدمي خدمة صحة الأم والطفل/ النساء**

**البيانات الديموغرافية:**

التاريخ:
نوع المنشأة الصحية:

- مركز صحي اولي
- مركز صحي شامل

اسم المركز الصحي:

العمر:

الجنسية:

- أردنية
- سورية
- غير ذلك، يرجى التحديد ______

**أعلى درجة علمية حصلت عليها (اختر واحدة فقط)**

أ. دكتوراه

ب. ماجستير

ج. دبلوم عالي

د. بكالوريوس

ه. شهادة دبلوم

و. غير ذلك، الرجاء التحديد .................

**منذ متى وأنت تتلقين خدمات في هذا المركز الصحي؟**

- أول زيارة
- 3-6 أشهر
- 6 أشهر - سنة
- أكثر من سنة

**ما هي الخدمات التي تلقيتها في هذا المركز؟**

- تطعيم طفلي
- تنظيم الأسرة
- الرعاية قبل الولادة
- الرعاية بعد الولادة-النفاس
- غير ذلك، يرجى التحديد_______

**هل أنت على معرفة باستخدام النظام الإلكتروني الجديد للمركز الصحي:**

1. نعم
2. لا

إذا كانت الإجابة ب"نعم" يرجى الإجابة على الأسئلة التالية

| **#** | **السؤال** | **أوافق بشدة** | **أوافق** | **محايد** | **لا أوافق** | **لا أوافق بشدة** | **عاجز عن الإجابة** |
| --- | --- | --- | --- | --- | --- | --- | --- |
| **1** | **النظام الإلكتروني الجديد يسرّع الخدمة** |  |  |  |  |  |  |
| **2** | **النظام الإلكتروني الجديد يتصف بالسرية ويحافظ على خصوصية معلوماتي** |  |  |  |  |  |  |
| **3** | **النظام الإلكتروني الجديد يقلل من وقت الانتظار** |  |  |  |  |  |  |
| **4** | **يقضي مقدمو الخدمات الصحية المزيد من الوقت معي** |  |  |  |  |  |  |
| **5** | **يمكن لمقدمي الخدمات الصحية الوصول بسهولة إلى معلوماتي من خلال النظام الإلكتروني الجديد** |  |  |  |  |  |  |
| **6** | **يشعرني النظام الإلكتروني الجديد بثقة حول كيفية جمع واستخدام المعلومات الخاصة بي وبصحتي** |  |  |  |  |  |  |
| **7** | **جعل النظام الإلكتروني الجديد زياراتي للمركز الصحي أكثر صعوبة** |  |  |  |  |  |  |
| **7.1** | **أسئلة استكشافية: يرجى توضيح ذلك:** |  |  |  |  |  |  |
| **8** | **حسّن النظام الإلكتروني الجديد علاقتي مع مقدم الخدمة الخاص بي** |  |  |  |  |  |  |
| **9** | **جعل النظام الإلكتروني الجديد زياراتي للمركز الصحي أسرع مقارنة بعملية استخدام الملف الورقي** |  |  |  |  |  |  |
| **10** | **ساهم النظام الالكتروني الجديد بان اشعر بالثقة في أن كل زيارة مستقبلية ستستند على بياناتي الصحية السابقة في النظام.** |  |  |  |  |  |  |
| **11** | **أرغب ان اتمكن من الوصول إلى ملفاتي الطبية على النظام الإلكتروني الجديد من خلال تطبيق الهاتف المحمول** |  |  |  |  |  |  |
| **12** | **يرجى توضيح ما إذا كان لديك أي تعليقات أو اقتراحات محددة حول هذا النظام الإلكتروني الجديد** |  |  |  |  |  |  |
