# Supplementary material for: Evaluation of a piloted digital reproductive health registry in Jordan to improve mother and child health
Source: Reprod Health. 2025 May 31;22(Suppl 1):77. doi: 10.1186/s12978-025-01995-2 (PMC12125747; doi:10.1186/s12978-025-01995-2)
Supplement: Supplementary file 9 — Supplementary material 9. Self-administered questionnaire for service providers at MCH clinics (in Arabic language) [file 12978_2025_1995_MOESM9_ESM.docx]

**أسئلة التقييم لمقدمي الخدمات:**

**المعلومات الديموغرافية:**

**التاريخ:**

**نوع المنشأة الصحية:**

- **مركز صحي اولي**
- **مركز صحي شامل**

**اسم المركز الصحي:**

**البريد الإلكتروني لمقدم الخدمة:**

**(ملاحظة: هذه المعلومات هي لغرض التواصل معك فقط. سيتم حذفها أثناء مرحلة التحليل، كما لن يكون هناك ذكر لأي اسم أو أي معلومات أخرى.)**

**رمز مقدم الخدمة:**

**العمر :**

**الجنس:**

- ذكر
- أنثى

**أعلى درجة علمية حصلت عليها (اختر واحدة فقط)**

أ. دكتوراه

ب. ماجستير

ج. دبلوم عالي

د. بكالوريوس

ه. شهادة دبلوم

و. غير ذلك (الرجاء التحديد..........................................)

**الوظيفة**

- طبيب نسائية
- طبيب عام
- ممرض
- قابلة قانونية
- موظف/ة استقبال
- غير ذلك، _____________

**عملك:**

- دوام كامل في المركز الصحي
- موظف زائر بدوام جزئي في المرفق الصحي، حدد متوسط العمل في المرفق بالساعات ----------
- ترتيبات عمل أخرى (مثل الدور الإشرافي والدعم التقني وما إلى ذلك)

**منذ متى وأنت تعمل في هذه المركز الصحي؟**

- أقل من 3 أشهر
- من 3 أشهر إلى سنة
- من 1 إلى 5 سنوات
- أكثر من 5 سنوات

**هل تستخدم النظام الإلكتروني الجديد؟**

- نعم
- لا

**منذ متى وأنت تستخدم النظام الإلكتروني الجديد؟**

- أقل من 3 أشهر
- 3-6 أشهر
- 6-12 شهر
- غير ذلك.

| **البند** | **السؤال** | **أوافق بشدة** | **أوافق** | **محايد** | **لا أوافق** | **لا أوافق بشدة** | **عاجز عن الإجابة** |
| --- | --- | --- | --- | --- | --- | --- | --- |
| **درجة القبول** | يعتبر النظام الإلكتروني الجديد أكثر ملاءمة من السجلات الورقية |  |  |  |  |  |  |
|  | يتيح النظام الإلكتروني الجديد الوصول السريع إلى سجلات المرضى للحصول على رعاية أكثر تنسيقا وكفاءة |  |  |  |  |  |  |
|  | يساعد النظام الإلكتروني الجديد مقدمي الخدمات على تحسين إنتاجيتهم وتوازن عملهم |  |  |  |  |  |  |
|  | استخدام النظام الإلكتروني الجديد لا يستغرق وقتا طويلا |  |  |  |  |  |  |
|  | النظام الإلكتروني الجديد لا يعيق برنامج العمل اليومي |  |  |  |  |  |  |
|  | المعلومات من النظام الإلكتروني الجديد ذات صلة بعملنا اليومي |  |  |  |  |  |  |
|  | يعزز النظام الإلكتروني الجديد خصوصية وأمن بيانات المرضى |  |  |  |  |  |  |
|  | لدى جميع مقدمي الخدمات الرغبة للحفاظ على استخدام هذا النظام في مركزنا الصحي |  |  |  |  |  |  |
|  | أنا راض عن النظام الإلكتروني الجديد |  |  |  |  |  |  |
| **الجدوى** | يوفر النظام الإلكتروني الجديد معلومات دقيقة ومحدثة وكاملة عن المرضى في مرحلة تقديم الخدمة |  |  |  |  |  |  |
|  | يساعد النظام الإلكتروني الجديد على تعزيز التوثيق بشكل واضح وكامل ودقيق |  |  |  |  |  |  |
|  | يوفر النظام الجديد التوثيق الكامل لكافة خدمات صحة الأمهات والمواليد الجدد والأطفال |  |  |  |  |  |  |
|  | النظام الإلكتروني الجديد يساعد في الحد من الأخطاء الطبية |  |  |  |  |  |  |
|  | يسمح النظام الإلكتروني الجديد بالتبادل الآمن لمعلومات المريض مع المرافق الصحية الأخرى المعتمدة |  |  |  |  |  |  |
|  | يحسن النظام الإلكتروني الجديد التواصل مع مقدمي الخدمات الصحية الآخرين |  |  |  |  |  |  |
|  | تعرض المعلومات من النظام الإلكتروني الجديد بشكل سلس ويمكن استرجاعها بسهولة. |  |  |  |  |  |  |
|  | استخدام النظام الجديد يعزز استمرارية الرعاية التي يقدمها مرفقنا الصحي للمرضى |  |  |  |  |  |  |
|  | أعتقد كمزود خدمة أن النظام الإلكتروني الجديد مفيد |  |  |  |  |  |  |
| **الاستدامة** | يعمل التطبيق الإلكتروني بتوافق مع أنظمة المعلومات الاخرى المستخدمة في مراكز وزارة الصحة والمستشفيات |  |  |  |  |  |  |
|  | من السهل تعلم كيفية استخدام النظام الإلكتروني الجديد. |  |  |  |  |  |  |
|  | يمكن الاعتماد على خدمات الدعم المتوفرة للنظام الإلكتروني الجديد |  |  |  |  |  |  |
|  | تتوفر خدمات دعم النظام الإلكتروني الجديد على مستوى وزارة الصحة/ مركز الوزارة والمديريات الميدانية |  |  |  |  |  |  |
|  | يمكن لمستخدمي النظام الإلكتروني الجديد الحصول على التدريب المستمر من خلال وزارة الصحة |  |  |  |  |  |  |
|  | بطاقات صحة الأم والطفل الآلية المستخدمة في النظام الإلكتروني الجديد هي نفسها المستخدمة في المرافق الصحية الأخرى المحوسبة وغير المحوسبة |  |  |  |  |  |  |
|  | تقدم وزارة الصحة التدريب على استخدام النظام الإلكتروني الجديد للموظفين الجدد |  |  |  |  |  |  |
|  | يمكن للنظام الإلكتروني الجديد استيعاب المتغيرات/الحقول الجديدة المطلوبة بأقل تكلفة وجهد |  |  |  |  |  |  |
|  | يمكن للنظام الإلكتروني الجديد أن يُنتج التقارير الدورية اللازمة والمؤشرات ذات الصلة |  |  |  |  |  |  |
| **الحوكمة/أمن البيانات** | البيانات آمنة ومضمونة ويمكنك الوصول إلى البيانات وفقاً لتصريح محدد |  |  |  |  |  |  |
|  | تُعرف ملكية بيانات النظام الإلكتروني الجديد بوضوح من قبل المطور |  |  |  |  |  |  |
|  | يقدم النظام الإلكتروني الجديد دليلا واضحا لمستخدمي البيانات. (ما الذي يمكن استخدام البيانات من أجله؟ ما هي الاستخدامات المحظورة؟) |  |  |  |  |  |  |
|  | لدى النظام الإلكتروني الجديد معايير واضحة لتبادل البيانات |  |  |  |  |  |  |
|  | لدى النظام الإلكتروني الجديد القدرة على التحقق (ضمان إدخال البيانات بشكل صحيح وكامل) |  |  |  |  |  |  |
| **الجدوى** | يمكن استخدام النظام الإلكتروني الجديد في أي من مراكز الرعاية الصحية في وزارة الصحة |  |  |  |  |  |  |
|  | يمكن استخدام النظام الإلكتروني الجديد في أي من مستشفيات وزارة الصحة |  |  |  |  |  |  |
|  | يمكن استخدام النظام الإلكتروني الجديد في مرافق أخرى غير وزارة الصحة |  |  |  |  |  |  |
|  | تحتاج المرافق الصحية للوصول إلى النظام الإلكتروني الجديد إلى جهاز كمبيوتر محمول/الحاسوب المكتبي فقط واتصال منتظم بالإنترنت. |  |  |  |  |  |  |
|  | النظام الإلكتروني الجديد متاح على أي متصفح للإنترنت وليس هناك حاجة إلى أي برمجية ليتم تحميله |  |  |  |  |  |  |
|  | يقدم النظام الإلكتروني الجديد نفس التقارير التي يتم استخدامها وانتاجها يدويًا في المرافق الصحية لوزارة الصحة |  |  |  |  |  |  |
|  | تخزن البيانات المستمدة من النظام الإلكتروني الجديد على الخوادم المتوفرة حالياً حيث تتاح إمكانية الوصول اليها لجميع أصحاب المصلحة والمستخدمين الذين يمتلكون الصلاحيات لذلك |  |  |  |  |  |  |
|  | يقلل النظام الإلكتروني الجديد من التكاليف من خلال التقليل من استخدام الأوراق وتحسين السلامة والحد من الازدواجية في التدخلات على سبيل المثال: الفحوصات والأدوية. |  |  |  |  |  |  |
| **البساطة** | يمكنني كمقدم خدمة الوصول إلى ملف المريض باستخدام الرقم الوطني للمرضى الأردنيين أو بطاقة هوية معينة لغير الأردنيين |  |  |  |  |  |  |
|  | يحتاج مقدمو الخدمات المعتمدون إلى اسم مستخدم وكلمة مرور فقط للوصول إلى النظام الإلكتروني الجديد |  |  |  |  |  |  |
|  | من السهل فهم طريقة تدفق المعلومات من النظام الإلكتروني الجديد |  |  |  |  |  |  |
|  | صفحة المستخدم في النظام الإلكتروني الجديد بسيطة وسهلة الفهم |  |  |  |  |  |  |
|  | الصفحات في النظام الإلكتروني الجديد بسيطة وتتطلب حد أدنى من التدريب |  |  |  |  |  |  |
|  | يستخدم النظام الإلكتروني الجديد نفس المعلومات والحقول في بطاقات مرضى وزارة الصحة الورقية |  |  |  |  |  |  |
|  | لا يستغرق إدخال البيانات إلى النظام الإلكتروني الجديد وقتا طويلا |  |  |  |  |  |  |
| **حقوق الإنسان** | يمكن للنظام الإلكتروني الجديد أن يساعد في تحديد الثغرات في إمكانية الوصول للخدمات بين مختلف الفئات المستهدفة |  |  |  |  |  |  |
|  | يمكن للنظام الإلكتروني الجديد أن يساعد في تحديد الثغرات في استمرارية الرعاية داخل نظام تقديم الخدمات الصحية. |  |  |  |  |  |  |
|  | يساعد النظام الإلكتروني الجديد على تقييم العلاقة بين المحددات الاجتماعية الديمغرافية والوصول إلى الخدمات/الاستفادة منها. |  |  |  |  |  |  |
|  | النظام الإلكتروني الجديد يحسن خصوصية وسرية المرضى |  |  |  |  |  |  |
|  | ستساعد المعلومات والمؤشرات التي يولدها النظام الإلكتروني الجديد على تحسين نوعية الرعاية المقدمة |  |  |  |  |  |  |
|  | يمكن استخدام النظام الإلكتروني الجديد لتحديد الاحتياجات الخاصة للفئات الخاصة |  |  |  |  |  |  |
|  | يحق للمريض الوصول إلى النظام الإلكتروني الجديد |  |  |  |  |  |  |
|  | يمكّن النظام الإلكتروني الجديد المرضى من اتخاذ قرارات مستنيرة تتعلق بأوضاعهم الصحية |  |  |  |  |  |  |
| **النوع الاجتماعي** | يحسن النظام الإلكتروني الجديد جمع البيانات المصنفة حسب النوع الاجتماعي |  |  |  |  |  |  |
|  | الأسئلة الاستكشافية: يرجى توضيح ما إذا كان ذلك بسبب كفاءة تحصيل البيانات أو جودتها؟ |  |  |  |  |  |  |
|  | يحسن النظام الإلكتروني الجديد جمع البيانات المصنفة حسب النوع الاجتماعي |  |  |  |  |  |  |
|  | الأسئلة الاستكشافية: بالنظر إلى الإجابة أعلاه، ما هي المميزات الإضافية التي يمكن (أو لا يمكن) أن تكون ممكنة في هذا النظام الإلكتروني الجديد؟ |  |  |  |  |  |  |
|  | يضيف النظام الإلكتروني الجديد إلى عبء العمل، يخلق تسلسلات هرمية جديدة. |  |  |  |  |  |  |
|  | يخلق النظام الإلكتروني الجديد تحديات في ديناميكيات السلطة والجنس. |  |  |  |  |  |  |
|  | يعمل النظام الإلكتروني الجديد على تحسين الخصوصية والسرية للمرضى (على سبيل المثال: حماية النظام بكلمة مرور، لذلك يحتاج مقدمو الخدمة إلى إثبات هويتهم قبل مشاهدة البيانات) |  |  |  |  |  |  |
|  | يغطي النظام الإلكتروني الجديد جميع مكونات الصحة الإنجابية؛ بما في ذلك الأمراض المنقولة جنسيا/ فيروس نقص المناعة البشرية والصحة الجنسية والعنف القائم على النوع الاجتماعي وما إلى ذلك بطريقة سلسة / منطقية. |  |  |  |  |  |  |
|  | أسئلة استكشافية: يرجى تحديد أي من المكونات المذكورة أعلاه من الصحة الإنجابية أو التي لم تتم تغطيتها؛ وما هو اقتراحك حول كيفية تغطيتها؟ |  |  |  |  |  |  |
|  | يمكن للنظام الإلكتروني الجديد أن يساعد في جمع البيانات حول إمكانية الوصول بين المجموعات المختلفة. |  | | | | | |
|  | 1. رجال، نساء، بنين وبنات |  |  |  |  |  |  |
|  | 1. متزوج / غير متزوج |  |  |  |  |  |  |
|  | 1. المراهقات / سن الإنجاب / سن الأمل |  |  |  |  |  |  |
|  | يمكن للنظام الإلكتروني الجديد أن يساعد في التحليل لتحديد الفجوات في إمكانية الوصول بين المجموعات المختلفة. |  | | | | | |
|  | 1. رجال، نساء، بنين وبنات |  |  |  |  |  |  |
|  | 1. متزوج / غير متزوج |  |  |  |  |  |  |
|  | 1. المراهقات / سن الإنجاب / سن الأمل |  |  |  |  |  |  |
|  | النظام الإلكتروني الجديد بشكل منطقي وواضح ارتباطات بين المحددات الاجتماعية والديموغرافية التالية والوصول / الاستفادة من الخدمات. |  | | | | | |
|  | 1. التعليم |  |  |  |  |  |  |
|  | 1. الحالة الاقتصادية |  |  |  |  |  |  |
|  | 1. الجنسية |  |  |  |  |  |  |
|  | يمكن استخدام النظام الإلكتروني الجديد لتحديد الاحتياجات الخاصة للفئات الضعيفة التالية |  | | | | | |
|  | 1. اللاجئون - النازحون - المهاجرون |  |  |  |  |  |  |
|  | 1. أشخاص ذوي الإعاقة |  |  |  |  |  |  |
|  | 1. الفئات المهمشة والضعيفة الأخرى |  |  |  |  |  |  |
|  | يساعد النظام الإلكتروني الجديد في تقييم الفروق في حالات المرض والوفيات على أساس نوع الجنس. |  |  |  |  |  |  |
|  | يمكّن للنظام الإلكتروني الجديد المرضى من اتخاذ قرارات مستنيرة تتعلق بحالاتهم الصحية. |  |  |  |  |  |  |
| هل هناك أي خصائص غير متوفرة في النظام الإلكتروني الجديد؟ إذا كانت الإجابة بنعم، يرجى التحديد؟ | | | | | | |  |
| هل هناك أي خصائص تحتاج إلى تحسين؟ إذا كانت الإجابة بنعم، يرجى التحديد؟ | | | | | | |  |
| بالمقارنة مع الوثائق الورقية، ما هي فوائد النظام الإلكتروني الجديد؟ بالنسبة لمُقدّم الخدمات الصحية، بالنسبة للمريض؟ | | | | | | |  |
| هل تعتقد أن النظام الإلكتروني الجديد يحسن تقديم الخدمات الصحية؟ يرجى التحديد؟ | | | | | | |  |
| ما هي توصياتك لتحسين خصائص النظام الالكتروني؟  الوصول الى الخدمات:  الخصائص الفنية ونطاق العمل:  الاستخدام من قبل مقدمي الخدمات الصحية:  خصائص التوثيق وإعداد التقارير | | | | | | |  |
