# Supplementary material for: Evaluation of a piloted digital reproductive health registry in Jordan to improve mother and child health
Source: Reprod Health. 2025 May 31;22(Suppl 1):77. doi: 10.1186/s12978-025-01995-2 (PMC12125747; doi:10.1186/s12978-025-01995-2)
Supplement: Supplementary file 10 — Supplementary material 10. Focus group discussion (FGD) guide for stakeholders (in Arabic language) [file 12978_2025_1995_MOESM10_ESM.docx]

**إنشاء سجل منسق للصحة الإنجابية في الأردن**

**لتحسين صحة الأم والطفل**

**تقييم نقطة النهاية**

**استبيان للمعنيين**

**المعلومات الديموغرافية:**

**التاريخ:**

**البريد الإلكتروني لمقدم الخدمة:**

**(ملاحظة: هذه المعلومات هي لغرض التواصل معك فقط. سيتم حذفها أثناء مرحلة التحليل، كما لن يكون هناك ذكر لأي اسم أو أي معلومات أخرى.)**

**رمز مقدم الخدمة:**

**الجنس:**

- **ذكر**
- **أنثى**

**أعلى درجة علمية حصلت عليها (اختر واحدة فقط)**

أ. دكتوراه

ب. ماجستير

ج. دبلوم عالي

د. بكالوريوس

ه. شهادة دبلوم

و. غير ذلك (الرجاء التحديد..........................................)

**المؤسسة:**

**القسم:**

**الوظيفة:**

**منذ متى وأنت في وظيفتك؟**

| **الرقم** | **السؤال** | **الجواب** |
| --- | --- | --- |
|  | بشكل عام، كيف تقيم النظام الإلكتروني الجديد؟ |  |
|  | بشكل عام، كيف تقيم النظام الإلكتروني الجديد مقارنة بالسجلات الورقية المستخدمة في الماضي؟ |  |
|  | ما هي فوائد وآثار إدخال وتنفيذ نظام السجل الموحد للصحة الإنجابية الإلكتروني الجديد؟ |  |
|  | هل تعتقد أن النظام الجديد يحقق الهدف الرئيسي المتمثل في سد الفجوة المعلوماتية بين مختلف مستويات الرعاية؟ وكيف؟ |  |
|  | في رأيك، ما هي أهم سمات أو مكونات نظام السجل الموحد للصحة الإنجابية الإلكتروني الجديد؟ |  |
|  | في رأيك، ما هي أهم التحديات لاستخدام نظام السجل الموحد للصحة الإنجابية الإلكتروني الجديد؟ |  |
|  | هل تعتقد أن نظام السجل الموحد للصحة الإنجابية الإلكتروني الجديد قد زاد من جودة الرعاية الصحية للنساء والأطفال؟ يرجى التوضيح؟ |  |
|  | هل تعتقد أن نظام السجل الموحد للصحة الإنجابية الإلكتروني الجديد فعال من حيث التكلفة؟ يرجى التوضيح |  |
|  | هل تعتقد أن النظام الجديد يمثل أداة مستدامة يمكن أن تسهم في التدابير المتخذة لتحسين نتائج الصحة الإنجابية؟ |  |
|  | هل تعتقد أن النظام الجديد يمثل أداة مقبولة لأصحاب المصلحة المعنيين ولمقدمي ولمستخدمي الخدمات؟ لماذا؟ |  |
|  | هل تعتقد أن النظام الجديد يمثل آلية يمكن تعميمها واعتمادها على المستوى الوطني؟ |  |
|  | ما الذي يجعل النظام الجديد آلية يمكن تعميمها واعتمادها على المستوى الوطني؟ |  |
|  | هل تعتقد أن استخدام النظام الجديد كأداة لتوفير بيانات قابلة للتنفيذ في الوقت المناسب لمقدمي الخدمات الصحية من شأنه أن يحسن الرعاية المقدمة لصحة الأم والطفل؟ وكيف؟ يرجى التوضيح؟ |  |
|  | هل تعتقد أن النظام الجديد سوف يحسن رضا المريض عن الخدمات التي يتلقاها؟ كيف؟ يرجى التوضيح؟ |  |
|  | هل تعتقد أن تنفيذ نظام السجل الموحد للصحة الإنجابية الإلكتروني الجديد قد أثر على قرار الإحالة على مستويات مختلفة داخل مرفق الرعاية الصحية؟ |  |
|  | هل تعتقد أن نظام السجل الموحد للصحة الإنجابية الإلكتروني الجديد يؤدي إلى نظام إحالة أكثر فعالية وكفاءة مع خدمات أخرى؟ وكيف؟ يرجى التوضيح |  |
|  | هل تعتقد أن النظام الجديد يسهل انتاج المعلومات والتقارير المطلوبة لصحة الأم والطفل؟ وكيف؟ |  |
|  | ما هي نقاط الضعف/التحديات التي تحد من استخدام نظام السجل الموحد للصحة الإنجابية الإلكتروني الجديد؟ |  |
|  | هل هناك أي خصائص تحتاج إلى تحسين؟ إذا كانت الإجابة بنعم، يرجى التحديد؟ |  |
|  | أسئلة حول تكامل النوع الاجتماعي |  |
|  | هل يعمل النظام الإلكتروني الجديد على تحسين جمع وتحليل البيانات المصنفة حسب نوع الجنس مقارنة بالنظام الورقي؟  أسئلة استكشافية:  إذا كانت اجابتك نعم، يرجى وصف كيفية القيام بذلك.  إذا كانت اجابتك لا، يرجى اقتراح كيف يمكن ضمان ذلك؟ |  |
|  | هل يقوم النظام الإلكتروني بجمع وتحليل البيانات المصنفة حسب نوع الجنس (البيانات التي يتم جمعها حسب الجنس والعمر والحالة الاجتماعية والاعاقة ومكان الاقامة وغيرها من البيانات الشخصية للفرد)؟  أسئلة استكشافية:  إذا كانت اجابتك نعم، يرجى وصف كيفية القيام بذلك.  إذا كانت اجابتك لا، يرجى اقتراح كيف يمكن ضمان ذلك؟ |  |
|  | هل يساعد النظام الإلكتروني الجديد في تحديد الثغرات في إمكانية الوصول بين المجموعات المختلفة؟  - (رجال، نساء، بنين وبنات)  - (متزوج / غير متزوج)  - (المراهقة / سن الإنجاب / سن الأمل).  أسئلة استكشافية:  إذا كان الأمر كذلك يرجى وصف كيف مساعدة النظام لهذا الشيء.  إذا لم يكن الأمر كذلك، يرجى اقتراح كيف يمكن ضمان ذلك في نظام السجل المنسق للصحة الإنجابية الإلكتروني الجديد؟ |  |
|  | هل يساعد النظام الإلكتروني الجديد في تقييم الارتباط بين المحددات الاجتماعية والديموغرافية وامكانية الوصول / الاستفادة من الخدمات من أجل سياسات صحية أفضل؟  - التعليم  - الحالة الاقتصادية  - الجنسية  أسئلة استكشافية:  إذا كان الأمر كذلك، يرجى وصف كيفية مساعدة النظام لهذا الشيء.  إذا لم يكن الأمر كذلك، يرجى اقتراح كيف يمكن ضمان ذلك في نظام السجل المنسق للصحة الإنجابية الإلكتروني الجديد؟ |  |
|  | عند وضع السياسات لتكون أكثر شمولية للاجئين - النازحين داخلياً والمهاجرين والأشخاص ذوي الإعاقة، هل سيكون من المفيد أن يوفر لك النظام الإلكتروني مقارنات وتحليلات محددة؟  أسئلة استكشافية:  إذا كان الأمر كذلك يرجى وصف كيف مساعدة النظام لهذا الشيء.  إذا لم يكن الأمر كذلك، يرجى اقتراح كيف يمكن ضمان ذلك في نظام السجل المنسق للصحة الإنجابية الإلكتروني الجديد؟ |  |
|  | هل تعتقد أن النظام الإلكتروني الجديد يغطي جميع مكونات الصحة الإنجابية بطريقة مفيدة لك ولعملك؟ بما فيها:  - الأمراض المنقولة جنسيا/ فيروس نقص المناعة البشرية  - الصحة الجنسية  - العنف القائم على النوع الاجتماعي  -أخرى، الرجاء التحديد..................  أسئلة استكشافية:  إذا كان الأمر كذلك، يرجى وصف كيفية تغطية هذه المكونات.  إذا لم يكن الأمر كذلك، فيرجى اقتراح كيفية معالجة الثغرات ذات الصلة في نظام السجل المنسق للصحة الإنجابية الإلكتروني الجديد؟ |  |
|  | وأخيراً، هل هناك أي خصائص غير متوفرة في النظام الإلكتروني الجديد؟ إذا كانت الإجابة بنعم، يرجى التحديد؟ |  |
|  | هل لديك أي اقتراحات أو توصيات حول النظام؟ |  |
